# A New Combined Computational and Experimental Approach to Characterize Photoactive Conjugated 3D Polymers

**DOI:** 10.1002/smll.202407187

**Published:** 2025-02-05

**Authors:** Catherine Mollart, Patrick Heasman, Ellena Sherrett, Peter A. T. J Fletcher, Pierre Fayon, Jens M. H. Thomas, Villius Franckevičius, Michael J. G. Peach, Abbie Trewin

**Affiliations:** ^1^ Department of Chemistry Lancaster University Bailrigg Lancaster LA1 4YB UK; ^2^ Institute of Integrative Biology University of Liverpool Liverpool L69 7ZB UK; ^3^ Université Clermont Auvergne CHU Clermont Ferrand, Clermont Auvergne INP CNRS ICCF Clermont‐Ferrand F‐63000 France

**Keywords:** CMPs, electronic structure calculations, photo‐active materials, polymers, spectroscopy, structure‐property relationships, structure elucidation

## Abstract

A tractable new computational protocol is proposed to elucidate oligomeric‐scale detail from experimental spectra, providing insight into the local and longer‐range electronic and molecular structures of amorphous materials. The protocol uses an in‐house code Ambuild to grow kinetically‐controlled representative oligomeric clusters of an amorphous polymeric material. Generating many clusters, the statistical prevalence of different structural motifs is identified, and used to develop a ‘subset’ of structures that capture a broad range of important morphologies. Subsequent electronic structure calculations allow the prediction of IR, NMR, and UV–vis spectra of the bulk materials, providing significant insight into oligomeric scale topologies and helping develop structure–property relationships by identifying the underlying structural origins of different spectral features observed experimentally. Two known, and two novel, pyrene‐based conjugated microporous polymers (CMPs) are synthesized and characterized as a test bed for this newly‐proposed protocol. Meaningful IR, NMR, and UV–vis absorption spectral data, and experimentally comparable computationally derived spectra are obtained. Whilst IR and NMR reliably probe the local environment, UV–vis absorption spectroscopy is found to be particularly sensitive to the longer‐range structural motifs observed on an oligomeric scale, providing significant structural insight into the synthesized materials with reasonable computational cost.

## Graphical abstract

A new tractable computational protocol analyzes experimental spectra to illuminate the oligomeric scale structure of amorphous 3D polymers. Using Ambuild software, it generates representative molecular clusters, identifies common structural patterns, then calculates IR, NMR, and UV–vis spectra. The approach is validated against newly synthesised pyrene‐based conjugated microporous polymers (CMPs), with UV–vis proving especially useful at understanding longer‐range motifs.

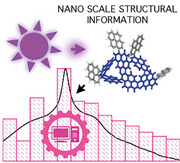

## 1 Introduction

**Figure 1:**
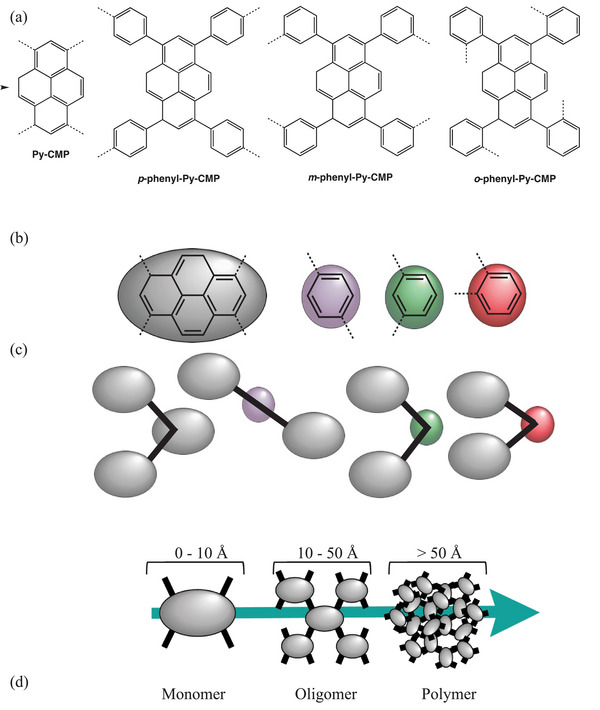
a) The CMP material polymer structures. b) The building units for the CMP materials, showing the attachment points (dashed lines) and the bonding topologies (solid lines). Grey ovoid – pyrene unit, purple ovoid – para connected phenyl, green ovoid –meta connected phenyl, and red ovoid – ortho connected phenyl. c) The connectivity topology of the CMP materials from left to right respectively, **Py**‐**CMP**, ***p***‐**phenyl**‐**Py**‐**CMP**, ***m***‐**phenyl**‐**Py**‐**CMP**, and ***o***‐**phenyl**‐**Py**‐**CMP**. d) The definition of length scales in relation to the monomeric building units used here. Monomer length scales are ≈0–10 Å, oligomeric length scales are 10–30 Å, and polymeric length scales are > 30 Å.

The importance of polymeric materials in the modern world is of course widely recognized. Beyond their commonplace societal use, polymers also underpin many existing technologies, and their novel use only looks set to expand. Despite the prevalence of polymeric materials, the characterization of their structure on an ‘oligomeric length scale’ (see **Figure** **1** for our definitions of length scales) remains a significant challenge due to the inherent disorder observed in most polymers. This is a particular challenge for 3D polymers, for example conjugated microporous polymers (CMPs)^[^ ^1^ ^]^ and porous aromatic frameworks (PAFs),^[^ ^2^ ^]^ where hyper cross‐linking occurs in all spatial directions, and network interpenetration (for increased packing efficiency) is commonplace. This characterization challenge is becoming increasingly detrimental to the development and realization of new polymeric materials with specific properties, as it prohibits a thorough understanding of the connections between monomer and synthetic strategy adaptations and the resultant polymeric properties.

As an example, consider materials that participate in generating or storing useful forms of energy, which are increasingly important due to our rising reliance on clean, renewable energy sources. In this context, materials that can convert solar energy, and so are photoactive, are a key part of any strategy directed toward harnessing solar energy for practical use. Numerous tactics can be adopted, including direct conversion to electricity (photovoltaics), to drive chemical reaction as photocatalysts (PC), and within photo‐electrochemistry (PEC). These applications all rely upon precise control of nanoscale structure to enable its subsequent use. E.g., the optical band gap was fine tuned in a library of CMP materials (CP‐CMPs) through precise statistical copolymerization,^[^ ^3^ ^]^ and the molecular detection sensitivity of TCB‐CMP was increased in comparison to a linear polymer analog.^[^ ^4^ ^]^

The local oligomeric‐scale structure of a photoactive polymer is thus vitally important. First, molecular conformation can influence the wavelength and intensity at which light is absorbed, affecting the overall efficiency and performance of the polymer. Second, it can direct oligomeric‐scale blending, and thus interactions between the donor polymer and the accepting moieties, which in turn can shape the ability to separate charge and avoid recombination. Finally, it can impact the larger‐scale structural features including phase separation and the resulting blend morphology that enables charge carrier pathways to reach their respective charge collectors.

We therefore need robust ways of probing the oligomeric‐scale structure of photoactive polymers. This is extremely challenging due to the limited characterization techniques available for polymeric materials, which tend to be formed in kinetically‐driven processes that result in considerable amorphousness and an overall lack of long‐range order, as confirmed by powder X‐ray diffraction (PXRD) techniques. While meaningful monomer‐scale characterization can be undertaken using techniques including nuclear magnetic resonance (NMR), X‐ray photoelectron spectroscopy (XPS), and Fourier‐transform infrared (FTIR),^[^ ^5^, ^6^, ^7^ ^]^ approaches that probe longer‐range oligomeric conformation are extremely challenging due to the intrinsic absence of long‐range order in these amorphous materials.

New families of polymer materials are being explored for solar energy conversion applications, including conjugated polymers,^[^ ^8^ ^]^ due to their ability to interact with light (photo activity), significant potential for chemical functionalization, and high photochemical stability, which offer benefits over traditional inorganic classes of materials.^[^ ^9^, ^10^, ^11^ ^]^ Thus, the ability to analyze the oligomeric scale structure of amorphous polymer systems is becoming increasingly important.

To aid in the structural analysis of amorphous polymers, computational approaches have been adopted to assess structure at the local, monomeric level. The sensitivity of predicted properties to the underlying local molecular structure means it is important to ‘follow the chemistry’ of a reaction to generate structures representative of the overall polymer network, particularly (as considered here) in cases where the product is formed in a kinetically‐driven process, yielding substantial structural diversity on a longer‐length scale.

We previously reported the ‘artificial synthesis’ of CMPs using *N*,*N*‐dimethylformamide (DMF) as solvent, where we modeled the molecular structure using our in‐house code Ambuild.^[^ ^12^, ^13^ ^]^ In this case, Ambuild allowed us to follow the full synthetic procedure, including the Sonogashira–Hagihara catalytic pathway, and the experimental stoichiometry of reagents for CMP‐1.^[^ ^12^, ^13^, ^14^, ^15^, ^16^, ^17^ ^]^ An Ambuild‐based approach thus can computationally mimic the synthetic growth process, producing a range of representative amorphous models of computationally tractable size, that each typify different local environments observed in the amorphous whole.

For optically active materials, additional data can be acquired through UV–vis absorption or fluorescence spectra. As the wavelength at which light is absorbed/emitted is directly related to the electronic structure, itself heavily influenced by the underlying molecular structure, it can be directly correlated to the oligomeric‐scale configuration of a molecular system. Indeed, UV–vis spectroscopy has been used to confirm the molecular conformation of dyes and to explore a polymer energy landscape.^[^ ^18^, ^19^ ^]^ Importantly, within highly conjugated systems, molecular orbitals are often delocalized beyond individual monomer units and are of oligomer scale. UV–vis spectroscopy therefore has the potential to be significant in probing oligomeric‐scale structure, allowing the generation of a picture of longer‐scale topologies in a material that would not be realizable using conventional techniques.

As a test case, we focus in this work on a set of pyrene‐based CMPs. These are relatively chemically simple highly‐conjugated photo‐active polymers, first introduced in 2011 by Jiang et al.^[^ ^20^ ^]^ We reasoned that these CMPs would enable us to probe oligomeric‐scale topological influences on the UV–vis spectra. In reference 19, Jiang et al. demonstrated control of the band gap of their CMP materials through the introduction of linear co‐monomers (or ‘linkers’) of increasing size. In particular, a pyrene‐based CMP was produced via the homocoupling of 1,3,6,8‐tetrabromopyrene using Yamamoto carbon‐coupling (Figure S1 and Scheme S1a, Supporting Information),^[^ ^21^ ^]^ previously referred to as **YPy** by Cooper and co‐workers, here referred to as **Py**‐**CMP**.^[^ ^20^, ^21^, ^22^, ^23^ ^]^ Similarly, co‐coupling of 1,3,6,8‐tetrabromopyrene with (*para*) 1,4‐dibromobenzene resulted in ***p***‐**phenyl**‐**Py**‐**CMP**, referred to as **YDBPy** by Jiang and co‐workers (Scheme S1b, Supporting Information).^[^ ^20^ ^]^ The inclusion of an effectively ‘linear’ co‐monomer gave rise to a reduction in surface area (**YPy**, 1508 m^2^ g^−1^; **YDBPy**, 1069 m^2^ g^−1^) and an increase in the (optically measured) band gap: **YPy**, 1.84 eV; **YDBPy**, 2.05 eV.^[^ ^20^ ^]^

Due to the challenging nature of analyzing amorphous materials, where multiple simulation models are required to generate a realistic ‘snapshot’ of the real‐world material, and where the cost and complexity of analysis scale rapidly with increasing system size, current approaches to assessing the origin of the UV–vis optical properties rely on the use of small, potentially unrepresentative fragments of the polymer structure (consisting of oligomers of only a few monomer units), where only a restricted number of topological isomers can be realized. This means both localized and longer‐range influences on electronic structure properties can be lost, as important but diverse structural features are necessarily missed. The consequence being that design strategies may be based on incomplete ‘rules’, where structure–property relationships are not fully realized or understood, inhibiting the discovery of new materials. Zwijnenburg et al.,^[^ ^24^ ^]^ addressing some of these deficiencies, extended to fragments consisting of 4 to 6 oligomers, were able to identify ring‐strain as a potential significant influence on the spectral properties.

In contrast to previous models, an ideal snapshot would consider much larger fragments, consisting of larger oligomers, allowing a broader range of structural diversity than can be realized from small model systems. Generation of multiple such fragments would allow the statistical composition of the possible array of structural arrangements to be realized, where in a small non‐representative set, the relative prevalence (and possibly even the features themselves) would be missed. Further, alternative oligomeric arrangements of polymers that give rise to similar characterization and analysis results can be explored, enabling a broadening of the structure–property relationships and better‐informed future material design.

With present methods, the underlying structural origins of the UV–vis spectra thus remain unclear, as multiple local structural features, i.e., local structure, topology, macrocyclic rings (MCRs), that can impact upon the UV–vis spectra and broader optical behavior, are either missed or misrepresented. With a more sophisticated approach, the combination of multiple inter‐dependent factors, presenting a far more complicated picture, can potentially be understood.

To directly address these limitations here, we use a combined computational and synthetic approach to elucidate the structural origins of the optical properties of **Py**‐**CMP** and ***p***‐**phenyl**‐**Py**‐**CMP,** whilst also validating our structural models against IR and solid‐state NMR data. We also expand the series of **phenyl‐Py‐CMP** by reporting the first synthesis of ***m***‐**phenyl**‐**Py**‐**CMP** and ***o***‐**phenyl**‐**Py**‐**CMP**, shown in Figure 1 and Scheme S1c,d respectively (Supporting Information). Expanding the series to include the meta and ortho phenyl linkers means we can explore structural‐directing effects. It is hypothesized that smaller pyrene–phenyl–pyrene angles, shown by the connectivity topology in Figure 1b, will direct toward macrocyclic ring formation. Therefore, greater numbers of rings should be observed for ***o***‐**phenyl**‐**Py**‐**CMP** than for ***m***‐**phenyl**‐**Py**‐**CMP** and ***p***‐**phenyl**‐**Py**‐**CMP** respectively, and thus we can elucidate to what extent such structural features do indeed play a role in controlling the observed optical properties.

Our approach provides monomeric and oligomeric‐level insight into the origin of the observed properties of these materials through a statistically meaningful exploration of the range of topological features present within larger oligomeric clusters of these polymers. We use this statistical analysis to produce simulated IR, solid‐state NMR, and UV–vis spectra. We demonstrate that very similar IR and NMR spectra can be acquired for very different oligomer topologies, suggesting these characterization techniques provide valuable local information but cannot be used to distinguish oligomeric‐length scale structural features. We also uniquely show that of the spectroscopic techniques studied, only UV–vis spectra can be used to assess the longer oligomeric‐scale structural features present within these polymers, and hence rationalize the respective properties.

## 2 Experimental Section

### 2.1 Experimental Synthesis

All CMPs reported were synthesized via the Yamamoto coupling reaction.^[^ ^21^ ^]^ Nickel(0)bis(cyclooctadiene)_2_ (Ni(cod)_2_), 2,2’‐bipyridine (bipy), and cyclooctadiene (cod) were used in stoichiometric quantities for each reaction. 1,3,6,8‐Tetrabromopyrene was combined in a 1:2 ratio with the 1,4‐dibromobenzene, 1,3‐dibromobenzene, and 1,2‐dibromobenzene co‐monomers to generate ***p***‐**phenyl**‐**Py**‐**CMP, *m***‐**phenyl**‐**Py**‐**CMP,** and ***o***‐**phenyl**‐**Py**‐**CMP,** respectively.

A 250 mL two‐neck round‐bottomed flask, a 100 mL single‐neck round‐bottomed flask, a two‐directional tap, and two Suba‐seals were placed in dry N_2_ within a glovebox. Ni(cod)_2_ (1.43 g, 5.2 mmol), cod (0.67 mL, 5.3 mmol), and bipy (0.81 g, 5.2 mmol) were added to the 250 mL round‐bottomed flask, then the Suba‐seal and tap were connected accordingly to either neck of the flask. Additionally, the single‐neck 100 mL round‐bottomed flask and Suba‐seal were used for the monomers (1,3,6,8‐tetrabromopyrene (514 mg, 1 mmol) and 1,3,6,8‐tetrabromopyrene (257 mg, 0.5 mmol) with 1,4‐dibromobenzene (234 mg, 1 mmol), 1,3‐dibromobenzene(234 mg, 1 mmol), and 1,2‐dibromobenzene (234 mg, 1 mmol) for synthesis of **Py**‐**CMP, *p***‐**phenyl**‐**Py**‐**CMP**, ***m***‐**phenyl**‐**Py**‐**CMP,** and ***o***‐**phenyl**‐**Py**‐**CMP** respectively). Both flasks were then taken from the glovebox to the fume hood. The flasks were both re‐evacuated and refilled with N_2_ or Ar gas. DMF (50 mL) was added to the nickel reagent vessel, and the solution heated to 80 °C for 1 h. In the meantime, DMF (50 mL) was added to the vessel containing the monomers, and the mixture left to stir for 1 h to facilitate dissolution of the reagents. The monomer solution was added to the nickel reagent solution via syringe, and the mixture heated at 80 °C for 24 h. The reaction was allowed to cool to room temperature, and concentrated hydrochloric acid (15 mL) was added dropwise. The mixture was allowed to stir for 1 h. The solid product was collected by suction filtration and washed on the filter with CHCl_3_ (3 ´ 100 mL), H_2_O (3 ´ 100 mL), and THF (3 ´ 100 mL). Further purification was carried out via Soxhlet extraction with THF for 24 h. The solid product was then dried in vacuo at 120 °C. The mass of each product obtained was 334 mg, 220 mg, 147 mg, and 100 mg for **Py**‐**CMP, *p***‐**phenyl**‐**Py**‐**CMP**, ***m***‐**phenyl**‐**Py**‐**CMP,** and ***o***‐**phenyl**‐**Py**‐**CMP**, respectively.

### 2.2 Experimental Characterization

Solid‐state ^13^C NMR spectra were collected using a Bruker Avance 400 wide‐bore NMR spectrometer. UV–Vis spectra were collected using an Avantes AvaSpec‐2048FT spectrometer with a ThorLabs SLS201/M stabilized fiber‐coupled light source. Fourier‐transform infra‐red spectra of the CMP samples were collected using an Agilent Cary 630 FTIR spectrometer.

### 2.3 Artificial Synthesi**s**

Previous studies have shown that amorphous polymer networks are generated in a stepwise process. First, oligomers form that increase in concentration until a gel is formed. These polymer gels continue to grow, and the networks interpenetrate to eventually form insoluble polymer particles that drop out of solution.^[^ ^13^, ^25^ ^]^ The crowded, non‐reversible covalent bond formation process pins individual monomer units in a kinetic structure as the polymer is forming, meaning that a wide distribution of structural features form, potentially in topologies far from the thermodynamic minima.^[^ ^13^ ^]^ Therefore, small, unconstrained fragments of polymer in their thermodynamic minima (calculated through unconstrained structural optimization) are unrepresentative of such a polymer network, as they represent an idealized synthetically unrealizable scenario that is statistically unlikely in the real system.

**Figure 2:**
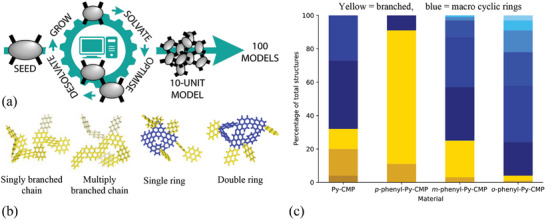
a) Schematic showing the approach used within Ambuild to generate 100 individual models of a 10‐unit cluster. A single build unit, shown here for **Py**‐**CMP**, is seeded within a simulation cell. Solvent is added and the structure optimized. Following optimization, the solvent is removed, and a grow step undertaken within which another build unit is added to the cluster. The new cluster is re‐solvated, and an optimization step is undertaken within which a molecular dynamic run is followed by a *zip* test to check for intra‐cluster bonding. This loop continues until there are ten units within the cluster, at which point a new cluster is started. b) Examples of the 10‐unit clusters showing some of the structural types observed for **Py**‐**CMP**. Rings are shown in blue and branches in yellow (darker and lighter yellow for different branches). c) Chart showing the percentage of each structural type for each of the CMP materials. Yellow = branched (palest yellow = singly branched, mid yellow = doubly branched, darkest yellow = multiply branched), blue = MCRs (darkest blue = single MCRs, increasing through double, triple, quadruple, and quintuple to lightest blue = sextuple rings). Full breakdown of the structural types can be found in Table S1 (Supporting Information).

To properly represent the polymer chemical structure, a statistically relevant number of fragment models that are large enough to realize macrocyclic features, are required. Here the Ambuild code is used to generate 100 representative fragments of each polymer system in the form of 10‐Py monomer unit clusters, as shown in **Figure** **2**a. As a proof of concept study, a balance is required: Between the size of each cluster; a large enough cluster is required to incorporate larger scale features to appropriately model the extended polymer structure, with the tractability of the system; if each cluster is too large then their analysis becomes prohibitively computationally expensive. A 10‐unit cluster size was chosen as this was deemed a good balance of size and computational expense. Similarly, a balance is required between having a large enough number of repeats to be statistically meaningful and being too large that it becomes unachievable in a meaningful timeframe. Thus, a combination of 100 repeats of 10‐unit clusters was deemed to be structurally and statistically meaningful to appropriately sample the structural diversity present for the purposes of this study. Likewise, here we do not include the catalytic step used previously in Ambuild artificial synthesis strategies, as the computational expense of their inclusion would have limited the number and size of the oligomer models. Further work will be required to fully validate and optimize these choices in terms of compromising between computational expense and gaining the most meaningful information about the system of study.

Ambuild is a GPU‐based simulation code written in Python specifically designed to model amorphous porous polymers.^[^ ^12^, ^26^ ^]^ Ambuild integrates with HOOMD‐blue,^[^ ^27^, ^28^ ^]^ which was used as our molecular mechanics‐based geometry optimization and molecular dynamics (MD) engine throughout. The polymer consistent forcefield^[^ ^29^ ^]^ (PCFF) was used throughout, as it is well‐established this forcefield is appropriate for modeling microporous organic polymers, such as the pyrene‐based CMPs described here.^[^ ^12^, ^30^, ^31^, ^32^ ^]^

The artificial synthesis procedure is described in depth in Section S2 (Supporting Information), but to summarize; initially, one tetrabromopyrene building block is seeded into the center of a cubic Ambuild simulation cell. The remaining cell volume is filled with DMF solvent molecules to better replicate the synthetic conditions, and the simulation cell then undergoes geometry optimization and molecular dynamics steps. The DMF solvent is then removed from the cell, and in all cases apart from **Py**‐**CMP** itself, two phenyl linkers (representing the experimental stoichiometry) are bonded to existing building blocks within the system using Ambuild *growBlocks* steps, and the solvent re‐seeded into the cell. An Ambuild *zipBlocks* step is then undertaken to allow additional bond formation between close building blocks (based on pre‐defined proximity criteria), followed again by geometry optimization and MD steps. The process is repeated, this time growing with a tetrabromopyrene building block in all cases. This is all then iterated to build clusters of up to ten pyrene building blocks (and twenty linkers).

To account for the amorphous nature of each of the four CMP materials investigated, and to achieve statistical relevancy, 100 independent clusters of each of the four materials, were grown. Each resultant cluster was categorized according to its basic morphology, based on the underlying structural motifs it contains. The categorization focuses on the prevalence of macrocyclic ring‐like formations, which are representative of cross‐linking within the extended polymer structure. As such, the three broad categories defined were: linear, branched (and the extent of any branching: singly‐, doubly‐, or multiply‐), and macrocyclic rings (MCRs, and the number of MCRs observed: clusters containing one, two, three, four, five, and six rings were all observed). Note that because of the focus on MCR‐formation and the extent of crosslinking, the presence of any MCRs in the categorization eclipses the presence of chains; in a typical structure containing MCRs, chains are also present.

Some examples of **Py**‐**CMP** singly‐ and multiply‐branched chains, and single and double MCRs, are given in Figure 2b. This relatively broad categorization (effectively a ‘dominant motif analysis’) allows to statistically consider the prevalence of differing structural motifs, based on how frequently they manifest amongst each set of 100 repeats.

Figure 2c shows the categorization of the 100 clusters for each polymer assessed, colored yellow for chains and blue for MCRs. For ***p***‐**phenyl**‐**Py**‐**CMP, *m***‐**phenyl**‐**Py**‐**CMP,** and ***o***‐**phenyl**‐**Py**‐**CMP** a clear trend is seen toward increasingly prevalent MCR formation as the pyrene–phenyl–pyrene angle decreases, as expected. Interestingly, the same ratio of branched MCR structures for **Py**‐**CMP** as for ***m***‐**phenyl**‐**Py**‐**CMP**, which we attribute to the similar effective angle between the pyrene nodes, is observed. This suggests the hypothesis that a decreased pyrene–phenyl–pyrene angle directs toward MCR formation is correct. Further, this will allow to assess the contribution of MCRs and hence crosslinking to the optical behavior.

To generate a more manageable set of clusters for further structural refinement and analysis, a representative subset of ≈10 of the 100 ten‐pyrene clusters, sampling the behavior of the full set, was chosen for each of the materials. The choice of representative subset clusters was based on ensuring at least one observed sample of a structural motif was included in each case, and that multiple examples of highly prevalent motifs were also included. This enabled to sample each distinct structural type observed for each material, while also approximately accounting for the statistical prevalence of each structural type within the full set of 100 clusters. Details of the specific clusters chosen for each material are given in Section S3 (Supporting Information).

### 2.4 Computational Characterization

For the four materials, their IR, NMR, and UV–vis spectroscopic properties are considered based on electronic structure calculations, using as a starting point the representative subset cluster geometries generated using Ambuild. During work‐up, unreacted bromine end groups that are accessible will be removed leaving only a small amount. Therefore, any remaining unreacted bromine atoms was replaced with hydrogen atoms, to more realistically represent the very small number of bromine atoms expected in a cluster of this size (weight‐percent arguments suggest 1 to 2 bromine atoms in clusters of this size due to the high degree of polymer crosslinking yielding ≈4 wt.% unreacted bromine end groups in the experimental synthesis).^[^ ^1^, ^33^ ^]^

In the development of the protocol, techniques that are computationally tractable were chosen deliberately; a search is in progress for an approach that yields useful information at a ‘reasonable’ computational expense, rather than searching for the highest possible accuracy, appropriate when modeling amorphous materials such as CMPs, which are a kinetic product rather than the minimum energy product obtained under thermodynamic control.

Grimme and co‐workers’ GFN2‐xTB semi‐empirical tight binding approach,^[^ ^34^, ^35^ ^]^ as implemented in their freely available XTB program,^[^ ^36^ ^]^ was used as an efficient electronic structure‐based geometry optimizer. This methodology provides a low‐cost geometry optimization that is readily applicable to systems with hundreds/thousands of atoms, which produces structures of an accuracy approaching those from conventional density‐functional theory (DFT) calculations. We consider this step purely a ‘relaxation’ of the structure, to yield more sensible bond lengths, conjugation, and intramolecular interactions (such as e.g., p–p stacking interactions) than those provided by the basic force‐field approach, without imposing the inherent restrictions present in a molecular mechanics approach. These relatively subtle structural details do influence the electronic structure of the clusters and any conjugation across the system, and hence the molecular properties evaluated using them.

This same model chemistry is also used to generate the IR spectroscopic data (the harmonic vibrational normal modes and IR intensities). Despite the kinetically driven structures produced, all of the relaxed representative subset clusters are found to be minima on their respective potential energy surfaces.

To test the applicability of the XTB‐derived geometries in subsequent NMR and UV–vis spectroscopic analyses over the use of conventional DFT‐based geometries, the impact on several test systems was considered. In preliminary comparisons we made between B3LYP geometries calculated using the cc‐pVDZ basis set, GFN2‐xTB, and the PCFF‐based geometries obtained from Ambuild, it is found that for both the NMR and UV–vis data calculated, the impact of using the XTB‐derived geometries is minimal compared to the underlying influence of model chemistry choice on the NMR and UV–vis properties themselves, but there is a larger difference compared to using Ambuild (PCFF) structures. A careful consideration of the use of the GFN2‐xTB geometry versus the CCSD/cc‐pVTZ geometry of molecular Py shows a near‐negligible influence; see Section S4 (Supporting Information) for further details.

^13^C NMR shielding constants were evaluated on each of the representative subset clusters for each of the four systems. Here, the PBE/pcS‐1 model chemistry was chosen; the pcS‐1 basis set is specifically designed for shielding constant evaluation and is readily tractable on systems of this size, and the use of a GGA functional is often advantageous in NMR calculations.^[^ ^37^ ^]^ These calculations were all carried out using Gaussian 09.^[^ ^38^ ^]^ To obtain chemical shifts from the calculated shielding constants (to facilitate comparison with experimental NMR data) a calibration was performed against a range of small molecules (including tetramethylsilane (TMS), Py, etc.) as outlined in Section S4 (Supporting Information). As a result, all calculated shielding constants were shifted against a reference of 180 ppm, which is fully consistent with the observed shift required to match the experimental solid‐state NMR data obtained for the four CMP systems.

UV–vis spectra were similarly mimicked using the same representative subsets of clusters, by calculating vertical time‐dependent DFT (TDDFT) excitation energies and oscillator strengths within the Tamm–Dancoff approximation (TDA), which is both cost‐effective,^[^ ^39^ ^]^ and known to be beneficial in obtaining reliable state ordering of excitation energies in highly conjugated molecules, including molecular pyrene.^[^ ^40^ ^]^ Here, the CAM‐B3LYP/def2‐SVP model chemistry is used. CAM‐B3LYP^[^ ^41^ ^]^ is chosen explicitly to avoid any spurious low‐energy charge‐transfer states contaminating the spectra, which are otherwise likely to be a problem in extended systems such as these. As with the NMR, these calculations were all performed using Gaussian 09.

It is anticipated that however that this choice of model chemistry and approach will lead to an overall overestimation of excitation energies relative to experiment. Indeed, this is what is observed for molecular Py. Basis set incompleteness has a non‐negligible effect but is a necessary consequence of reducing computational cost for these large systems; however, its impact seems to be fairly uniform for the energy ranges considered here, i.e., it impacts the excited states considered reasonably uniformly. The use of the standard vertical approximation, whereby the structure is fixed at the ground state structure upon excitation, also has a large (≈0.3 eV) influence on the low‐lying excited states due to the not insignificant structural reorganization observed in Py upon electronic excitation.^[^ ^42^ ^]^ With all these factors in mind, a difference between the calculated and experimental excitation energies of at least 0.8 eV was anticipated; but for the energy range of interest, a fairly uniform shift between calculated and experimental values. These factors are discussed in detail in Section S4 (Supporting Information).

For each of the representative clusters, 50 singlet excited states and their associated oscillator strengths were calculated. The energy range covered extends to at least 4.5 eV in every case, well above the relevant experimental energy range, even when accounting for the necessary calibration shift.

### 2.5 Computationally‐Generated Spectra

The full IR, NMR, and UV–vis absorption spectra are computationally modeled for each material. This provides this work with a holistic view of the data generated by examining the overall match to experiment, probing all peak positions and intensities, rather than focusing on individual states/contributions. It also allows the work to investigate the predictive power of the computational approach in producing meaningful, experimentally representative spectra.

To generate IR, NMR, and UV–vis spectra for each individual representative cluster, the raw calculated data was taken, any necessary calibration was applied, and then the spectral data was broadened to more realistically mimic the experimentally observed spectra. For more details on this process, see Section S5 (Supporting Information).

The spectral data obtained for individual clusters were combined, to produce ‘average’ (where each cluster spectrum contributes equally), or occasionally ‘weighted average’ (where the contribution of each spectrum is weighted to emphasize or de‐emphasize the particular contributions of individual clusters) spectra. Using this approach, a single spectrum can be produced for each classification of cluster. This enables this work to consider the contribution of different structural motifs to the overall spectra produced. This approach is also used to produce overall spectra that reflect the statistical behavior anticipated in the bulk material for direct comparison to experiment. However, it is noted that this comparison is reliant upon the modeling capturing the range of structural diversity present, their respective contributions to the spectra, and the frequency with which these structural features occur.

## 3 Results and Discussion

### 3.1 Py‐CMP

**Figure 3:**
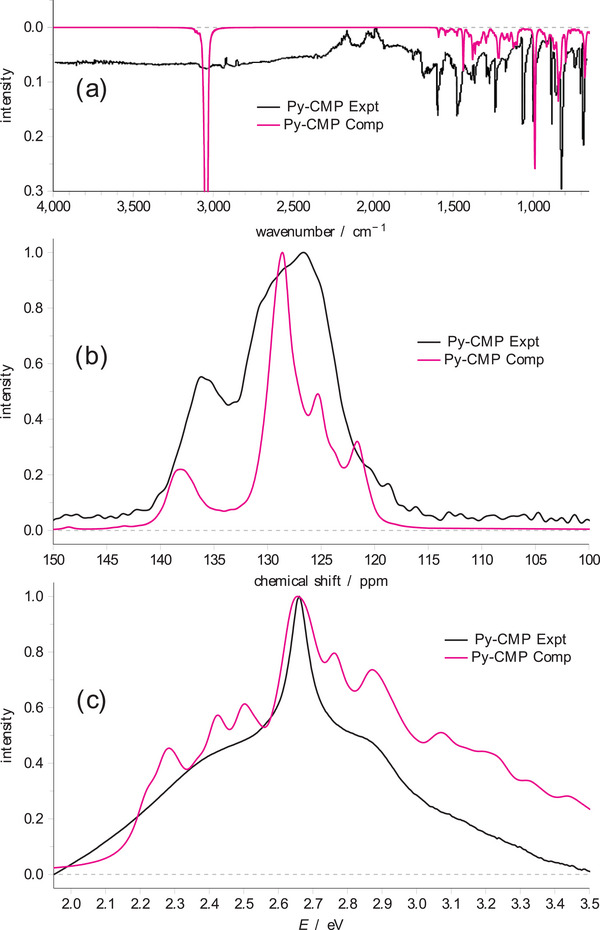
The experimentally derived a) IR, b) NMR, and c) UV–vis spectra for **Py**‐**CMP**, compared to the computationally generated spectra, created here using an average across cluster models.

The experimental IR, solid‐state ^13^C NMR, and UV–vis spectra for **Py**‐**CMP** are shown in **Figure** **3**. Each spectrum is consistent with that reported previously.^[^ ^20^ ^]^ The key features in the IR spectrum all occur below 1600 cm^−1^; above this wavenumber, only a broad, nondescript signal at 3050 cm^−1^ is observed, potentially indicating atmospheric gases and/or water absorption within the **Py**‐**CMP** as well as different C–H environments. We therefore focus on the ‘fingerprint’ region. The spectrum is dominated by key peaks at ≈680, 800, 875, 1000, 1075, 1200, 1450, and 1600 cm^−1^. For the solid‐state ^13^C NMR spectrum, there are two broad peaks observed. The first is a significant peak with a maximum at ≈127 ppm, covering a range of ≈10 ppm. The second is a smaller peak, of roughly half the height of the main peak, with a maximum at ≈137 ppm. The UV–vis spectrum in contrast has only very broad features. Here, the maximum occurs at 2.65 eV, with a relatively sharp shoulder to higher energy, at ≈2.8 eV, and a broader shoulder to lower energy, at ≈2.4 eV.

As a first attempt at rationalizing these experimental spectra, we consider the calculated spectra obtained from some simple 10‐pyrene chain models, where the pyrene units are joined either systematically or randomly. We consider three models, first where the pyrenes are joined at the 1‐, and 6‐ position, this configuration affords the least steric hinderance, second where the pyrenes are joined at the 1‐, and 8‐ positions, and third where the pyrenes are joined in a random combination of 1‐, 3‐, 6‐, and 8‐ positions. The spectra are calculated as outlined in Section S6 (Supporting Information). Although these are clearly simple models and are not representative of the amorphous nature of the full **Py**‐**CMP** polymer, they provide useful information.

The IR, NMR and UV spectrums based on the linear 10‐pyrene system are shown in Figure S13A (Supporting Information). They reproduce the majority of the key experimental IR details within the fingerprint region well; some of the relative intensities do not match perfectly with the experimental spectrum, but all key peaks (except for the peak at 1075 cm^−1^, which we will return to) are present. At higher wavenumbers, the calculated spectra show very significant C–H stretching peaks at ≈3050 cm^−1^. We attribute this discrepancy in part due to the likelihood of physisorbed water in the experimental material masking this region of the spectrum, and the overestimation of such intensities as predicted by electronic structure methods.^[^ ^16^ ^]^ That said, it may also be possible that we are over‐emphasizing the presence of C–H bonds within **Py**‐**CMP**; if our clusters are underestimating the amount of crosslinking, then we would overestimate the number of C–H groups. We know that we are overestimating the number of end groups within the clusters, due to their finite size, so this might also be having an effect.

With regard to the solid‐state ^13^C NMR spectrum, the linear 10‐pyrene systems are similarly informative, and reproduces the basic behavior of the system quite effectively. There are two peaks, roughly centered at the same chemical shifts as in the experimental spectrum, with the peak at ≈127 ppm being broader and more intense. The smaller secondary peak, albeit with reduced intensity compared to experiment, is immediately reproduced from the model system. Further analysis reveals that the secondary peak at ≈137 ppm arises from the C centers that are part of those C–C bonds connecting individual Py units; i.e., the origin of the side peak is the polymerization process itself.

Finally, with our linear 10‐pyrene model systems, we consider the UV–vis spectrum. By some margin, the lowest excitation energy is the most allowed in the 10‐pyrene chains, such that in each case, the spectrum is effectively one (fairly narrow) peak. There is a shift of ≈0.1 eV between the two cases. Unlike in the case of the IR and NMR spectra, here the linear 10‐pyrene models do not reproduce the experimental UV–vis spectrum. This highlights an intrinsic sensitivity of UV–vis spectral data to longer‐scale structural differences that is not observed with IR and NMR data, which we will see repeatedly throughout this work. For NMR and IR, the information is either localized to specific chemistries, e.g., distinguishing between phenyl and pyrene centers, or is not reliant on the oligomeric‐scale environment. However, UV–vis data can give information about the oligomeric‐scale, as excitations depend on electronic structure effects extending across multiple monomer units. Thus, it can give insight into structural motifs, including rings, branching, and polymer densities, on the oligomeric scale and which more closely represent the real polymer framework structure.

Considering the chain model that is randomly generated, we are able to systematically vary the chain length to consider the effect of chain truncation on the resulting spectra, shown Figure S13B,C (Supporting Information). By a visual assessment, we can see that in all spectra the change with each additional unit becomes smaller and can be considered reasonably converged at 10 units. To test the convergence, we generated a 15‐unit cluster which visually looked very similar to the 10 unit cluster, shown in Figure S13D (Supporting Information). To quantify the convergence, we undertook a “distance” comparison between the spectra calculated for each of the 1–10‐unit cluster models and the 15 unit cluster model, shown in Figure S13E (Supporting Information).^[^ ^43^ ^]^ In each spectra, convergence between models can be observed by 10 units and so we therefore believe that 10 units provides a suitable compromise between computational cost and minimizing termination effects.

#### 3.1.1 Cluster Models

To represent the anticipated underlying amorphous morphology of the **Py**‐**CMP** system more realistically, we considered a series of cluster models. While it is impossible to replicate wholly the infinite amorphous polymer framework, by following the chemistry and the structural rules thus imposed and replicated through the molecular mechanics description and structural optimization, we can be assured that the clusters are sensible representations of possible structural arrangements.

For **Py**‐**CMP**, we use a subset of 11 clusters to represent the 100 clusters generated via Ambuild. Each cluster was ‘grown’ and classified using the procedure outlined in Section S2 (Supporting Information), and subsequently characterized as described in Section S4 (Supporting Information). Finally, the spectra based on each cluster were generated as described in Section S5 (Supporting Information).

In this case, the 11 clusters we consider comprise four structures that contain no MCRs: one singly‐branched structure (this structure differs from the ‘linear’ models in that it is not systematically bonded despite exhibiting minimal branching); one doubly‐branched structure; and two multiply‐branched structures. The remaining seven clusters considered all contain MCRs; four each containing a single MCR, and three of which each contains two MCRs; this suggests some degree of polymer crosslinking is occurring in these clusters. Initially, we consider the variation of each property with cluster type. To ease this comparison, we produce average spectra to group all the contributions from a particular cluster type.

For the IR spectra, there is overall only a small variation in the behavior between different cluster types. The relative intensities of some of the peaks shift (particularly for the MCR, where the region between 800–1000 cm^−1^ is notably affected), but in each case the same peaks are identifiable. As such, it is difficult to suggest whether one cluster type is more prevalent over another in the experimental material. An average spectrum, which takes into account each cluster in the subset does a good job overall of reproducing the experimental IR spectrum, but it does not represent a notable improvement over the basic linear 10‐Py models.

In the case of the solid‐state ^13^C NMR spectrum, we see again a relatively small influence of the structural diversity; in all cases, we reproduce the two key peaks in the spectrum. The structural diversity does seem beneficial in adding to the width of the most intense peak, and the presence of MCRs does seem to add to the region ≈133 ppm (i.e., between the two peaks), which improves overall correlation with experiment.

For the UV–vis spectra, we see more pronounced effects. Branching gives rise to multiple peaks with significant intensity, with the (unnormalized) intensity of the dominant peak of a branch roughly correlating to the number of pyrene units in that branch. In most cases, branching results in higher excitation energies as compared to the linear 10‐Py model systems.

The clusters with MCRs give rise to more varied excitation energies and intensities. Here, these clusters do also exhibit some branching, alongside the MCRs. We see overall increased intensity to higher energy in this case, consistent with branching, and also some lower excitation energies, consistent with the presence of MCRs. The averaged spectrum based on those structures containing only a single MCR is an excellent approximation to the experimental spectrum, in a way the linear 10‐pyrene models were not. We are also able to distinguish the presence of a 3‐pyrene MCR observed in a single cluster, as this gives rise to the lowest observed excitation energies of any of the models.

We note that we observe this particularly small and strained MCR in only one of the cluster models, and thus although this clearly has a significant influence on the excitation energies associated with that cluster, the contribution of such motifs to the overall UV–vis spectrum of the **Py**‐**CMP** is expected to be relatively small. Even though the growth of the **Py**‐**CMP** is kinetically controlled, in the presence of solvent we anticipate that such small, strained MCRs will be statistically less likely to form than larger, more stable MCRs. The final spectra, averaging over the data for the 11 clusters from the subset, for IR, NMR, and UV–vis are shown in Figure 3 as the ‘computationally‐derived’ spectra.

We note that even with this more sophisticated approach, we still do not pick up a peak at 1075 cm^−1^ in the IR spectrum of **Py**‐**CMP** (and several other less prominent features to lower wavenumber). However, this is near the region of the spectrum where we would anticipate an aryl C–Br stretch to appear^[^ ^44^, ^45^ ^]^; we therefore suggest that this discrepancy relates to our model clusters removing all traces of Br, whereas in the experimental system, some small amounts remain (≈4 wt.% bromine); see Section S10 (Supporting Information) for further details.

At this stage, it appears that the UV–vis spectra are most sensitive to the underlying structures used and demonstrate the considerable benefit of modeling the spectroscopic properties of the **Py**‐**CMP** amorphous system using Ambuild‐generated clusters.

### 3.2 Steering MCR Formation

To further investigate the nature of amorphous CMP structures and their connection to MCR formation/crosslinking, the utility of our protocol outlined above for the prediction of IR, NMR, and UV–vis spectra in such amorphous CMP materials, and the subsequent elucidation of the underlying molecular structure properties, we now considered the formation of a series of three additional **Py**‐**CMP**‐based materials, where we introduce a ‘linker’ as a second monomer into the reaction; these are shown in Figure 1.

***p***‐**Phenyl**‐**Py**‐**CMP** is constructed from 1,3,6,8‐tetrabromopyrene and 1,4‐dibromobenzene. This particular CMP has been previously reported as **YDBPy**.^[^ ^20^ ^]^ In this case, the bromine atoms on the co‐monomer are para to each other, meaning the linker acts as an effectively linear connector between Py units. As such, there is no directional change in the growth of the CMP, and the linker purely acts as a ‘spacer’.

Alongside ***p***‐**phenyl**‐**Py**‐**CMP**, we also consider the phenyl‐Py‐CMPs constructed with the (meta) 1,3‐dibromobenzene and (ortho) 1,2‐dibromobenzene co‐monomers, ***m***‐**phenyl**‐**Py**‐**CMP** and ***o***‐**phenyl**‐**Py**‐**CMP**, respectively. The effect of the *m*‐phenyl linker is to both space and redirect the direction of growth within the CMP by ≈60 degrees, and similarly the effect of the *o*‐phenyl linker is to redirect the direction of growth by ≈120 degrees. The use of these alternative co‐monomers may therefore direct the polymer structure to form a greater proportion of MCRs/more substantial crosslinking by effectively directing the growth of the polymer back toward the already polymerized pyrene units. As discussed previously, we anticipate MCR formation will likely be similar in ***m***‐**phenyl**‐**Py**‐**CMP**, relative to **Py**‐**CMP**, decreased in ***p***‐**phenyl**‐**Py**‐**CMP**, and increased in ***o***‐**phenyl**‐**Py**‐**CMP**.

**Figure 4:**
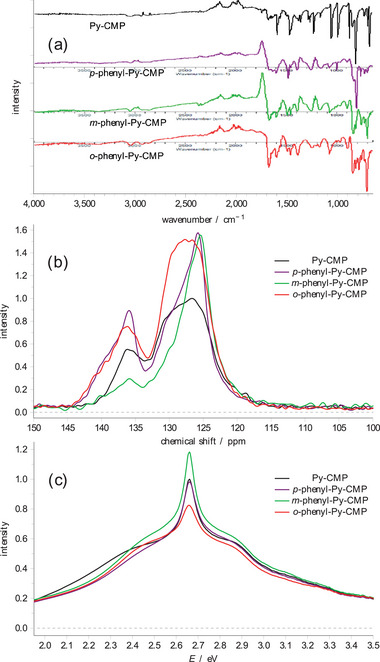
The experimentally derived a) IR, b) NMR, and c) UV‐vis spectra for ***p*‐**, ***m*‐**, and ***o***‐**phenyl**‐**Py**‐**CMP** compared to **Py**‐**CMP**, as synthesized for this study.

Experimental IR, NMR, and UV–vis spectra for each of these systems are shown in **Figure** **4**. The IR spectra contain many of the same features as for **Py**‐**CMP**, and again only show important details below 1600 cm^−1^, but it is clear that the spectra are distinct in that the relative intensities of the key peaks differ between the four systems.

The NMR spectra are notably distinct from each other in terms of relative intensity but show the same two sets of peaks as **Py‐CMP**. We interpret these as ‘additive’ features over those observed in **Py**‐**CMP**. In the case of the ***p***‐**phenyl**‐**Py**‐**CMP** and ***m***‐**phenyl**‐**Py**‐**CMPs**, there is a notable increase in observed intensity at lower ppm, skewing the shape of the main peak to be asymmetrically biased toward lower ppm. At the same time, we observe an increase in the width of the secondary peak toward higher ppm for the *p*‐ material. For the ***m***‐**phenyl**‐**Py**‐**CMP** case, the secondary peak diminishes in intensity. In the case of the ***o***‐**phenyl**‐**Py**‐**CMP**, we observe an increase in intensity throughout the main peak, and toward higher ppm in the secondary peak. See Section S7 (Supporting Information) for further details.

Notably however, the UV–vis spectra show only very minor differences between the four CMPs. The peak maximum is unchanged between the four materials; we observe relatively small changes in the shoulders to higher and lower energies, such that the spectrum of each material is distinct, but far more similar to each other than anticipated; an individual material would unlikely be recognizable on the basis of each UV–vis absorption spectrum. In particular, the linker‐containing CMPs all seem to exhibit a faster drop in intensity at lower energies (approaching 2 eV), and the shoulder to the lower energy side of the primary peak is less distinct / separated; the behavior to higher energies is again very similar between the four materials. Given our observations for **Py**‐**CMP** in terms of how sensitive the UV–vis behavior is to the details of the underlying molecular structure, and the pronounced nature of this sensitivity as compared to the IR and NMR behaviors, this is unexpected.

### 3.3 Clusters of the Co‐Polymers

To further probe these observations, we have initially adopted a similar computational approach to that taken for **Py**‐**CMP**, whereby we generated 100 clusters each of ***p***‐**phenyl**‐**Py**‐**CMP**, ***m***‐**phenyl**‐**Py**‐**CMP**, and ***o***‐**phenyl**‐**Py**‐**CMP**, where each cluster contains 10 pyrene units alongside 20 linker units following the ideal stoichiometry from the reaction scheme.

In the case of ***p***‐**phenyl**‐**Py**‐**CMP**, we observe only two structural types amongst all 100 clusters: linear branched clusters, and branched structures containing a single MCR. This suggests that the introduction of the *p*‐linker has the effect of reducing/inhibiting MCR formation, and hence reduces crosslinking. Although we anticipated the introduction of this linker would reduce strain in any MCRs present, as it acts as a spacer, it has in fact reduced MCR formation and hence crosslinking in general. We observe the linker often forms ‘chains’ itself within the clusters, suggesting that, at least in our computational synthesis route, linker–linker bonding is commonplace. Full details are given in Section S3 (Supporting Information). Here therefore, our representative subset consists of eight multiply branched structures, and only two structures each containing a single MCR.

Alternatively for ***m***‐**phenyl**‐**Py**‐**CMP**, the linker does appear to bias the system to produce an increased number of MCRs. This is both indicated by the significant increase in the number of structures overall that contain MCRs over **Py**‐**CMP**, but also the prevalence of multiple MCRs being observed within those individual structures. In this case, clusters containing up to five MCRs are observed, suggesting a significantly more crosslinked structure overall. Full details are again given in Section S3 (Supporting Information). Here, our representative subset consists of nine clusters that contain MCRs, varying from those containing a single MCR, to those containing five MCRs. Alongside this are three multiply branched structures.

Finally, for the ***o***‐**phenyl**‐**Py**‐**CMP**, the linker acts to produce even more MCRs than in the ***m***‐**phenyl**‐**Py**‐**CMP** case. Here, clusters containing up to six MCRs are observed, and the prevalence of structures containing clusters again increases, to the point that our representative subset contains only one structure without any MCRs (a multiply branched structure) and ten structures that each contain between one and six MCRs. Again, this suggests very significant cross‐linking within the extended ***o***‐**phenyl**‐**Py**‐**CMP** structure.

#### 3.3.1 *p*‐phenyl‐Py‐CMP

**Figure 5:**
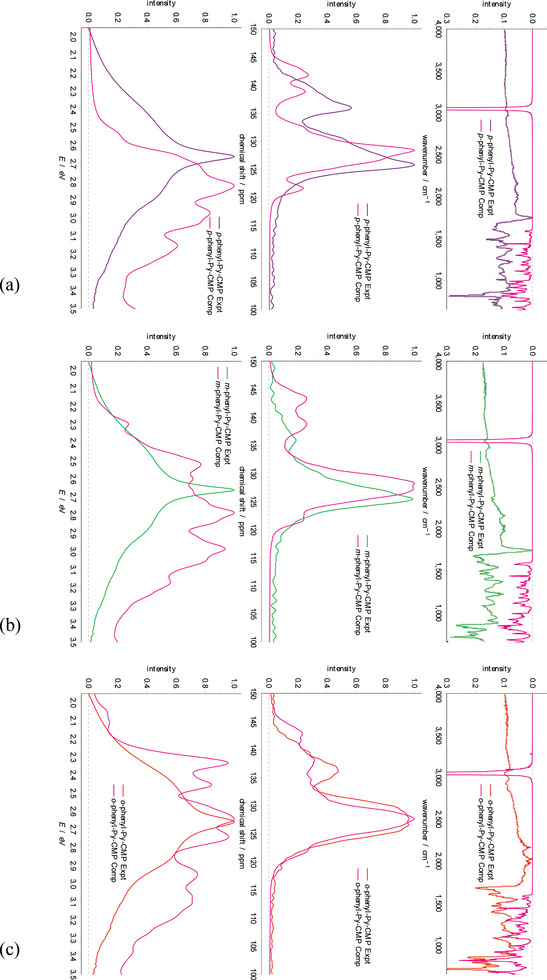
The experimental and calculated IR, NMR, and UV–vis spectra for a) ***p***‐**phenyl**‐**Py**‐**CMP**. b) ***m***‐**phenyl**‐**Py**‐**CMP** and c) ***o***‐**phenyl**‐**Py**‐**CMP**. In each case the computational spectra is an average over cluster types, considering the linker fully.

Let us initially focus on the ***p***‐**phenyl**‐**Py**‐**CMP** case, shown in **Figure** **5**a. Here, for the IR spectra, we observe nearly identical spectra averaged over the MCR clusters and over the multiply branched clusters, the net result being the system is equally well represented by either the branched or MCR structures, which suggests that the MCRs (and hence crosslinking) are relatively unimportant in this case, consistent with their reduced prevalence overall and their relatively unstrained nature.

As with the **Py**‐**CMP** case, the average IR spectrum is able to reproduce some of the key features observed in the experimental spectrum (notably the 1075 cm^−1^ peak is still missing, again consistent with it arising due to C–Br stretches). However, the overall agreement with experiment is poorer than in the case of **Py**‐**CMP**.

Similarly for the NMR data, there is only a small difference between the average spectrum predicted from the MCRs, and from the multiply branched clusters. Here however, it is far more immediate that the agreement with the experimental data is poorer than with the **Py**‐**CMP** case. This is primarily due to the secondary peak at higher ppm itself splitting into effectively two small peaks. We will return to this observation.

For the UV–vis data, we do see a more pronounced difference between the average spectrum for the MCRs, versus the average spectrum for the multiply‐branched clusters. Notably, the MCR spectrum is shifted to higher energy compared to the multiply‐branched case, in contrast to the behavior observed previously for **Py**‐**CMP**. This is perhaps reflective of a decrease in conjugation length observed within these clusters with an MCR present, and the lack of strain within the MCRs formed compared to in **Py**‐**CMP**.

Importantly, there is a significant difference between the UV–vis spectrum as predicted from the average across all clusters for ***p***‐**phenyl**‐**Py**‐**CMP** as compared to **Py**‐**CMP**. The net result is poor agreement between the experimental and computational spectra in this case—recall that there is only a very small difference observed experimentally, as in Figure 4. This poor agreement is also true of the average MCR spectrum, and the average multiply branched spectrum, and so is not indicative of an imbalance in the statistics underlying the cluster structures generated.

#### 3.3.2 *m*‐phenyl‐Py‐CMP

Next, we consider a similar analysis for ***m***‐**phenyl**‐**Py**‐**CMP**, shown in Figure 5b. Again, we begin by considering the IR spectra predicted for each cluster type. In this case, there are some more pronounced differences between the spectrum as predicted by each cluster type; notably the peaks below 900 cm^−1^ are particularly affected. Overall, however, the spectra are relatively consistent and could not be used to distinguish clearly between the presence or absence of MCRs/crosslinking. As with ***p***‐**phenyl**‐**Py**‐**CMP**, the agreement between the average spectrum and experiment is less clear than was observed with **Py**‐**CMP**.

Despite the increased structural diversity observed in the ***m***‐**phenyl**‐**Py**‐**CMP** case, we again see a relatively small change between the NMR spectra of each cluster type; there are no distinguishing features that can be correlated with the presence or absence of MCRs within the structure. As with the ***p***‐**phenyl**‐**Py**‐**CMP** case, the agreement with experiment is relatively poor, and again this is due to the higher ppm/more downfield secondary peak being poorly reproduced.

For the UV–vis data in this case, we observe significant differences based on the cluster type; due to the larger variation in cluster types for ***m***‐**phenyl**‐**Py**‐**CMP**, the differences do not fall into any clear‐cut categories. It is evident however that because of the significant differences observed between each cluster type, our statistical approach to producing a ‘final’ spectrum results in a vastly different spectrum as compared to picking any individual cluster type. This again emphasizes the relative sensitivity of the UV–vis data, as compared to the NMR and IR data, in picking up intrinsic structural differences.

As with ***p***‐**phenyl**‐**Py**‐**CMP**, the agreement with the experimental UV–vis spectrum is poor, as we again predict a significant shift in the UV–vis spectrum relative to both **Py**‐**CMP** and ***p***‐**phenyl**‐**Py**‐**CMP**.

#### 3.3.3 *o*‐phenyl‐Py‐CMP

Finally, we consider the ***o***‐**phenyl**‐**Py**‐**CMP**, shown in Figure 5c. In this case, the IR spectrum predicted is more sensitive to the structural types; given this system has the most diverse structural types of all of those considered, this is perhaps not surprising. Again though, the key peaks are present across all structure types, and it is just the relative intensities that are influenced overall. In terms of the comparison of the average spectrum to experiment, we observe a similar level of agreement to that seen in the ***p*‐** and ***m***‐**phenyl**‐**Py**‐**CMP** cases.

Once again, the NMR spectra across the different structural types show relatively minor variation. Interestingly, we observe that the region ≈135 ppm becomes more saturated as the density of MCRs increases within a cluster. This feature is also apparent in the spectra of ***m***‐**phenyl**‐**Py**‐**CMP**, although the trend is less pronounced. This perhaps suggests that a more densely crosslinked structure would yield a greater signal in this region, indicating that our clusters are in general underestimating the amount of crosslinking observed overall, due to their finite sizes and thus an overabundance of end‐group effects. The same overestimation to higher chemical shift of the secondary peak is still observed in this case, albeit to a lesser extent than in the ***p*‐** and ***m***‐**phenyl**‐**Py**‐**CMP** cases.

In terms of the UV–vis data, as with ***m***‐**phenyl**‐**Py**‐**CMP**, we again see a significant sensitivity of the spectrum predicted to the underlying structural type. Here, at least for those clusters with a particularly high proportion of MCRs (those with four and above), the lowest excitation energy drops as the number of MCRs increases. Thus, as with ***m***‐**phenyl**‐**Py**‐**CMP**, in this case the choice and weighting of individual structural types is of vital importance in terms of the final spectrum produced. As with the other systems involving linkers, however, we again predict a significant shift in the UV–vis spectrum relative to that observed experimentally.

### 3.4 Improving Agreement with Experiment

As outlined above, the agreement between experiment and the computational spectra for those systems incorporating a linker (the ***p*‐**, ***m*‐**, and ***o***‐**phenyl**‐**Py**‐**CMP** systems) is considerably poorer than for the raw **Py**‐**CMP**. Indeed, one could not expect to identify the respective copolymer systems from their respective computational spectra based on structures generated with their idealized stoichiometry.

Does this relate to the different prevalence of structural types within the subsets; are we overpredicting one type of structure and underpredicting another type of structure?

Changes in the weightings of individual structural types across the systems cannot account for the poor agreement with experiment, as the spectral properties associated with each structural type differs across individual systems. That this occurs across all forms of spectroscopy considered, and that it cannot be explained by the changes in the composition of the representative subsets, suggests the underlying individual cluster structures are less representative of the experimental morphology observed in ***x***‐**phenyl**‐**Py**‐**CMP** than for the **Py**‐**CMP** case.

Notably, this is entirely consistent with the observed similarities in the experimental UV–vis spectra of the four materials, particularly given we have found the UV–vis spectra to be the most sensitive to the underlying structure of the clusters considered. The experimental UV–vis data therefore suggests the newly synthesized ***x***‐**phenyl**‐**Py**‐**CMP**s are (possibly significantly) less distinct from the original **Py**‐**CMP** than our clusters suggest.

To help elucidate the potential overestimation of the importance of the linker in the ***x***‐**phenyl**‐**Py**‐**CMP** cases, we initially consider the NMR data. Unlike with the IR and UV–vis data, we can readily assign spectral intensity as originating from either the Py units, or the linker units.

**Figure 6:**
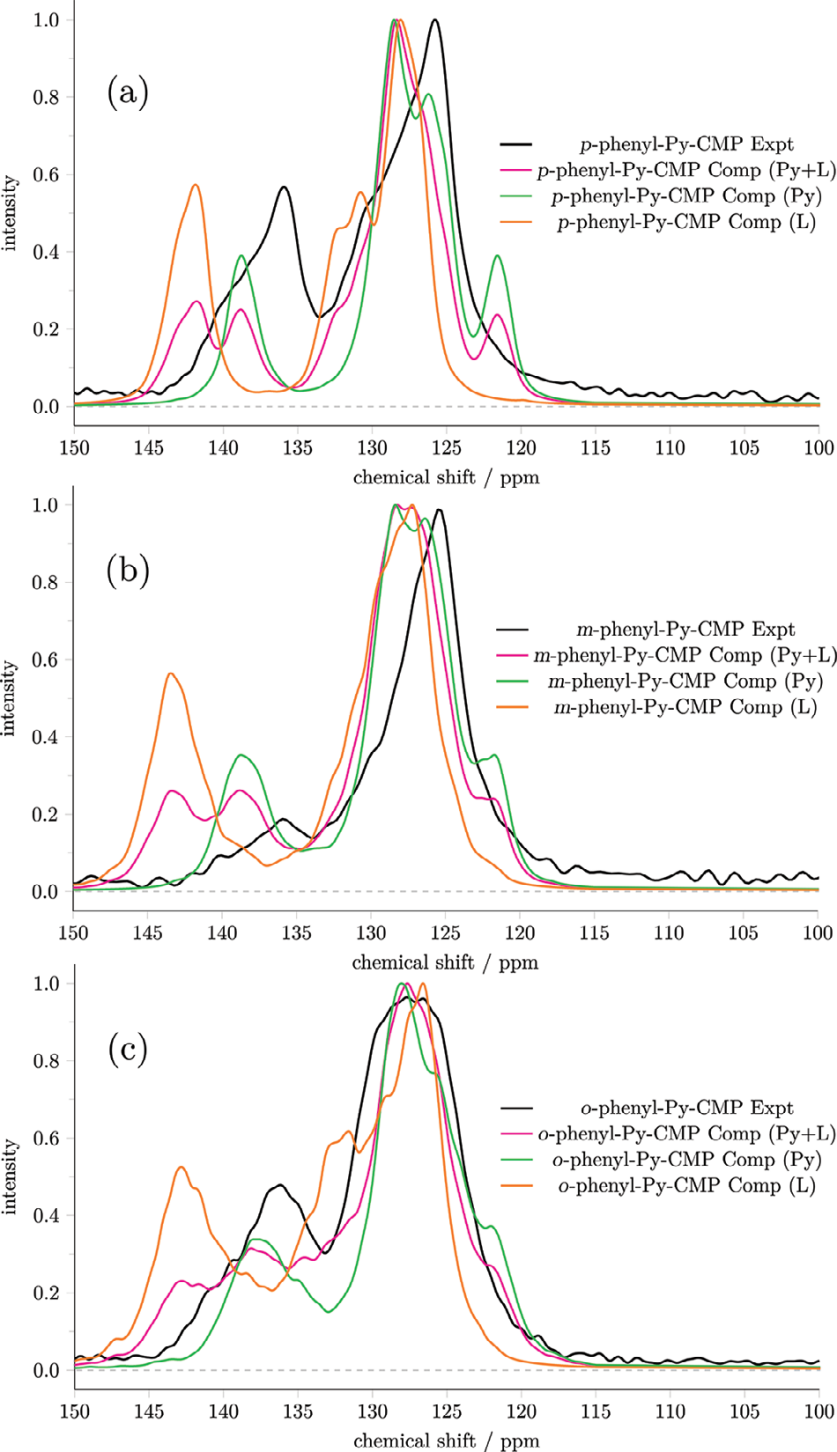
Experimental and computational NMR for a) ***p*‐**, b) ***m*‐**, and c) ***o***‐**phenyl**‐**Py**‐**CMP** systems. The computationally derived NMR spectrum in each case is divided into contributions within each cluster as arising from both pyrene and linker (Py+L), just pyrene (Py), and just linker (L).

**Figure** **6** shows the NMR spectra, by structural type, as originating from both Py and linker, and individually from the Py units, and from the linker units, in ***x***‐**phenyl**‐**Py**‐**CMP**s. For each material, we observe that the agreement with experiment is substantially improved when only considering contributions originating from the Py units, compared to the case where linker contributions are also included. Similarly, where we show only linker contributions to the spectra, we see very poor agreement with experiment. In particular, the distortion to higher chemical shift of the secondary peak is entirely caused by the presence of linker signals. These observations suggest that, in all cases, we are overestimating the amount of linker present in the structures. This is also consistent with an analysis of equiv. structural types (for instance, multiply‐branched structures) across all four materials.

We proceed with this assumption, that we have overestimated the extent to which the linker is incorporated into the structure within the Ambuild‐generated clusters. Given the clusters were generated with a 2:1 ratio of linker to Py (following the experimental stoichiometry), this seems likely. There are multiple ways in which the Py and linker monomers can be incorporated that result in very similar overall characterization data. These include (i) as idealized with a repeating pyrene–linker bonding within the polymer (idealized co‐polymer), (ii) with a random but homogenous distribution of pyrene–pyrene, pyrene–linker, or linker–linker bonding within the polymer (fully integrated statistical co‐polymer), or (iii) regions with pyrene–pyrene, pyrene–linker, or linker–linker bonding within the polymer (block co‐polymer). Figure S15 (Supporting Information) illustrates these different forms. All three types may be present in varying ratios, and this may differ between ***x***‐**phenyl**‐**Py**‐**CMP**s.

**Figure 7:**
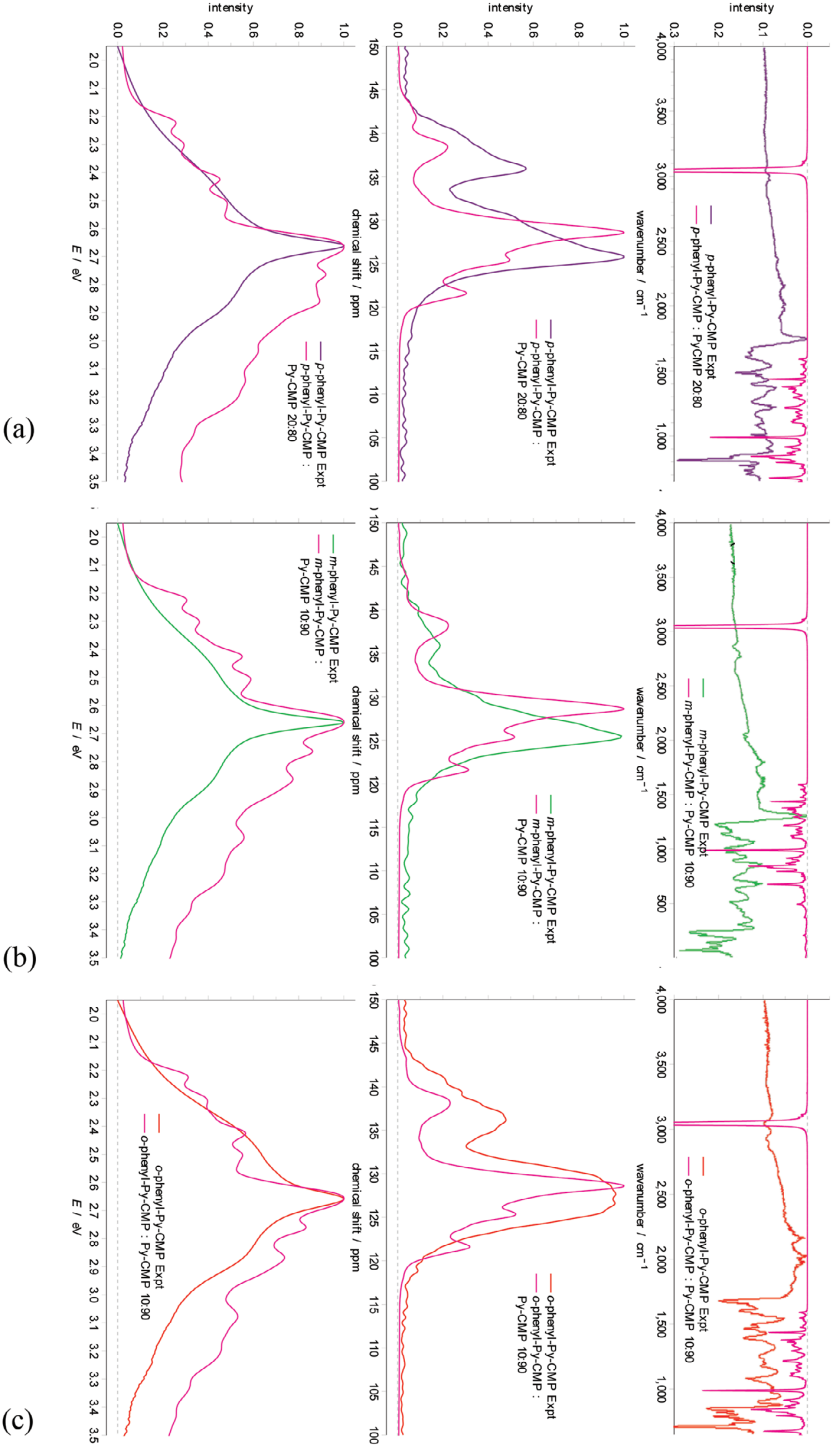
Experimental and computational spectra for combinations of ***p*‐**, ***m*‐**, and ***o***‐**phenyl**‐**Py**‐**CMP** with **Py**‐**CMP**. The best fit to experiment is 20:80 for ***p***‐**phenyl**‐**Py**‐**CMP** and **Py**‐**CMP**, and 10:90 for ***m***‐**phenyl**‐**Py**‐**CMP** and ***o***‐**phenyl**‐**Py**‐**CMP**, and **Py**‐**CMP**.

The clusters we have generated following the reaction scheme stoichiometry clearly best represent the second scenario above; a fully integrated statistical co‐polymer. To approximate a block co‐polymer computationally, we consider a weighted average of the **Py**‐**CMP** and ***x***‐**phenyl**‐**Py**‐**CMP** data for each spectral type. If, e.g., we assume that ***p***‐**phenyl**‐**Py**‐**CMP** consists of 90% **Py**‐**CMP**‐like regions, and 10% ***p***‐**phenyl**‐**Py**‐**CMP** regions, then we weight the respective cluster contributions in a 9:1 ratio. The sensitivity of each spectral type to this weighting is discussed in Section S8 (Supporting Information). We find that a weighting of between 10 and 20% produces NMR and UV–vis spectra best representative of the experimental ones, as shown in **Figure** **7**, suggesting that the integration of the Py monomer and each linker is considerably less than anticipated.

Overall, this highlights the extent to which clusters can be used to represent the amorphous nature of CMP‐like materials, and how our protocol can be used to predict, in a meaningful and insightful way, the spectral properties of such materials.

### 3.5 Retro‐Rationalization Fit to Experiment

Finally, we aim to apply the knowledge gained so far to extract structural information from the systems, and to assign the origin of peaks within the experimental spectra to specific structural types that are present within the system network.

Beginning with **Py**‐**CMP**, Figure S19 (Supporting Information) shows the individual IR spectrum of each cluster within the **Py**‐**CMP** subset. As highlighted previously, each IR spectrum is very similar, showing little to no sensitivity to structural type. Similarly, Figure S20 (Supporting Information) demonstrates that whilst there are slight shifts in the NMR spectrum depending on the structural type of each member of the **Py**‐**CMP** subset, no one spectrum is able to fully rationalize the experimental result, although the overall shape of each computational NMR spectrum is similar.

However, again as highlighted earlier, the UV–vis spectrum of each cluster in the **Py**‐**CMP** subset (Figure S21, Supporting Information) does change significantly depending on the structural features present. This suggests that only UV–vis (of the characterization methods studied) is sensitive enough to detect differences depending on the oligomeric‐structural details of the clusters. We therefore assess the total UV–vis spectrum of each material to establish which structural features appear dominant in the experimental spectrum.

Examining the molecular orbitals of individual clusters in the subsets for each material, we see orbitals delocalize across multiple building blocks, often in a complicated, unintuitive manner. This observation reiterates the importance of modeling large fragments to correctly replicate this behavior. As the excited states studied involve multiple significant contributions from different orbital transitions, we use ‘density‐difference plots’ to characterize the behavior of individual excited states. These plots, as shown in Figures S36–40 (Supporting Information), also often show delocalized behavior. In general, we find the more connected the fragment, as judged by a higher ‘saturation’ of building blocks reacted to each other, the more delocalized the excitations are. In contrast, terminal building blocks exhibit more localized excitations confined to effectively an individual Py monomer. This observation again suggests that, as the overall computational UV–vis spectrum is more intense at higher energies than in the experimental spectra, we are overestimating the terminal and branching contributions, which appear in this higher energy region. To improve our modeling approach in the future, larger clusters are required to minimize terminating group contributions, effectively allowing us to consider the system as an infinite set of MCRs.

Next, we consider the density of the cluster models in comparison to experiment (for full details, see Section S12, Supporting Information). As CMPs are amorphous, kinetically controlled frameworks, they are inhomogeneous throughout and can exhibit discrete regions of both high‐ and low‐density.^[^ ^31^ ^]^ We can identify how the density of the experimental material compares to our systems, by assessing the resulting influence of different densities on the calculated UV–vis spectra of our cluster models. We do this by beginning with the broadened UV–vis spectrum of the entire subset for each material, systematically removing the lowest density cluster one‐by‐one until we are left with just the highest density cluster in the subset.

**Figure 8:**
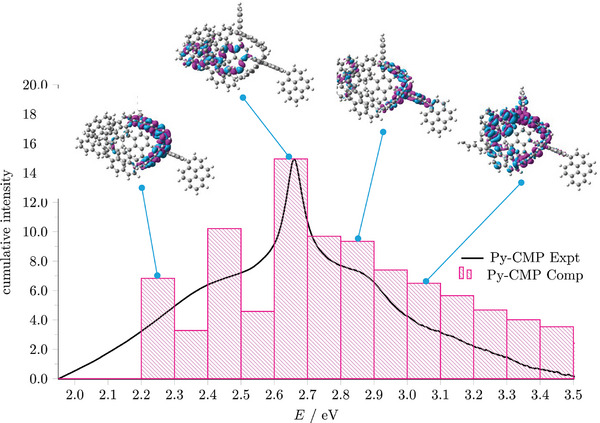
Histogram showing the cumulative intensity of the excited states within **Py**‐**CMP**, normalized to the maximum experimental intensity (black line). Insets show the density difference plots for an example excited state within selected energy bins. The magenta color represents the “electron”, where the electron has been excited to, and the cyan represents the “hole”; where the electron has been excited from.

For **Py**‐**CMP**, as the overall cluster density increases, the peaks within the UV–vis spectrum at lower energy become increasingly less dominant, whilst the peaks at higher energy remain throughout; the most intense peak at ≈2.7 eV is relatively constant. Figure S46 (Supporting Information) shows a histogram of the density (number) of excited states at each energy. Considering the respective oscillator strengths of the excited states within each energy bin of the histogram as a cumulative intensity, shown in **Figure** **8**, we can see that the overall shape of the histogram is reflective of the experimental spectra. Further, we can identify the origins of the peaks for each energy by assessing the structural features present that give rise to the excited states in each bin. Features at lower energies originate from a small number of strong‐intensity excited states, whereas those at higher energies originate from states that are more prevalent but with individually lower oscillator strengths. The main feature at ≈2.7 eV arises from a (very) small number of states, each with a dominant oscillator strength. We note the lower energy region of the spectrum is comprised of excited states localized on saturated, highly strained building blocks. In contrast, the higher energy region of the spectrum is generally composed of states involving less saturated and less strained building blocks.

We now assess the other three materials. For ***p***‐**phenyl**‐**Py**‐**CMP**, as the system density increases (Figure S47, Supporting Information), there is a better match between the computational and experimental UV–vis spectra. For ***m***‐**phenyl**‐**Py**‐**CMP**, the peak maximum again aligns better with experiment as the system density increases (Figure S49, Supporting Information); however, the increase in density also leads to an overestimation of peak intensity at higher energies. As for **Py**‐**CMP**, the peaks at low energies are ascribed to the lower‐density clusters. Finally, for ***o***‐**phenyl**‐**Py**‐**CMP**, the peak maximum aligns well throughout (Figure S51, Supporting Information), with the best match to experiment occurring with the highest system density; however, all the peaks in the spectrum are retained throughout, with greater resolution between each peak with increasing density.

Assessing the range of densities in each of the ***x***‐**phenyl**‐**Py**‐**CMP** materials, ***p***‐**phenyl**‐**Py**‐**CMP** is by far the lowest density material, whilst ***o***‐**phenyl**‐**Py**‐**CMP** is the densest. This can be rationalized by considering the structural directing effects of each linker, where the ortho linker corresponds to the most highly strained, densest material, and the para linker corresponds to the least strained, least dense material. These structural directing effects in turn impact the UV–vis spectra of each material.

Considering the histograms of the excited states of each modelled cluster relative to the experimental UV–vis spectrum; for ***p***‐**phenyl**‐**Py**‐**CMP**, we overestimate the energy of the peaks (Figure S48, Supporting Information); for ***o***‐**phenyl**‐**Py**‐**CMP**, we underestimate the energy (Figure S52, Supporting Information), and for ***m***‐**phenyl**‐**Py**‐**CMP**, we have the best overall match to experiment, with only a modest overestimation of the energy (Figure S50, Supporting Information).

## 4 Conclusions

We have proposed a carefully designed, tractable new computational protocol aimed at providing insight into the local and longer‐range electronic and molecular structures of amorphous materials. The protocol involves the use of our in‐house code Ambuild, which allows the direct computational mimicking of a synthetic procedure to generate representative oligomeric clusters of an amorphous material grown under ‘kinetic control’. The amorphous bulk is represented by growing a series of independent clusters, each mimicking a different part of the bulk. Through generating a large number of clusters, the statistical prevalence of different structural motifs can be identified and used to develop a ‘subset’ of structures that capture a broad range of important morphologies.

Electronic structure calculations run on these subset structures then allows the prediction of the IR, NMR, and UV–vis spectra of the bulk materials, providing significant insight into the molecular scale topology of the materials, and helping develop structure–property relationships by identifying the underlying structural origins of different spectral features.

In this work, we have synthesized and characterized two known, and two novel, CMPs, **Py**‐**CMP**, and ***p*‐**, ***m*‐** and ***o***‐**phenyl**‐**Py**‐**CMP**s, as a test bed for our newly‐proposed computational protocol. These systems have allowed us to consider the prevalence and importance of macrocyclic ring formation (and thus crosslinking) within the structures, whether the formation of such MCRs can be controlled through MCR‐directing linkers, and the impact of these underlying structural changes on the spectral properties of the materials via the characterization of their experimental IR, NMR, and UV–vis absorption spectral properties.

Based on representative subsets of Ambuild‐grown amorphous clusters of these four materials, we have demonstrated that electronic structure calculations can be used to generate meaningful IR, NMR, and UV–vis absorption spectral data, and experimentally comparable computationally derived spectra. Whilst IR and NMR reliably probe the local environment, UV–vis absorption spectroscopy is found to be particularly sensitive to the longer‐range structural motifs observed on an oligomeric scale, providing significant structural insight into the synthesized materials with reasonable computational cost.

For **Py**‐**CMP**, in all cases the predicted spectra match well with experiment, validating the computational protocol. For the three ***x***‐**phenyl**‐**Py**‐**CMP** systems, the agreement between calculated and experimental spectra is initially poor, reflecting an overestimation of the ‘mixing’ of Py and linkers in the clusters generated. Modifying the relative weightings of different clusters assuming an underlying morphology dominated by Py yields computational spectra that much better match experiment.

We have also identified a density‐dependence of the spectral properties, with those clusters of higher density generally correlating more reliably with the CMPs synthesized for this work. Again, UV–vis absorption data is key.

Although the cluster models show significant promise, it seems we underestimate the amount of polymer crosslinking in the Ambuild‐grown clusters, overemphasizing edge / end group effects. The impact of the finite size of the clusters is also unclear. We are presently investigating these factors, and our results will form the basis of a future study.

Overall, our new computational protocol has allowed us to probe the molecular structure of a series of CMPs, allowing us to identify underlying structural features that give rise to spectral properties in a greater level of detail than has previously been achieved for systems of this type. It highlights the importance of being able to replicate the structural diversity seen in an amorphous system, and the intrinsic power of mid‐sized cluster models. It also shows the significant value of UV–vis absorption as a probe of the electronic (and hence molecular) structure of CMPs in extended amorphous materials.

## Conflict of Interest

The authors declare no conflict of interest.

## Supporting information



Supporting Information

## Data Availability

The data that support the findings of this study are available in the supporting information of this article.
