# Supplementary material for: A New Combined Computational and Experimental Approach to Characterize Photoactive Conjugated 3D Polymers
Source: Small. 2025 Feb 5;21(9):2407187. doi: 10.1002/smll.202407187 (PMC11878256; doi:10.1002/smll.202407187)
Supplement: Supplementary file 1 — Supporting Information [file SMLL-21-2407187-s001.docx]

**Supporting Information**

A new combined computational and experimental approach to characterise photoactive conjugated 3D polymers

Catherine Mollart,^1^ Patrick Heasman,^1^ Ellena Sherrett,^1^ Peter A. T. J. Fletcher,^1^ Pierre Fayon,^1,3^ Jens M. H. Thomas,^2^ Vilius Franckevičius,^1^ Michael J. G. Peach^1^* and Abbie Trewin^1^*

^1^Department of Chemistry, Lancaster University, Bailrigg, Lancaster, UK, LA1 4YB

^2^Institute of Integrative Biology, University of Liverpool, Liverpool, UK, L69 7ZB

^3^Université Clermont Auvergne, CHU Clermont Ferrand, Clermont Auvergne INP, CNRS, ICCF, F-63000 Clermont-Ferrand, France

**Contents**

S1. CMPs 2

S2. Ambuild 4

S3. Statistical analysis 8

S4. Computational characterisation 11

S5. Spectral broadening 17

S6. Pyrene linear chain 19

S7. Experimental spectra 21

S8. Spectral weighting 23

S9. Degree of phase separation 25

S10. Cluster spectra compared to experiment 27

S11. Density-difference plots to characterise excited states 49

S12. Influence of density on UV–vis spectra 58

S13. References 65

1. **CMPs**

**Figure S1.** The Yamamoto catalytic cycle. R and R’ are aromatic monomers.^1^

**Scheme S1.** Reaction scheme used to synthesise (a) **Py-CMP**, (b) ***p*-phenyl-Py-CMP**, (c) ***m*-phenyl-Py-CMP** and (d) ***o*-phenyl-Py-CMP**. (a) and (b) were first reported by Jiang and co-workers as YPy and YDBPy, respectively,^2^ (c) and (d) were synthesised, to our knowledge, for the first time as part of this work. cod = cyclooctadiene, bipy = 2,2’-bipyridine, DMF = N,N’-dimethylformamide.

1. **Ambuild**

Ambuild is an in-house, GPU-based software specifically designed to model amorphous porous polymers such as CMPs, covalent triazine frameworks, porous aromatic frameworks and hypercrosslinked polymers.^3-7^ It is written in Python and able to integrate with HOOMD-blue,^8,9^ used as the geometry optimisation and molecular dynamics (MD) engine throughout. As in our previous studies employing Ambuild, we utilised the polymer consistent (PCFF) forcefield to describe bonding and non-bonding interactions in the Ambuild models as this was deemed the most appropriate for our systems.^10^ For further details on the Ambuild code, please see our previous publications (Reference set 3), where it is described in-depth, with full details and a validation of the approach, or its Github site (<https://github.com/linucks/ambuild/wiki>).

For each of the four materials investigated, **Py-CMP**, ***p*-phenyl-Py-CMP**,

***m*-phenyl-Py-CMP** and ***o*‑phenyl‑Py‑CMP**, we use the following computational protocol:

1. An initial tetrabromopyrene molecule is seeded into the centre of a cubic cell of size (60 Å, 60 Å, 60 Å). The remaining cell volume is filled with DMF to mimic the synthetic protocol as closely as possible. This initial seeding is followed by a geometry optimisation and NVT (constant number of molecules, cell volume and temperature) MD step, using the HOOMD-blue defaults for the number of geometry optimisation and molecular dynamics cycles (one million and one hundred thousand, respectively), a van der Waals cutoff of 10 Å, an integration timestep of 0.0001, and a HOOMD-blue temperature factor during the MD of 1.2.^8,9^
2. The DMF is then temporarily removed from the cell, and, in the case of

***p*-phenyl-Py-CMP**, ***m*-phenyl-Py-CMP** and ***o*‑phenyl‑Py‑CMP**, *two* linkers are added (mimicking the experimental stoichiometry) to the system using Ambuild *growBlocks* steps (Figure S2 (a)). Here, each linker is bonded at random to an available carbon end group, replacing the bromine ‘cap atom’ that was previously in place in the unreacted building blocks. Pre-defined bonding rules state an unreacted end group within a pyrene or linker building block may bond either to another building block of the same type (*e.g.* a tetrabromopyrene bonding to another tetrabromopyrene), or to a block of the other type (*e.g.* a tetrabromopyrene bonding to a linker), to allow for structural diversity within the amorphous system. The bond length and bond angle margins here are 0.5 Å and 5° respectively.

1. The DMF is then re-filled, and an Ambuild *zipBlocks* step (Figure S2 (b)) performed, where the defined bond length and bond angle margins are increased to 5 Å and 70°, respectively, before examining whether the molecules present are close enough to form any additional bonds within the extended bond length and bond angle constraints specified; if so, these bonds are formed. This is immediately followed by a further optimisation and MD step.
2. Following this, in all cases the DMF is removed once again, and a single tetrabromopyrene building block is added to the system using an Ambuild *growBlocks* step, which is again followed by re-seeding of the DMF solvent, an Ambuild *zipBlocks* step using the same margins as above, and a geometry optimisation and MD step.

Steps (B) to (D) are repeated as necessary to produce clusters containing ten pyrene building blocks and, in the case of ***p*-phenyl-Py-CMP**, ***m*-phenyl-Py-CMP** and ***o*‑phenyl‑Py‑CMP**, twenty linker building blocks, matching the experimental stoichiometry. For each system, 100 clusters were grown independently.


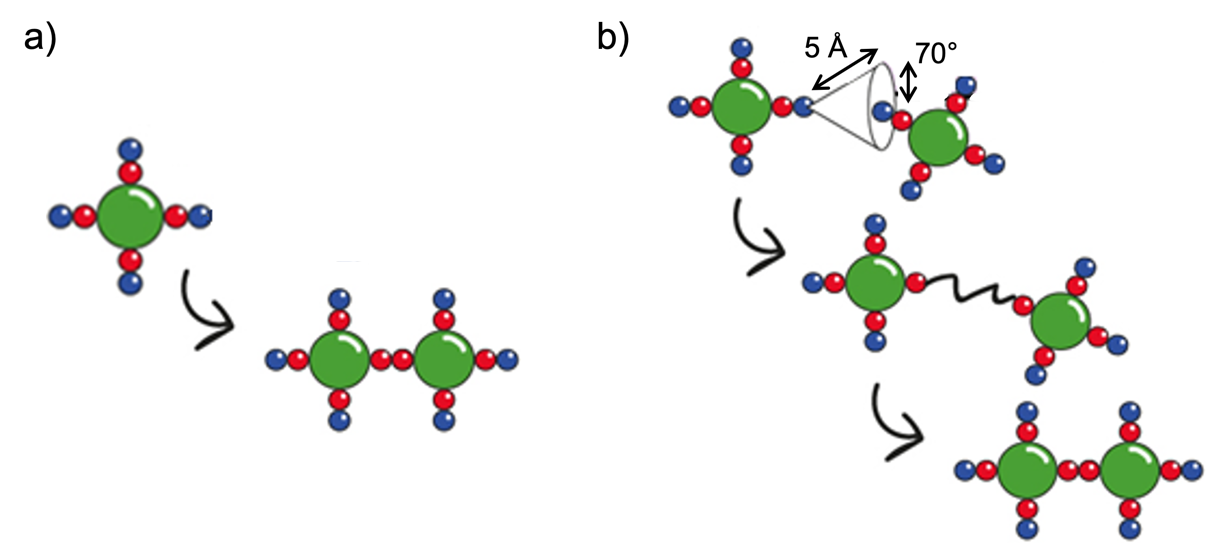


**Figure S2.** Cartoon representation of the Ambuild (a) growBlocks and (b) zipBlocks steps. Within the growBlocks step, a new building block is added directly onto an existing fragment within the cell providing there is a free end group that meets the desired bonding criteria. Within the zipBlocks step, two unreacted end groups within the cell may react if they (i) meet the desired bonding criteria and (ii) are within a cone, where the length of the cone is the extended zipBlocks bond length constraint and the radius of the widest part of the cone is derived from the extended zipBlocks bond angle constraint. In the case of the CMPs modelled here, the bond length constraint is 5 Å and the bond angle constraint is 70°.

**Figure S3.** The full Ambuild cluster generation process for one **Py-CMP** cluster. (a) Stepwise formation going from the original simulation cell to a two-pyrene system, (b) to (i) are intermediates in the cluster generation process, composed of two to nine pyrene building blocks, whereas (j) shows the final cluster composed of ten pyrene building blocks (after removal of the DMF solvent). *Key*: C – grey, H – white, Br – burgundy, N – blue, O – red.


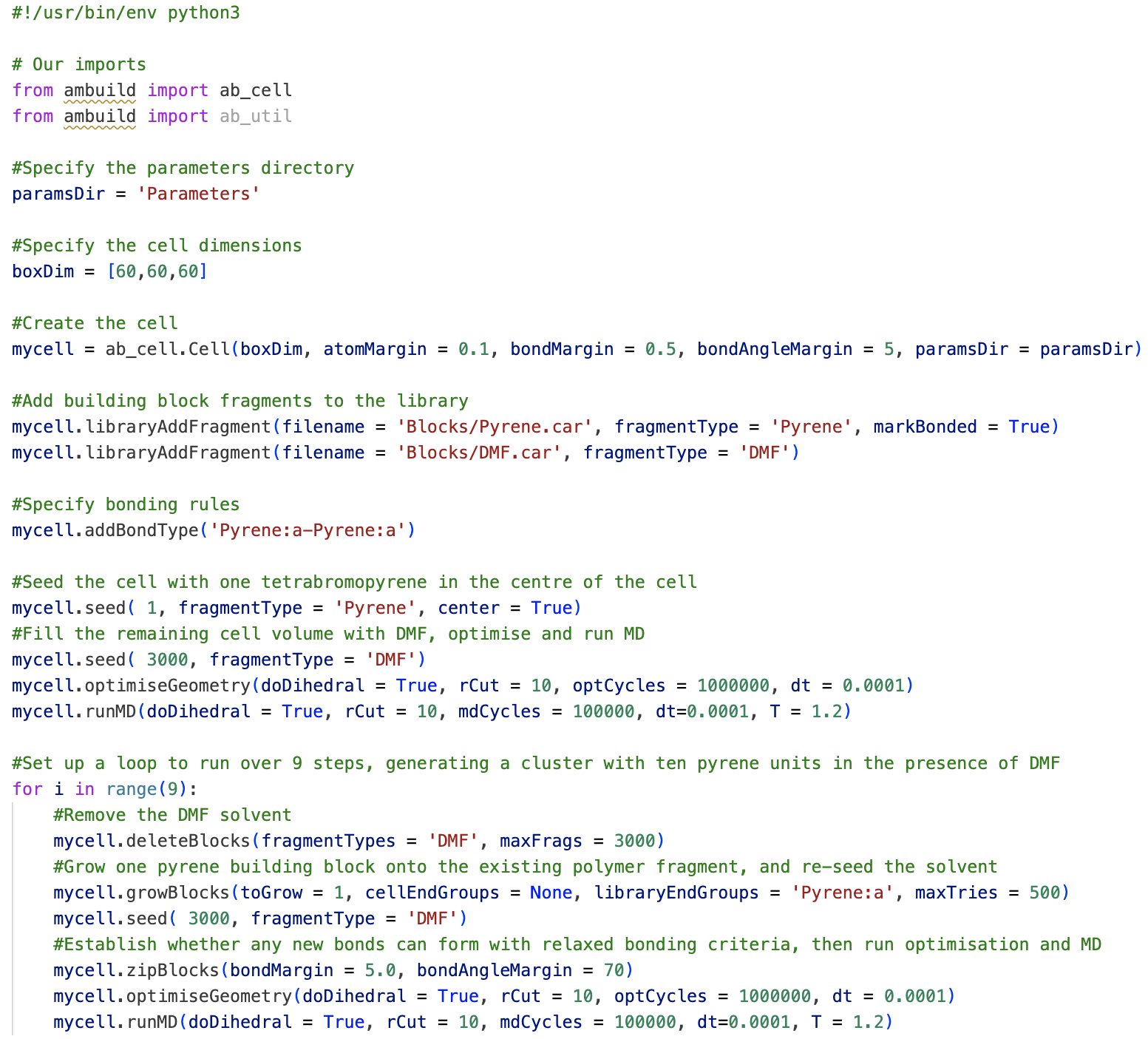


**Scheme S2.** An Ambuild input file for the full cluster generation of one **Py-CMP** cluster. This code can be found on the Ambuild Github site (<https://github.com/linucks/ambuild/wiki>) in the wiki section “11_Example_Input_Scripts”, Section 11.6 Example cluster generation for Py-CMP.

1. **Statistical analysis**

**Table S1.** The number of each structure type observed from the 100 individual Ambuild cluster generation runs for each material type (**Py-CMP**, ***p*-phenyl-Py-CMP**,

***m*-phenyl-Py-CMP** and ***o*‑phenyl‑Py‑CMP**). The values can thus be interpreted as the percentage of that structure type observed in each case. Examples of each structure type for each material are given in Figures S4–S7.

**Table S2.** The composition of each subset of structures studied by electronic structure calculations for each material, designed to reflect the full ratio of types found above. Examples of each structure type for each material are given in Figures S4–S7.

**Figure S4.** Example structures of each structure type used within the subset of **Py-CMP** structures analysed, with the structural MCR features highlighted in blue and singly/doubly branched pyrene building blocks in yellow. (a) Singly branched, (b) doubly branched,

(c) multiply branched, where branching occurs throughout the structure, (d) single ring,

(e) double ring. Structure files in the xyz format can be found in the supporting information labelled as Figure S4 a, b, c, d, and e.

**Figure S5.** Example structures of each structure type used within the subset of

***p*-phenyl-Py-CMP** structures analysed, with the structural MCR features highlighted in blue. (a) multiply branched, where branching occurs throughout the structure, (b) single ring. Structure files in the xyz format can be found in the supporting information labelled as Figure S5 a and b.

**Figure S6.** Example structures of each structure type used within the subset of

***m*-phenyl-Py-CMP** structures analysed, with the structural MCR features highlighted in blue. (a) multiply branched, where branching occurs throughout the structure, (b) single ring,

(c) double ring, (d) triple ring, (e) quadruple ring, (f) quintuple ring. Structure files in the xyz format can be found in the supporting information labelled as Figure S6 a, b, c, d, e, and f.

**Figure S7.** Example structures of each structure type used within the subset of ***o*‑phenyl‑Py‑CMP** structures analysed, with the structural MCR features highlighted in blue. (a) multiply branched, where branching occurs throughout the structure, (b) single ring,

(c) double ring, (d) triple ring, (e) quadruple ring, (f) quintuple ring, (g) sextuple ring. Structure files in the xyz format can be found in the supporting information labelled as Figure S7 a, b, c, d, e, f, and g.

1. **Computational characterisation**

**UV–Vis (absorption)**

There are several important factors that influence the comparison of calculated and experimental UV–vis data. These include the influence of the *ground state geometry of the structure* used, the use of the *vertical approximation*, the choice of *model chemistry*, any experimental artifacts that are not considered, and the finite size of the cluster models we base our predictions on.

As such, we take a pragmatic approach to the calibration of the UV–vis spectra. Based on our “best” calculated spectra, we determine an “optimal shift” relative to the experimental results recorded for **Py-CMP**, to align the absorption maxima. This gives a shift of −0.83 eV relative to experiment. As detailed below, we attribute a shift of < −0.1 eV to the choice of geometry, a shift of around −0.1 eV due to the choice of model chemistry, a shift of around 0.3 eV to the use of the vertical approximation, and a shift of around −0.4 to −0.6 eV to the finite size effects. Thus we can post-rationalise the size of this shift.

We then use our shift of −0.83 eV for the other systems considered. The pre-determination of such a shift, excluding experimental influence in the calibration, is clearly a complex undertaking and will be considered in detail in future work.

**The ground-state geometry**

The relative energies between ground- and excited-states can be highly sensitive to the geometry at which the energy differences are considered. For a molecule like Py, with complex photophysical behaviour, this is definitely the case. It is therefore particularly important that we evaluate the excitation energies at a meaningful ground state structure.

The geometries of the clusters of the **Py-CMP** systems considered here (as generated by Ambuild) are all post-processed using the GFN2-xTB approach of Grimme and co-workers. To assess the impact of using these geometries, rather than “conventional” DFT or electronic structure geometries, we consider the 20 lowest excited states of the Py monomer, and their dependence on the structure used.

For this comparison, we have calculated a high quality (C-centre frozen core) coupled-cluster with single and double excited configurations (CCSD) cc-pVTZ geometry of the Py monomer, and calculated excitation energies at this geometry. These excitation energies are compared to those evaluated at the GFN2-xTB geometry. We employ two model chemistries for this comparison; TDA-CAM-B3LYP/cc-pVTZ and TDA-CAM-B3LYP/def2SVP. In each case, the choice of basis set is found to be the dominant factor affecting the excitation energies.

For the cc-pVTZ excitation energies, the mean absolute change over the lowest 20 excitation energies between the CCSD and xTB geometry is 0.03 eV; the maximum change observed is 0.05 eV. For the def2SVP excitation energies, the mean absolute change and maximum change are, to the precision quoted, identical. This suggests the xTB geometry we obtain for molecular Py is of a high quality and will not significantly impact the results over the use of a “more expensive” geometry.

**Figure S8.** A comparison of the computationally-derived UV–vis spectra of pyrene using the TDA-CAM-B3LYP/SVP model chemistry, at the CCSD/cc-pVTZ and xTB geometries.

**The vertical approximation**

Experimentally, the UV–vis absorption data reflects adiabatic transitions between the ground- and excited-states of the molecules (i.e., the geometry of the molecule naturally relaxes upon excitation to the relevant state that has been excited into), and includes broadening due to concurrent changes between vibrational states as the molecule is excited between electronic states. Here, we use the entirely standard *vertical approximation* in evaluating the UV–vis spectra from electronic structure calculations. The alternative would be to explicitly calculate the excited state geometry of each state individually. Given that, for the clusters we consider, typically at least 30 excited states meaningfully contribute to the predicted spectra, this is prohibitive.

The use of the vertical approximation invariably leads to an overestimation of any individual excitation energy (compared to experiment), although the extent to which each individual state energy is overestimated depends on the amount of geometric reorganisation upon excitation.

To probe this overestimation in our systems, we again consider the behaviour of molecular Py, for which high-resolution experimental UV–vis absorption data is available. We then compare our calculated values to this data. To consider this effect as independently as possible of any other influences, we primarily compare the molecular Py experimental data against EOM-CCSD/cc-pVTZ and TDA-CAM-B3LYP/cc-pVTZ data, calculated at the ground state CCSD/cc-pVTZ geometry. (Again, the CCSD values are all calculated with the core orbitals on the C atoms uncorrelated). This is contrasted to TDA-CAM-B3LYP/cc-pVTZ data calculated at the respective geometries of the two lowest energy singlet excited states, S_1_ and S_2_, again as calculated using the TDA-CAM-B3LYP/cc-pVTZ model chemistry. Allowing the geometry to relax gives a shift of around 0.3 eV relative to the unrelaxed geometry.

Contrasting the total state energies (the sum of the ground-state energy at the excited state geometry, plus the appropriate excitation energy), compared to the unrelaxed vertical excitation energy, gives a smaller shift of around 0.1–0.2 eV. In either case, these values remain a significant overestimate of directly comparable experimentally-observed values.

We conclude that this is a relatively significant factor in the deviation from experiment; around 0.3 eV of any discrepancy can be attributed to our neglect of adiabatic / geometry relaxation effects.

**The influence of model chemistry**

The model chemistry we have chosen to study the clusters is a compromise between cost and accuracy. For systems of this size, the use of coupled cluster methods (which would be the ideal option) is prohibitively expensive, in terms of both computational resource and compute time. As such, we use the CAM-B3LYP DFT-based approach. CAM-B3LYP is known to provide realistic excitation energies, and in general correlates well with coupled-cluster values for a range of molecules. In particular, it is known the use of CAM-B3LYP coupled with the Tamm–Dancoff approximation (TDA), is able to correctly reproduce the state ordering in linear condensed acenes, and indeed in Py, where many other DFT methodologies fail.

The spectra produced by broadening the EOM-CCSD/cc-pVTZ, and TDA-CAM-B3LYP/cc-pVTZ data are shown in Figure S9. Overall, the agreement is excellent; the relative positioning of individual peaks is slightly affected, but the two spectra are qualitatively very similar.

**Figure S9.** A comparison of the computationally-derived UV–vis spectra obtained from EOM-CCSD and TDA-CAM-B3LYP excitation data, calculated using the cc-pVTZ basis set, at the CCSD/cc-pVTZ ground state geometry.

It is recognised however, that this model chemistry results on average to a (relatively modest) overestimation compared to CCSD. This is exacerbated by the necessity of using an efficient basis set, which itself will give rise to an overestimation compared to using a more extensive basis set. Here, we have chosen the def2-SVP basis set to study the clusters, hence we use the CAM-B3LYP/def2-SVP model chemistry coupled with the TDA, to study the excited states of the clusters.

To quantify the extent to which this choice of model chemistry results in an overestimation relative to other choices, we consider TDA-CAM-B3LYP/cc-pVTZ excitation energies of the Py monomer, computed at the CCSD/cc-pVTZ geometry, and contrast with those values calculated using our chosen CAM-B3LYP/def2-SVP TDA model chemistry. There is a relatively small change in values observed. Some sensitivity in the obtained oscillator strengths is observed, but importantly the relative intensities are not significantly affected.

We conclude that an error of around 0.1 eV is introduced through the use of the TDA-CAM-B3LYP/def2SVP model chemistry, versus the use of either EOM-CCSD or TDA-CAM-B3LYP, in a larger triple-zeta basis.

**NMR**

The calculated shielding constants obtained from Perdew–Burke–Ernzerhof (PBE)/pcS-1 calculations were converted into experimentally-comparable chemical shifts by means of taking a reference shielding (corresponding to a chemical shift of 0.0 ppm) of 180.0 ppm. We obtain this reference shielding by a calibration process involving multiple well-characterised hydrocarbon molecules with similar structural features to the **Py-CMP**s considered in this work: hexane, cyclohexane, benzene, adamantane, pyrene, TMS, 1,4-dimethylbenzene, 1,3‑dimethylbenzene, and 1,2-dimethylbenzene.

Here, the ^13^C shielding constants are evaluated using PBE/pcS-1 calculations at geometries obtained for each individual molecule using the same xTB approach as for the **Py-CMP** clusters. These shielding constants are then ‘paired’ with experimental chemical shifts for those same molecules; a simple linear fit of the data, extrapolated to the *x*-axis crossing point (as in Figure S10) gives a value of 180.0 ppm for the conversion. This value is fully consistent with the observed shift required to match with the solid-state NMR data obtained for the four CMP systems, resulting in calculated spectra that match well with experiment.

**Figure S10.** Plot of the experimental chemical shift against the calculated isotropic shielding for a range of reference molecules to establish the value used to shift the calculated shielding values. *Key*: hexane – green, cyclohexane – red, benzene – yellow, adamantane – blue, pyrene – purple, TMS – gold, 1,4-dimethylbenzene – orange, 1,3-dimethylbenzene – teal, 1,2-dimethylbenzene – pink, trendline – black dashed line. The *R*^2^ value is 1.0.

**Periodic NMR**

The structure of a periodic model composed of a 6-membered pyrene macrocyclic ring in the quartz topology was optimised and had its NMR spectra calculated using CASTEP version 22.11^11^ on the High-End Computing facility at Lancaster University.^12-17^ The Perdew–Burke–Ernzerhof (PBE)^18^ generalised gradient approximation (GGA) was used as exchange–correlation functional throughout the optimisation and NMR calculations. Ultrasoft pseudopotentials were used to describe the core–valence interactions.^19,20^ The gauge-included projector augmented wave (GIPAW) algorithm was used during the NMR calculations to construct the all-electron wave function in the presence of the magnetic field.^21,22^ A plane wave basis set cut-off energy of 50 Ry and Monkhorst–Pack grid of 1 × 1 × 1 (to specify the points in *k*-space where the Brillouin zone should be sampled)^23^ were employed throughout the geometry optimisation and NMR calculations. The isotropic chemical shieldings were then shifted relative to the tetramethylsilane (TMS) standard (TMS shielding value = 189.72 ppm), where:

Chemical shift (ppm) = 189.72 ppm – isotropic chemical shielding (ppm)

**
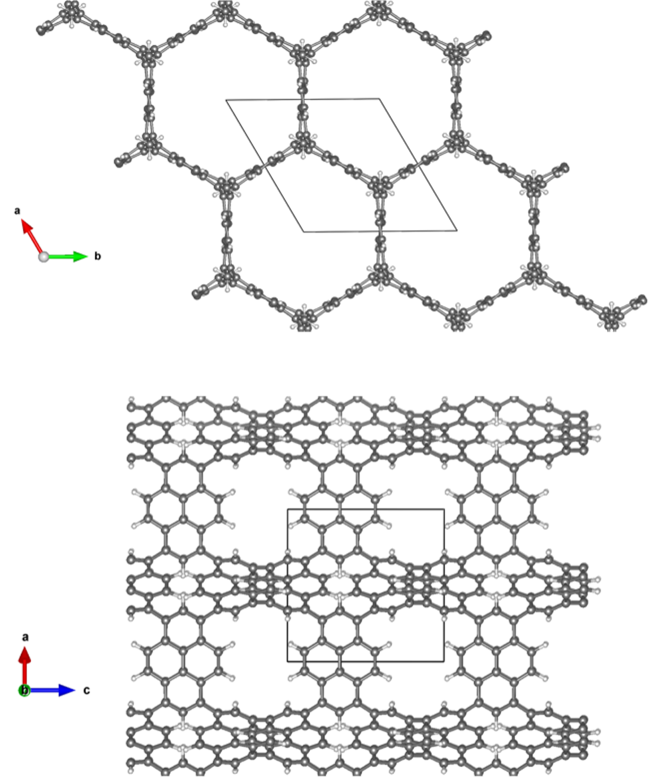
**

**Figure S11.** Structure (left) and ^13^C NMR spectrum (right) of the periodic pyrene structure calculated using CASTEP.

1. **Spectral broadening**

Spectral broadening involves applying a shape function, whose height is determined by the calculated intensity, with a full-width at half-maximum (FWHM) defined empirically, for each observed signal. This helps to at least empirically portray lifetime effects, accentuate any near-degeneracies where individual peaks may not be readily distinguishable, and represent the inherent uncertainty in the peak positions introduced by the finite size of the representative clusters, and the accuracy of the underlying model chemistries.

For the IR spectra, following Grimme and co-workers,^24^ an empirically derived uniform wavenumber scaling factor of 0.990 was used to calibrate all calculated wavenumbers. No scaling was applied to the individual IR intensities calculated. A Lorentzian-based broadening scheme, with a FWHM of 5 cm^−1^, was employed throughout.

The ^13^C NMR shielding constants calculated for each system were similarly broadened using a Lorentzian, with a FWHM of 1 ppm. Here, the shielding constants were converted to chemical shifts using a value of 180 ppm as the reference shielding.

UV–vis spectra were obtained by applying a Voigt function (an interpolation of a Gaussian and Lorentzian function) around each peak, with parameters 𝜎 = 0.0025 and γ = 0.04.

A schematic figure illustrating this process is shown below in Figure S12. It shows the raw data of the discrete energies and intensities, as a comb plot; the process of broadening each of these discrete points; and the combination of these to produce a final computational spectrum. Note that in the schematic, as in the ‘real’ data, we normalise the data such that the highest intensity peak has a height of 1.0.

**Figure S12.** An illustration of the broadening process used throughout to turn the raw calculated discrete ‘stick’ data into continuous spectral data to facilitate comparison with experiment.

1. **Pyrene linear chain**

Three linear chain models were constructed: First where the Py are joined at the 1-, and 6- position, this configuration affords the least steric hinderance; second where the Py are joined at the 1-, and 8- positions; and thirdly where the Py are joined in a random combination of 1-, 3-, 6- , and 8- positions. The spectra are shown in Figure S13-A.

To examine how the properties of a Py chain evolve with chain length, and to validate that we achieve adequate convergence of spectra to that of the “infinite” experimental oligomer, we generated a series of random Py chains of increasing length, culminating in a randomly-bonded (“**R**”) 10Py chain model **10Py(R)**.

Due to the randomness of potential connectivity here, we generated a series of ten such **10Py(R)** chains, grown sequentially, where each pyrene unit added has an equal chance of 1,3-, 1,8- or 1,6-bonding, resulting in randomly-bonded chains of between 2 and 10 Py units in length. From these topologies, we then calculated XTB-relaxed structures (and XTB-derived IR spectra), and calculated NMR shieldings and TDA excitation energies and oscillator strengths as in Sections S4 and S5. This data was then used to produce average calculated spectra in the same manner outlined in section S5.

We find that for each of the predicted IR, NMR and UV–vis, the spectra demonstrate reasonable convergence by 10 units (see Figure S.13-B); the change associated with adding an additional Py unit (averaged over the ten different **10Py(R)** models) rapidly decreases as the chains approach 10 units in length.

In Figure S.13-C(a) we illustrate how the maximum-intensity predicted NMR peak shifts with increasing chain length; this shows convergence past ~4 units. To further check for convergence of the UV–vis spectrum, we similarly plotted the absorption on-set against the number of units in Figure S.13-C(b), as previously performed by Zwijnenburg for 1,3-bonded pyrene chains,^25^ and find good convergence is reached by 10 units.

To further probe the implications of the choice of oligomer size on the predicted properties of **Py-CMP**, we generated a set of ten 15-unit **15Py(R)**, by growing 5 more Py units onto each **10Py(R)** chain. As expected, in Figure S.13-D we find minimal differences between the predicted average IR and NMR of the 10- and 15-unit chains, and a slight shift of the calculated UV–vis absorption on-set for **15Py(R)** to lower energy.

Here, we attempt to quantify the change in spectra for each chain length from 1–10 units and to the 15-unit chain, using Salvador and Chan’s FastDTW ^26, 27^ time-warping algorithm, as a method of ‘measuring’ the difference between each predicted spectrum as a “distance”, where “distance” is the distance of the warp path and is related to both x and y axis warping paths.^26, 27^ Figures S.13-E(a)—(c) demonstrate the convergence of this ‘distance’ from the properties of **15Py(R)**, to within the expected “distance” between models of the same N (i.e. the effect of truncation on the “distance” value is lower than that that arises due to structural diversity effects), can be seen by 10 units in each case, indicating that 10 units is a reasonable length for our oligomer models as the properties change increasingly less with each additional unit.

Importantly, the effects due to chain truncation as observed here are significantly smaller than those due to the diversity of structure type found in our later analyses. Our calibration procedures for generating the experimentally comparable spectra therefore seem to adequately account for the remaining effects of chain truncation in this case (particularly where the variation with chain length is most significant, in the case of the UV–vis spectra).

As the 15-unit models require significantly more time and computational resources to predict their spectra, and because the properties of our 10-unit cluster models allow for sufficient structural diversity to provide a meaningful match to the experimental **Py-CMP** spectra, we believe the 10-unit models are an appropriate system size compromising between reasonable accuracy and computational tractability.

**
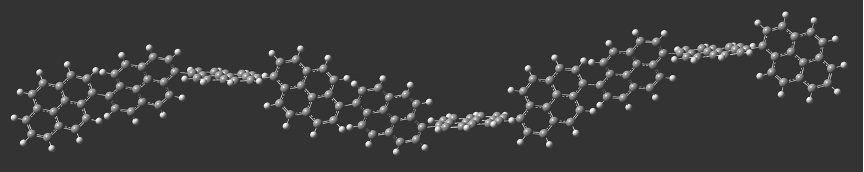
**

**10Py(1,6)**

**
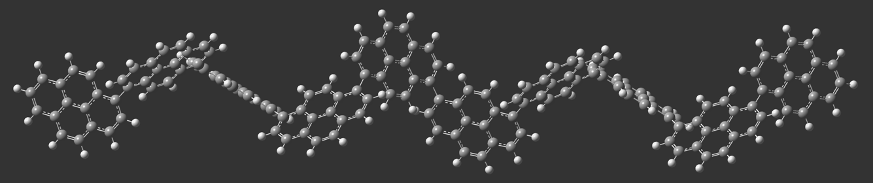
**

**10Py(1,8)**

**
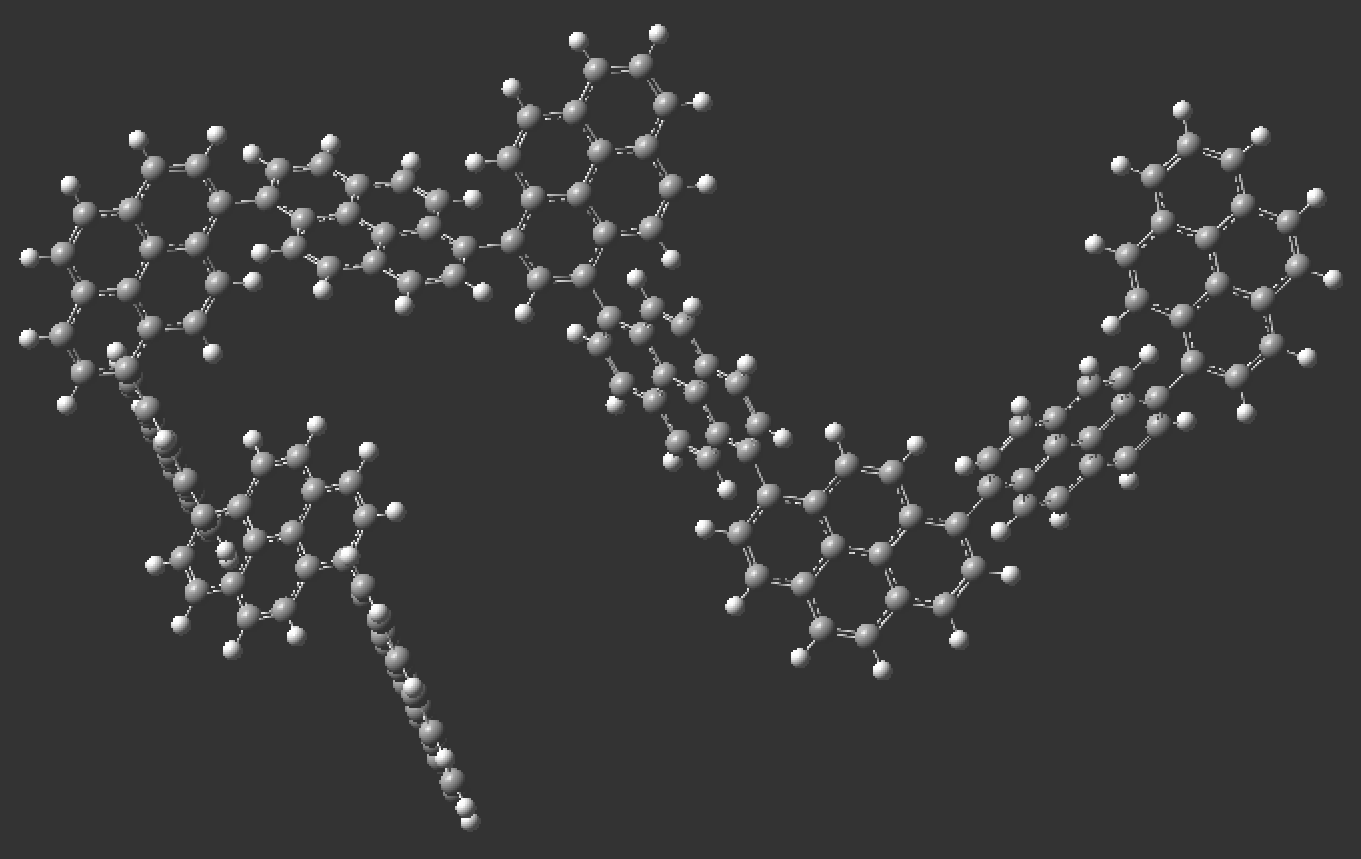
**

**Example 10Py(R)**


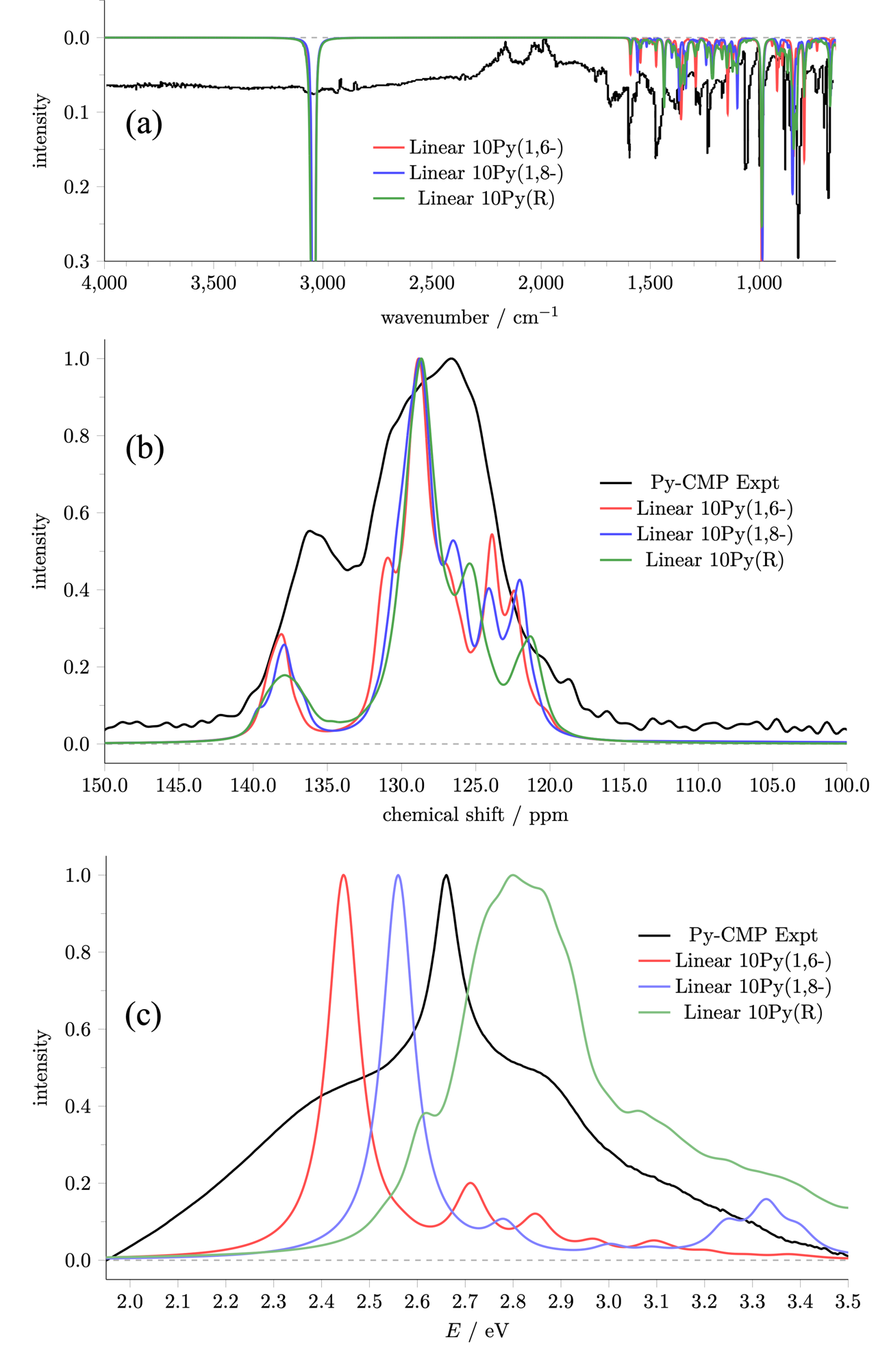


**Figure S13-A.** for **Py-CMP**, a comparison of the experimentally determined data (in black) compared to the computationally-derived spectra for the “linear” (non-branched) **10Py** models where the pyrenes are joined at the 1-, and 6- position (red), where the pyrenes are joined at the 1-, and 8- position (blue), and a random combination of 1-, 3-, 6-, and 8- positions (green). As in the other such figures, (a) shows the IR spectra, (b) the NMR, and (c) the UV–vis. Note the UV–vis data demonstrates considerably more structure-dependence than the IR and NMR data in this case.

**
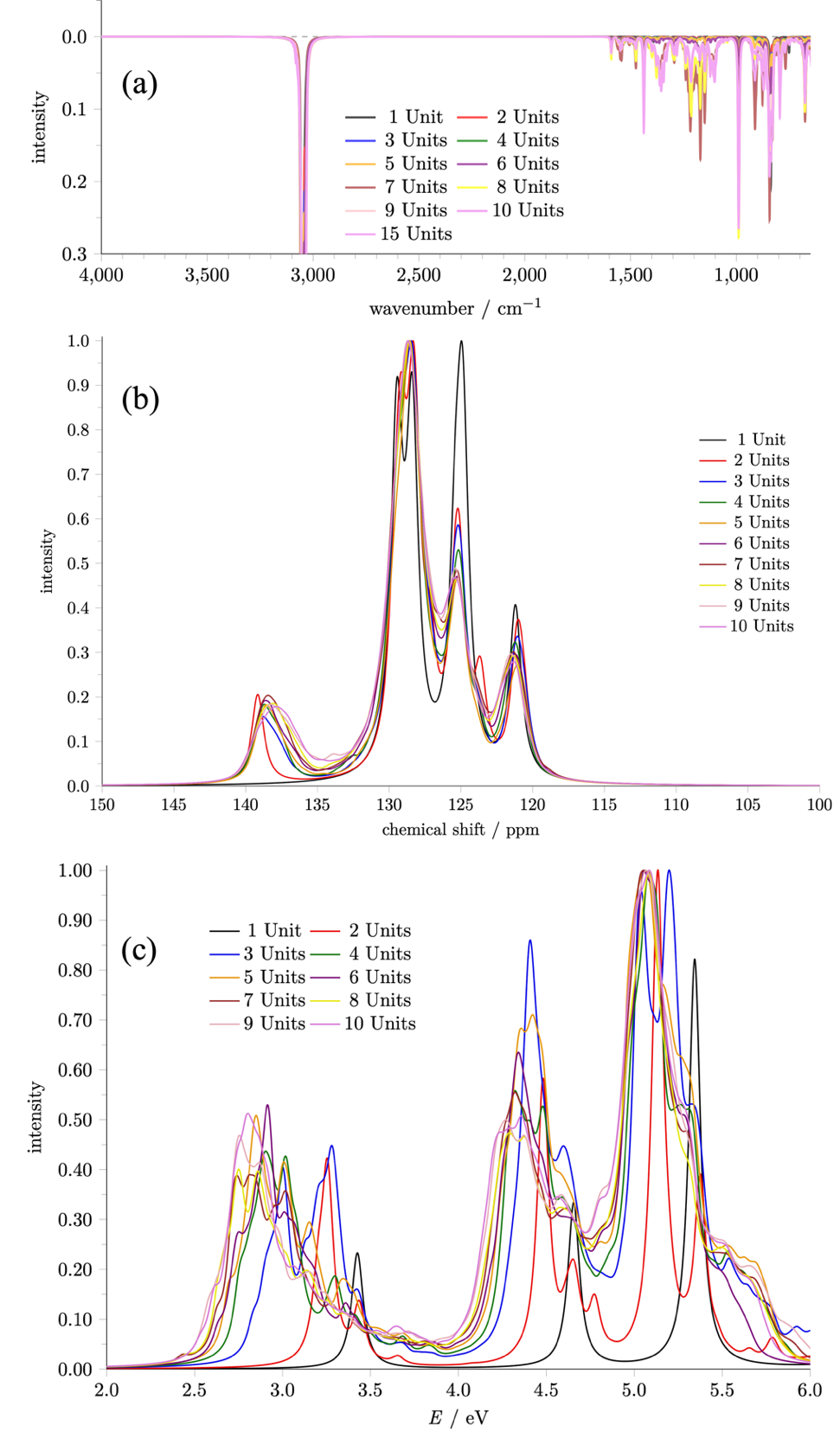
**

(c)

(d)

**Figure S13-B.** For **Py-CMP,** the spectra of the **10Py(R)** linear model where pyrenes are joined in a random combination of 1-, 3-, 6- , and 8- positions showing a comparison between the combined computationally-derived spectra for a set of ten “linear” models as they increase in length by 1 pyrene unit up to **10Py(R)**. (a) shows the IR spectra, (b) the NMR, and (c) the UV–vis. Again, the UV–vis data demonstrates considerably more structure-dependence than the IR and NMR data. The minor peak at ~135–140 ppm in the predicted NMR increases in intensity and broadens as the number of units increases, as this signal arises due to the different Py–Py bonded carbon environments. The UV–vis spectrum is extended here to capture peaks at higher eV values than experimentally measured. It possesses three major peaks, which shift to lower energy with increasing chain length, consistent with an increase in conjugation length. The lowest-energy absorption peak, which is captured in the experimental spectrum, shifts by the greatest proportion. Overall, good convergence of spectra is observed for the predicted IR, NMR and UV–vis as the chain length approaches 10 units.


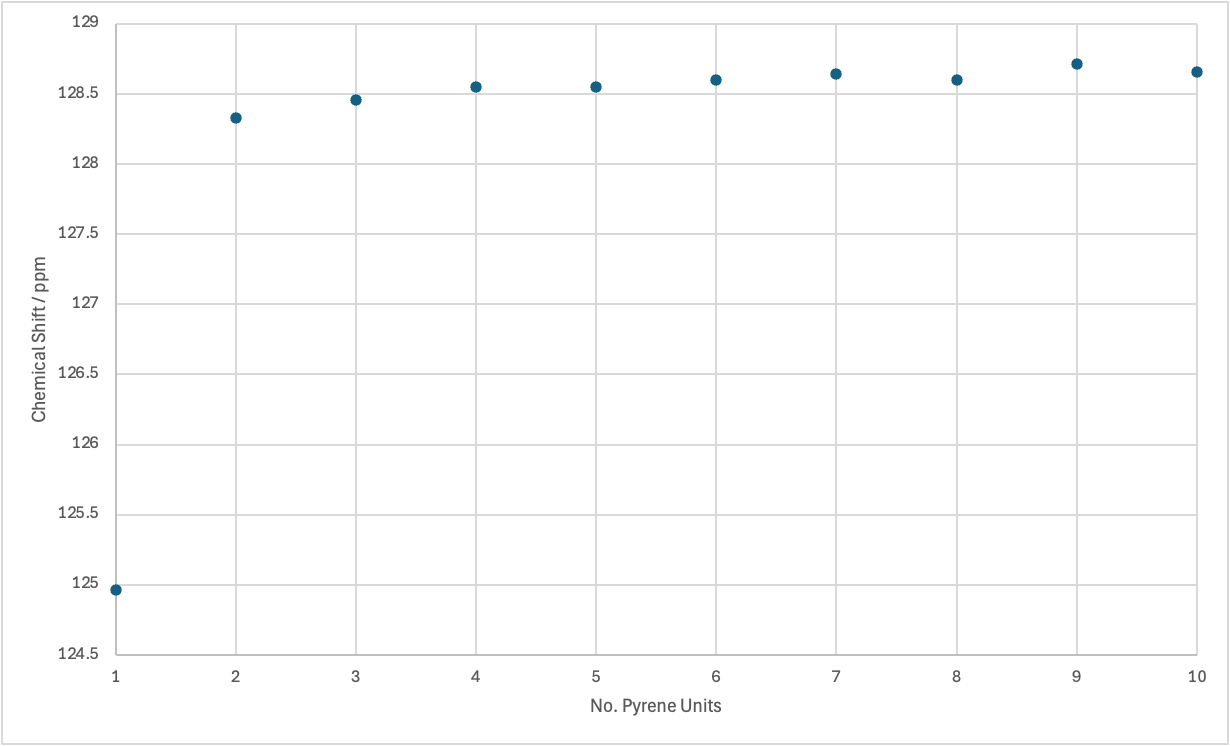


(a)

(b)


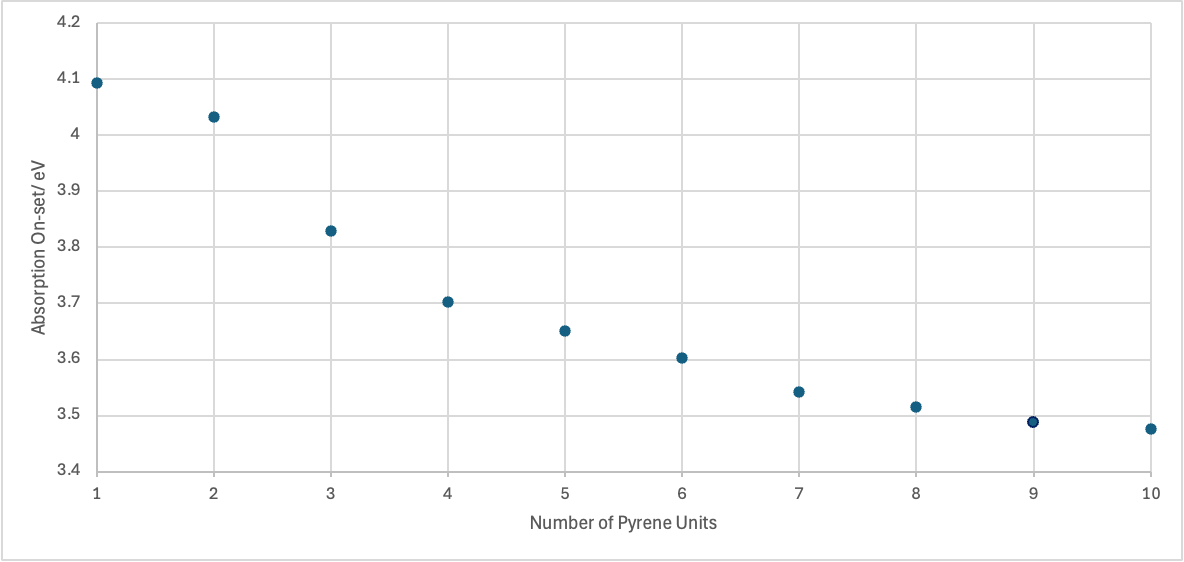


**Figure S13-C.** (a) a plot of the maximum intensity peak position for the combined NMR spectra of the **R** chains against the number of pyrene units demonstrates good convergence from ~7 units. (b) a plot of the average absorption on-set of the ten **R** chains against the number of pyrene units demonstrates good convergence by 10 units.


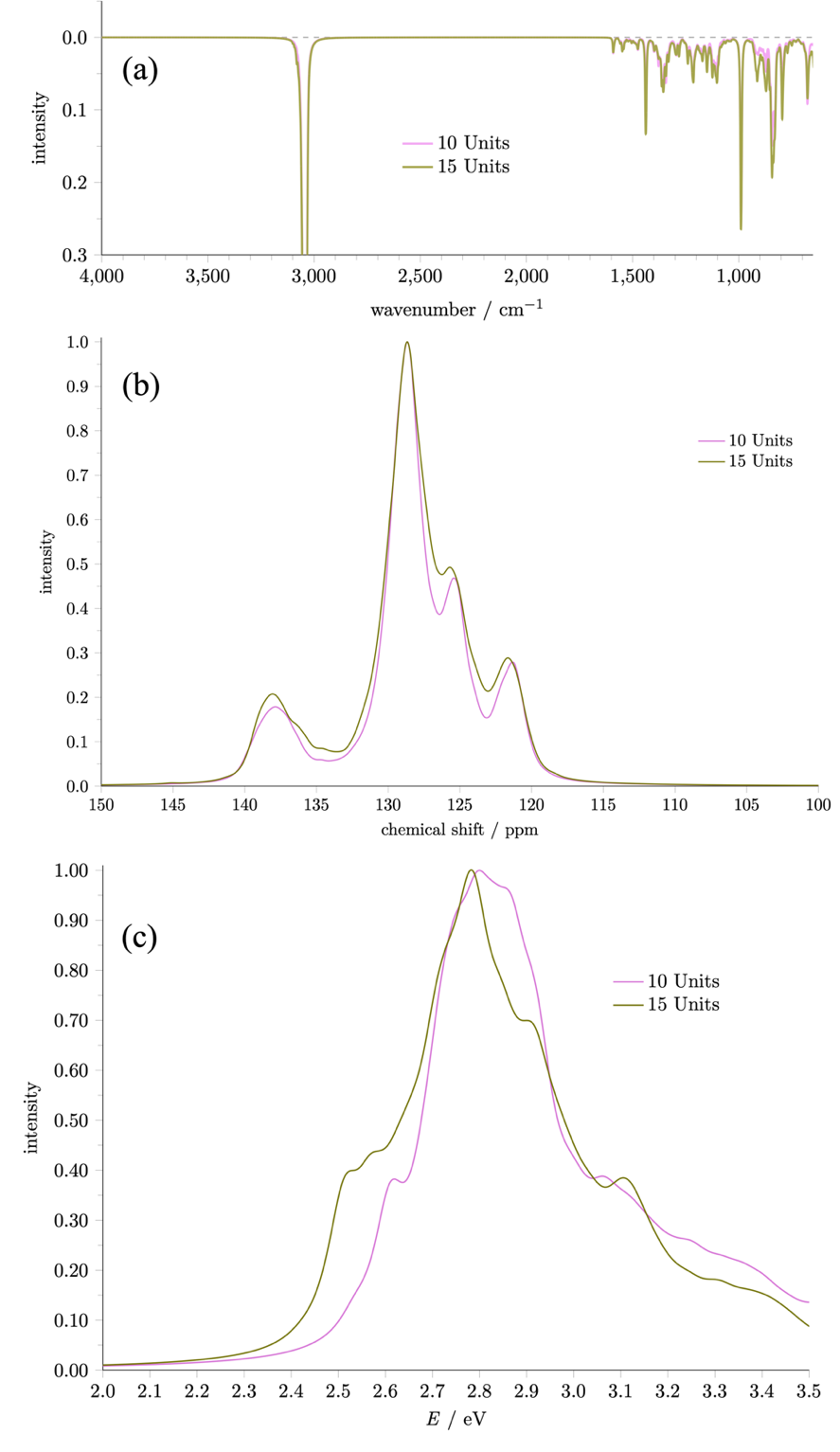


**Figure S13-D.** For **Py-CMP**, a comparison between the average predicted IR, NMR and UV-vis spectra for the **10Py(R)** and **15Py(R)** chains. As in the other such figures, (a) shows the IR spectra, (b) the NMR, and (c) the UV–vis. As expected, the greatest difference is observed in the UV–vis spectra, however the general shape of the signal remains the same between 10 and 15 units, with only a small shift in absorption on-set to lower energy for **15Py(R)**. Importantly, these effects are significantly smaller than those observed due to the diversity of structure type observed in subsequent analysis.


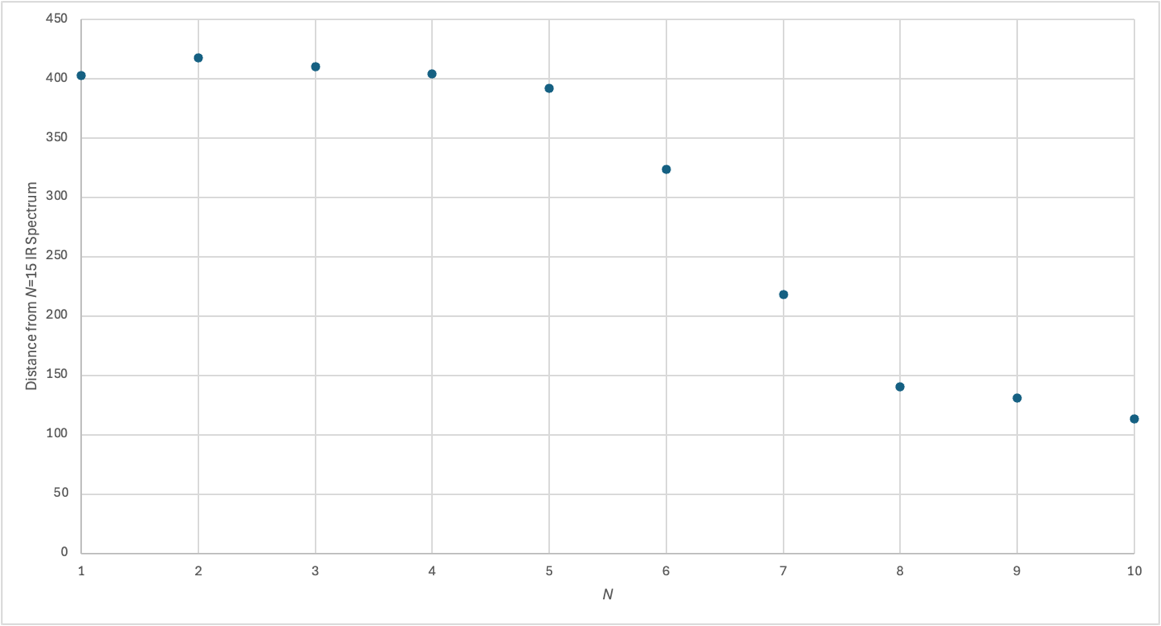

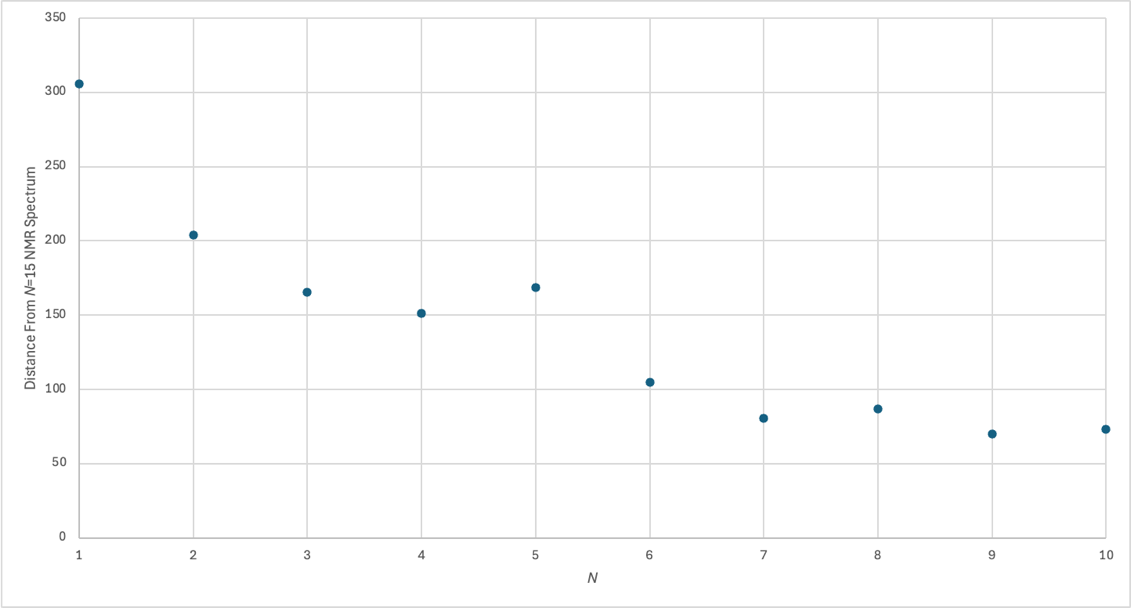

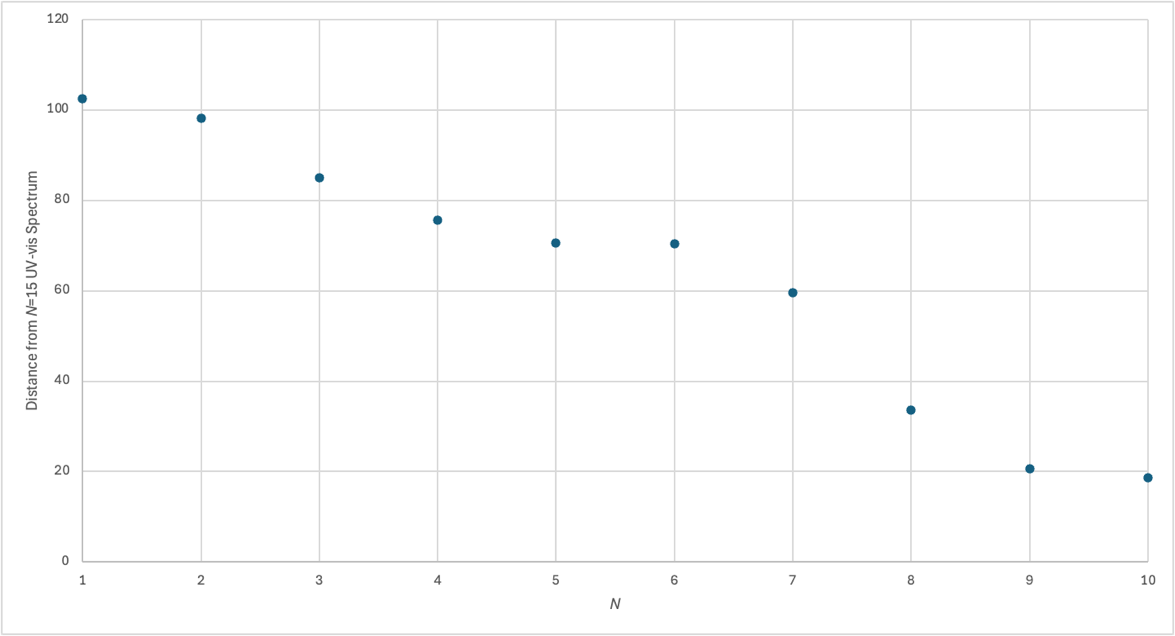


(a)

(b)

(c)

**Figure S13-E.** Plots of the calculated “distance” between the predicted spectra and **R** chain of length *N* respectively, where “distance” is the distance of the warp path and is related to both x and y axis warping paths.^26,27^ (a) average IR spectra compared to the average **15Py(R)** spectrum (b) average NMR spectra compared to the average **15Py(R)** spectrum. Convergence of distance can be seen by 10 units. (c) average UV-vis spectra from 2—3.5 eV compared to the average **15Py(R)** spectrum. Convergence of distance (to within the natural diversity expected between models of the same N) can be seen by 10 units in each case.

1. **Experimental spectra**

The experimental spectra as presented in Figure 4 of the main manuscript for all systems considered have been recorded on the samples synthesised for this work. Key spectral features are consistent with YPy and YDBPy from reference 2.

(d)

(e)

(f)

**IR**

The experimental IR spectra, as shown in Figure 4 (a), are raw spectra, presented without any data manipulation.

**NMR**

The experimental NMR spectra are shown in Figure 4 (b). Here, all spectral data has been shifted such that the minimum recorded intensity is zero for each individual system. The maximum intensity for **Py-CMP** has been normalised to a value of 1.0. All other systems have been calibrated relative to that maximum intensity value, to facilitate comparison between the spectra of the four systems.

**UV–vis (absorption)**

The experimental UV–vis absorption spectra are shown in Figure 4 (c). In this case, the raw spectral data has been baseline-corrected using a simple linear model, to ensure that features arising from the cuvette (below ~1.8 eV and above ~3.8 eV, which should be identical) are aligned across the four systems and to ensure the minimum recorded intensity in each case is zero. The correction has a relatively minor effect, but aids in highlighting the subtle differences observed between the four systems considered. As with the NMR case, the **Py-CMP** spectrum is normalised such that its maximum intensity is equal to 1.0; the remaining spectra are calibrated relative to that maximum intensity value.


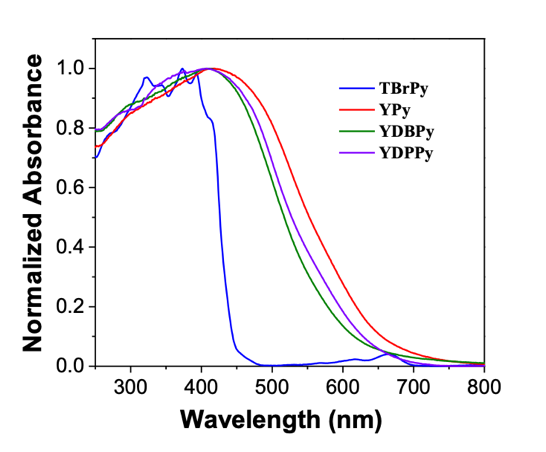

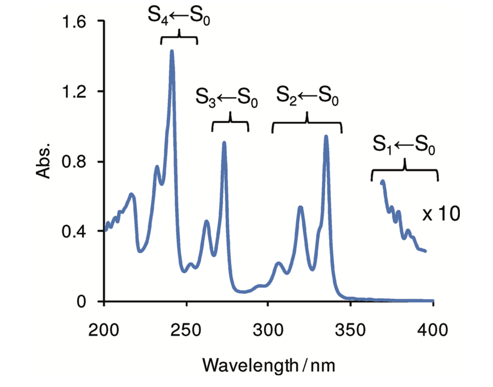


The experimental spectra of TBrPy (monomer), YPy (**Py-CMP**), and YDBPy (***p*-phenyl-Py-CMP)** replicated from reference 2 (left) and the experimental spectrum of Py (monomer), replicated from reference 25 (right).^2,28^ Our calculations do not reproduce the low energy (~650 nm) small absorbance in the TBrPy monomer; this is due to the T_1_ $\leftarrow$ S_0_ transition, which is formally forbidden without accounting for relativistic (spin–orbit coupling) effects.

The polymerisation process considerably affects the high-absorbance Py S_2_ $\leftarrow$ S_0_ transition, which becomes dominant at low energies, as this state is most sensitive to substitution at the 1-, 3-, 6-, and 8- positions. Indeed, this high oscillator strength state is the lowest energy state observed in TBrPy.

Above shows a comparison of the TBPy and Py monomer absorbance, calculated using TDA-CAM-B3LYP/SVP excitation energies and oscillator strengths (a) compared with our Py-CMP experimental absorption spectrum, on the same scale as all our other comparison data.(b) the same calculated data, on a larger energy scale, to show that the majority of the features lie to higher energies. (c) the equivalent data on a nm scale, for comparison with the monomer experimental data above.

1. **Spectral weighting**

Below in Figure S14 is a schematic representation of the process of ‘averaging’ two broadened spectra. In this schematic, we take a standard mean as the average, but we apply a weighted mean as the averaging process in other cases, in order that we can flexibly adjust the contribution of each individual structure or cluster type to the final spectrum obtained.

This process illustrates that peaks from different clusters at similar energies can ‘merge’ in the final spectrum, to yield relatively subtle features in the final spectrum.

In SI section 10, we show the individual spectrum predicted for each cluster, of each CMP considered. This allows the different features that give rise to the final calculated spectra of each CMP to be rationalised.

We also include figures of weighted averages of the **Py-CMP** and ***x*-phenyl-Py-CMP** data.

**Figure S14.** A schematic illustrating the process of obtaining ‘averaged’ overall spectra, weighting the contributions from each individual cluster type.

1. **Degree of phase separation**

(a) idealised co-polymer


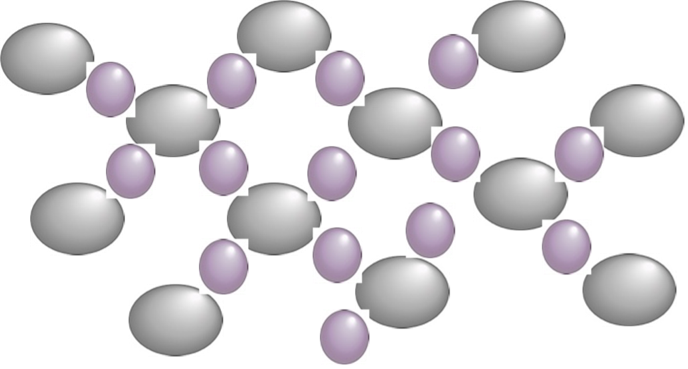


(b) fully integrated statistical co-polymer


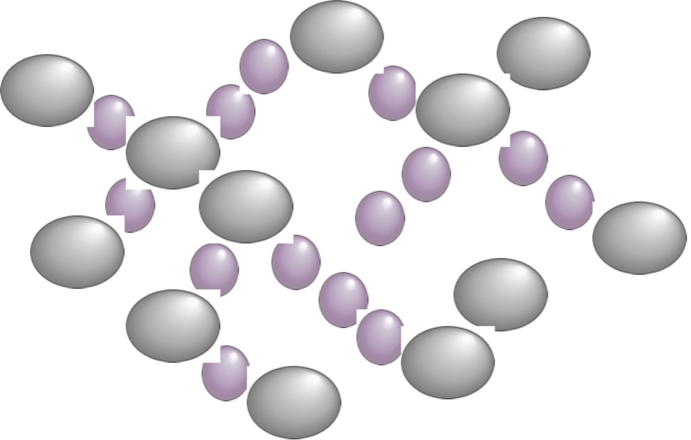


(c) block co-polymer


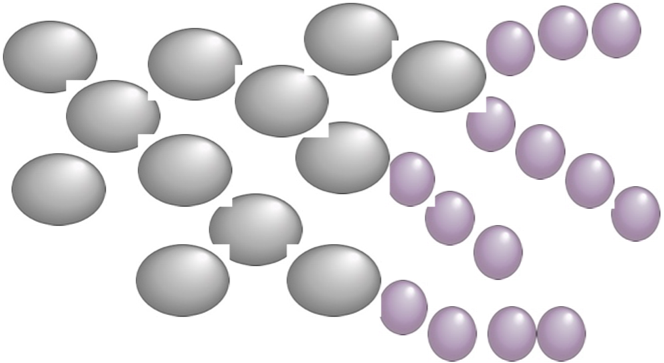


**Figure S15.** Cartoon representations of (a) an idealised co-polymer, (b) a fully integrated statistical co-polymer, and (c) block co-polymer.

**Figure S16.** Example structures of each structure type used within the subset of

***p*-phenyl-Py-CMP** structures analysed, with the pyrene building blocks given in black and the ***p*-phenyl** building blocks given in purple. (a) multiply branched, (b) single ring.

**Figure S17.** Example structures of each structure type used within the subset of

***m*-phenyl-Py-CMP** structures analysed, with the pyrene building blocks given in black and the ***m*-phenyl** building blocks given in green. (a) multiply branched, (b) single ring, (c) double ring, (d) triple ring, (e) quadruple ring, (f) quintuple ring.

**Figure S18.** Example structures of each structure type used within the subset of ***o*‑phenyl‑Py‑CMP** structures analysed, with the pyrene building blocks given in black and the ***o*-phenyl** building blocks given in red. (a) multiply branched, (b) single ring, (c) double ring, (d) triple ring, (e) quadruple ring, (f) quintuple ring, (g) sextuple ring.

1. **Cluster spectra compared to experiment**


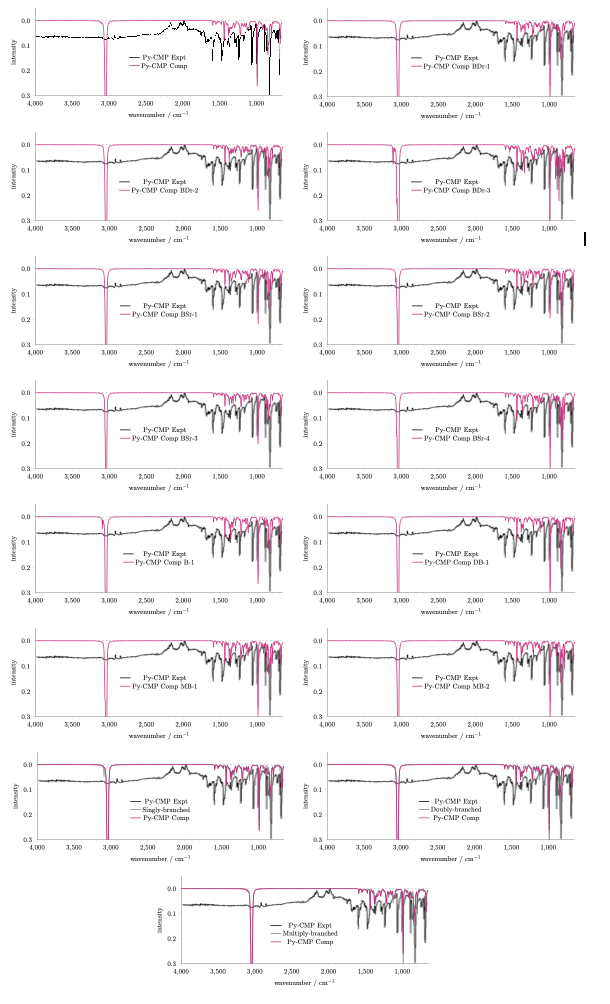


**Py-CMP**


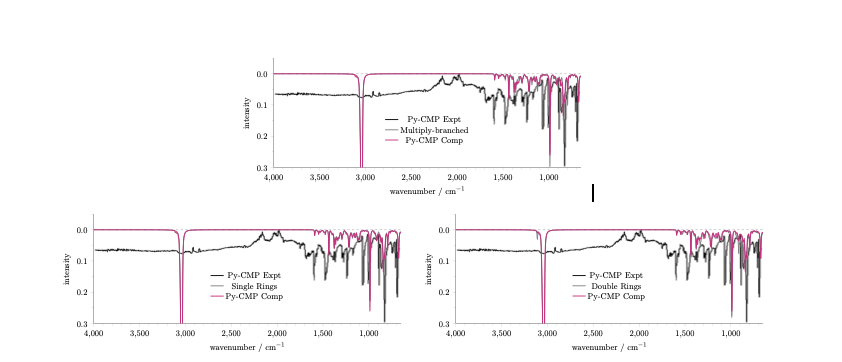


**Figure S19.** IR spectra of experimental (black) and computational (pink) **Py-CMP**.

*Key*: Expt – experimental, Comp – computational, considering the whole subset,

Comp BDr-1 – double ring structure X, Comp BSr-X – single ring structure X,

Comp B-1 – singly branched structure 1, Comp DB-1 – doubly branched structure 1,

Comp MB-X – multiply branched structure X. The final plots show the averaged spectra for each of the different types, comparing with experiment and the whole subset.

We note that the 1075 cm^−1^ in the IR spectrum of **Py**‑**CMP** (and several other less prominent features to lower wavenumber) are still absent from the computed IR spectra; this is independent of the cluster model used for the spectrum. These differences can be attributed to the absence of Br in our cluster models. Experimentally, these materials are characterised to contain roughly 4 wt% Br; this equates to 1 Br atom per 10-Py cluster.

An IR spectrum, calculated for tetrabromopyrene (shown below) demonstrates that both the key 1075 cm^−1^ peak, and many of those less prominent features in the fingerprint region, are well-accounted for via the inclusion of Br in the structure. Similarly, anharmonic effects (which cannot realistically be accounted for in cluster models of any appreciable size) are also found to particularly influence agreement in this region of the spectrum; we find that for the Py monomer, an harmonic calculation finds 13 fundamental vibrational modes that notably contribute to the spectrum below 1000 cm^−1^, whereas an anharmonic calculation finds an additional 25 combination bands that contribute to the spectrum.

**Figure S19A.** A comparison of the B3LYP/cc-pVDZ harmonic IR spectrum, calculated on molecular Py, and tetrabromopyrene (TBrPy). The region below 1200 cm^−1^ is particularly affected by the substitution of H for Br (a wavenumber scaling of 0.97 is applied here, consistent with the model chemistry).


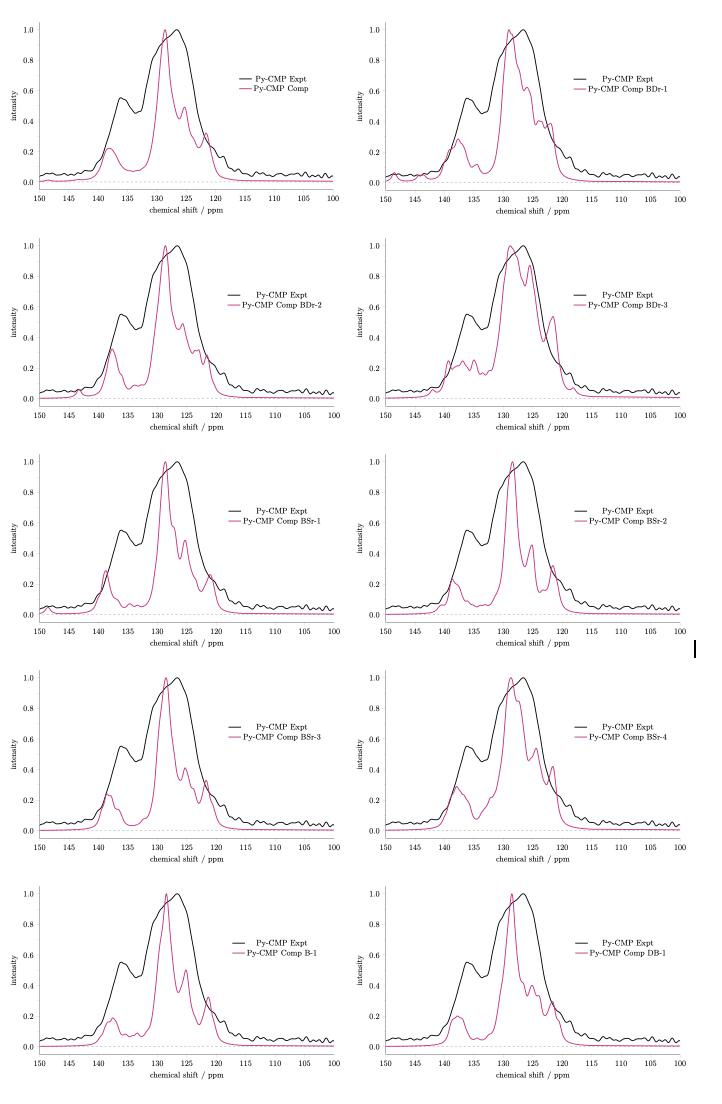


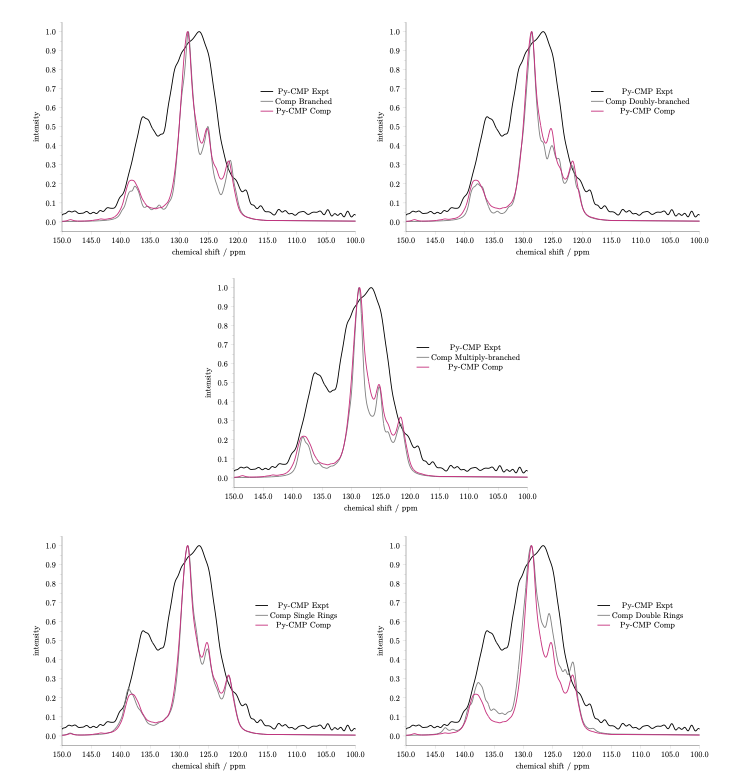


**Figure S20.** NMR spectra of experimental (black) and computational (pink) **Py-CMP**.

*Key*: Expt – experimental, Comp – computational, considering the whole subset,

Comp BDr-1 – double ring structure X, Comp BSr-X – single ring structure X,

Comp B-1 – singly branched structure 1, Comp DB-1 – doubly branched structure 1,

Comp MB-X – multiply branched structure X. The final plots show the averaged spectra for each of the different types, comparing with experiment and the whole subset.


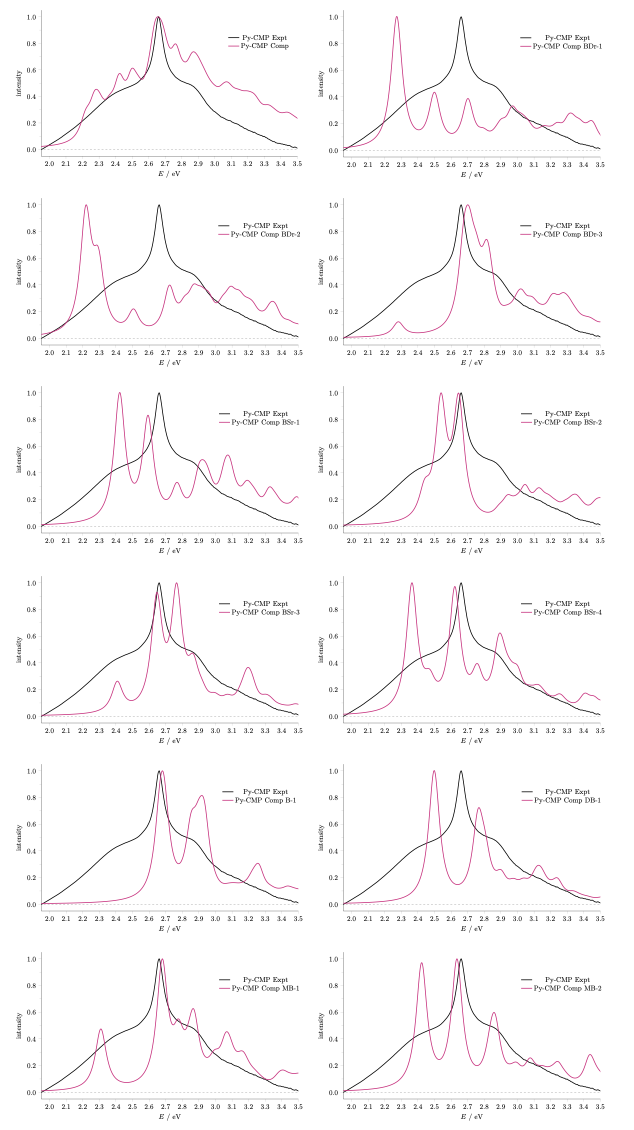


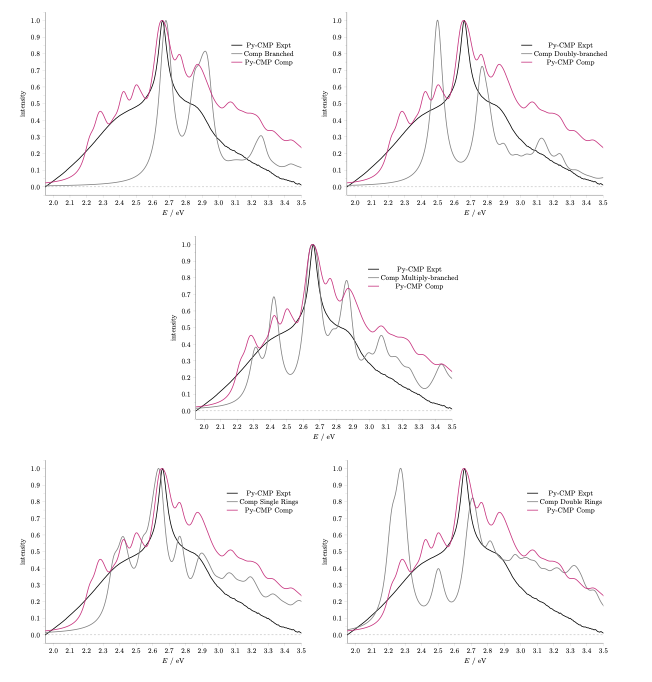


**Figure S21.** UV–vis spectra of experimental (black) and computational (pink) **Py-CMP**.

*Key*: Expt – experimental, Comp – computational, considering the whole subset,

Comp BDr-X – double ring structure X, Comp BSr-X – single ring structure X,

Comp B-1 – singly branched structure 1, Comp DB-1 – doubly branched structure 1,

Comp MB-X – multiply branched structure X. The final plots show the averaged spectra for each of the different types, comparing with experiment and the whole subset.

***p*-phenyl-Py-CMP**


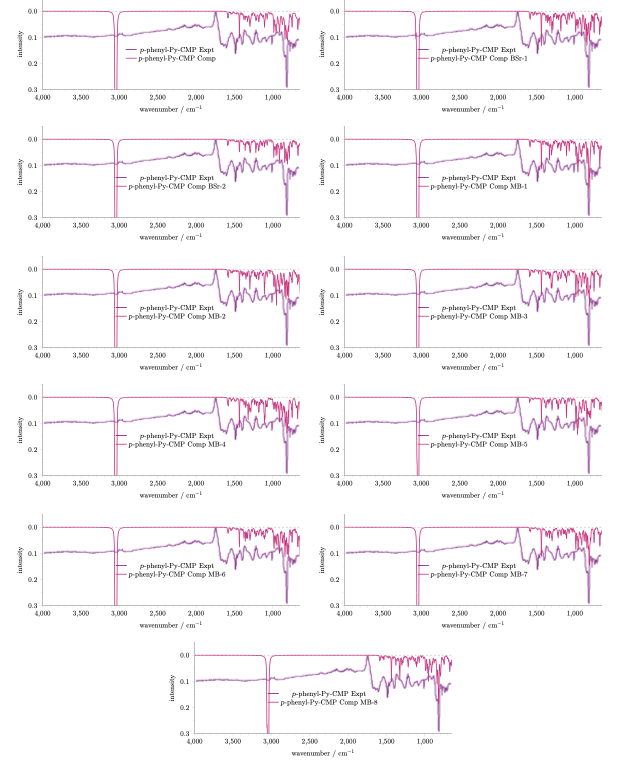


**Figure S22.** IR spectra of experimental (purple) and computational (pink)

***p*-phenyl-Py-CMP**. *Key*: Expt – experimental, Comp – computational, considering the whole subset, Comp BSr-X – single ring structure X, Comp MB-X – multiply branched structure X.

**
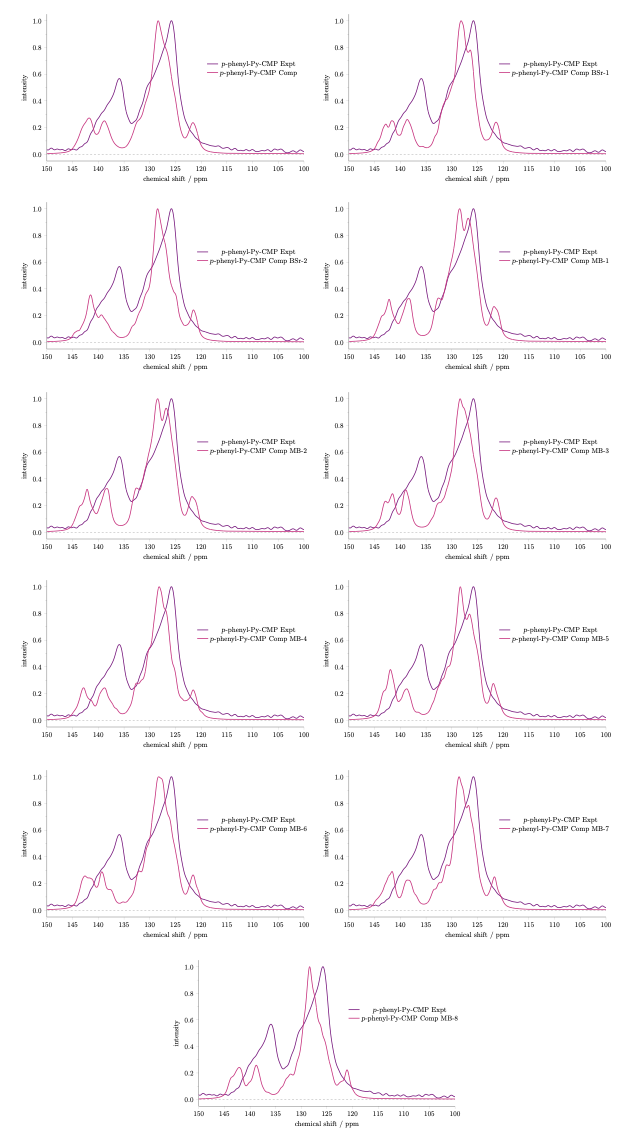
**

**Figure S23.** NMR spectra of experimental (purple) and computational (pink)

***p*-phenyl-Py-CMP**. *Key*: Expt – experimental, Comp – computational, considering the whole subset, Comp BSr-X – single ring structure X, Comp MB-X – multiply branched structure X.

**
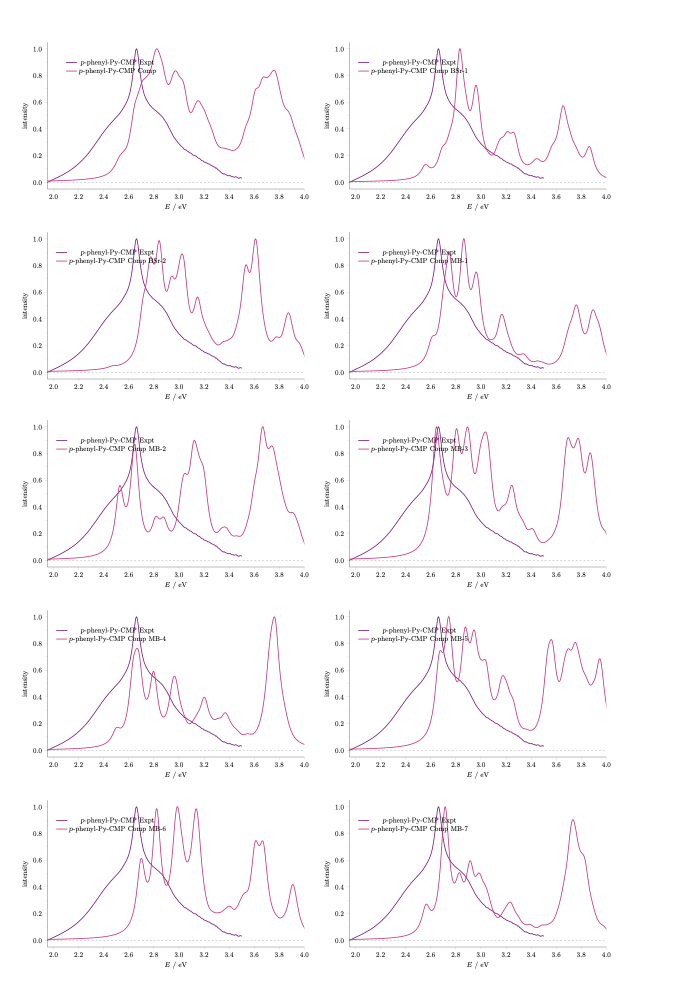
**

**Figure S24.** UV–vis spectra of experimental (purple) and computational (pink)

***p*-phenyl-Py-CMP**. *Key*: Expt – experimental, Comp – computational, considering the whole subset, Comp BSr-X – single ring structure X, Comp MB-X – multiply branched structure X.

***m*-phenyl-Py-CMP**


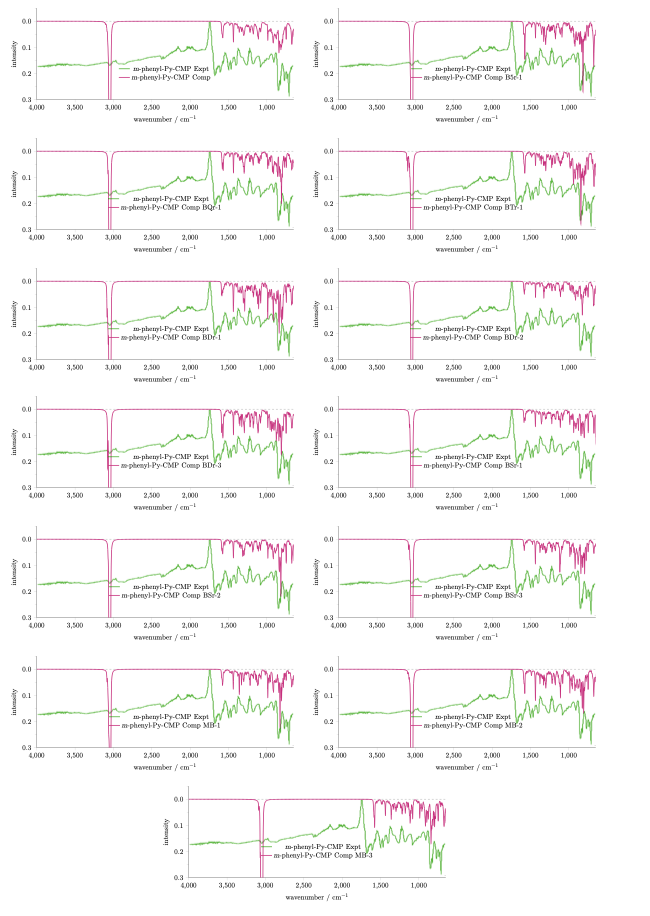


**Figure S25.** IR spectra of experimental (green) and computational (pink)

***m*-phenyl-Py-CMP**. *Key*: Expt – experimental, Comp – computational, considering the whole subset, Comp B5r-1 – quintuple ring structure 1, Comp BQr-1 – quadruple ring structure 1, Comp BTr-1 – triple ring structure 1, Comp BDr-X – double ring structure X, Comp BSr-X – single ring structure X, Comp MB-X – multiply branched structure X.

**
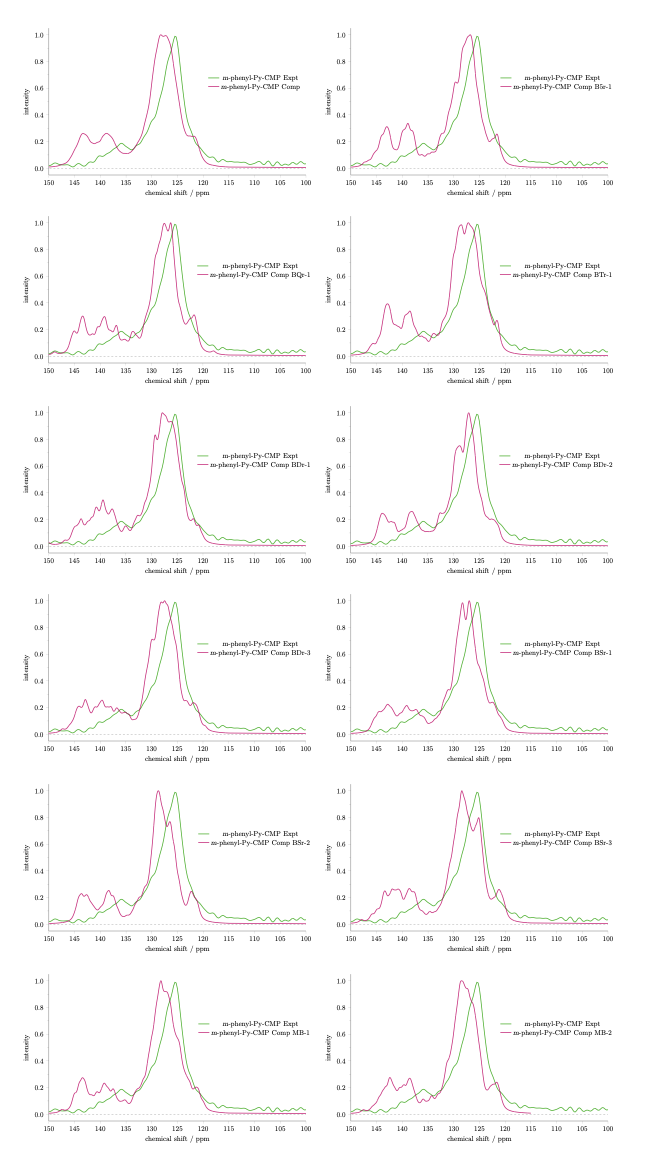
**

**Figure S26.** NMR spectra of experimental (green) and computational (pink)

***m*-phenyl-Py-CMP**. *Key*: Expt – experimental, Comp – computational, considering the whole subset, Comp B5r-1 – quintuple ring structure 1, Comp BQr-1 – quadruple ring structure 1, Comp BTr-1 – triple ring structure 1, Comp BDr-X – double ring structure X, Comp BSr-X – single ring structure X, Comp MB-X – multiply branched structure X.

**
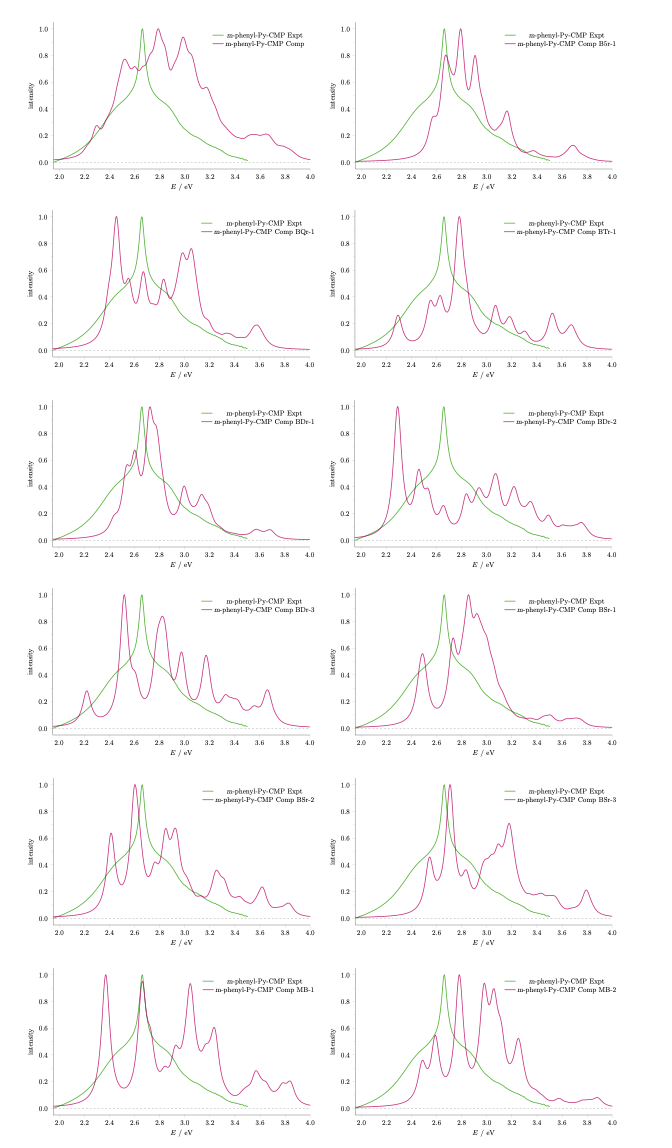
**

**Figure S27.** UV–vis spectra of experimental (green) and computational (pink)

***m*-phenyl-Py-CMP**. *Key*: Expt – experimental, Comp – computational, considering the whole subset, Comp B5r-1 – quintuple ring structure 1, Comp BQr-1 – quadruple ring structure 1, Comp BTr-1 – triple ring structure 1, Comp BDr-X – double ring structure X, Comp BSr-X – single ring structure X, Comp MB-X – multiply branched structure X.

***o*‑phenyl‑Py‑CMP**

**
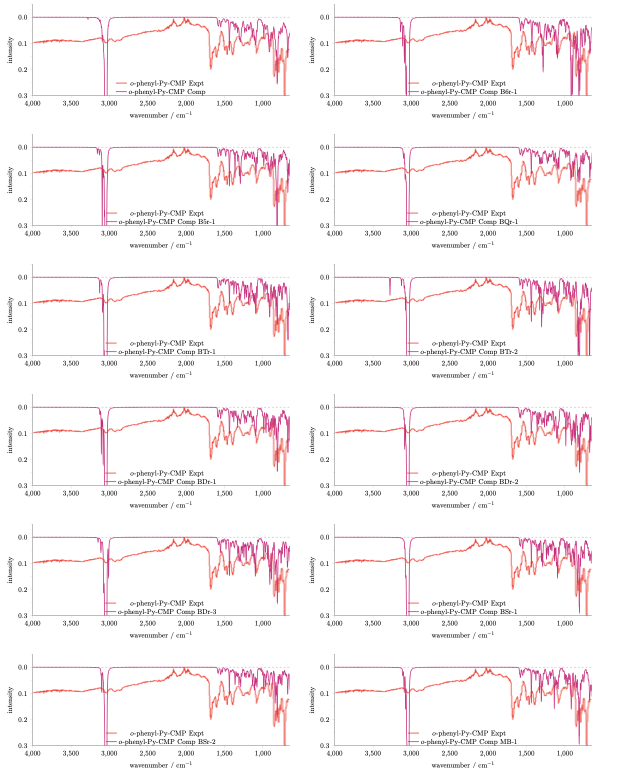
**

**Figure S28.** IR spectra of experimental (red) and computational (pink) ***o*‑phenyl‑Py‑CMP**. *Key*: Expt – experimental, Comp – computational, considering the whole subset,

Comp B6r-1 – sextuple ring structure 1, Comp B5r-1 – quintuple ring structure 1,

Comp BQr-1 – quadruple ring structure 1, Comp BTr-X – triple ring structure X, Comp BDr-X – double ring structure X, Comp BSr-X – single ring structure X, Comp MB-1 – multiply branched structure 1.

**
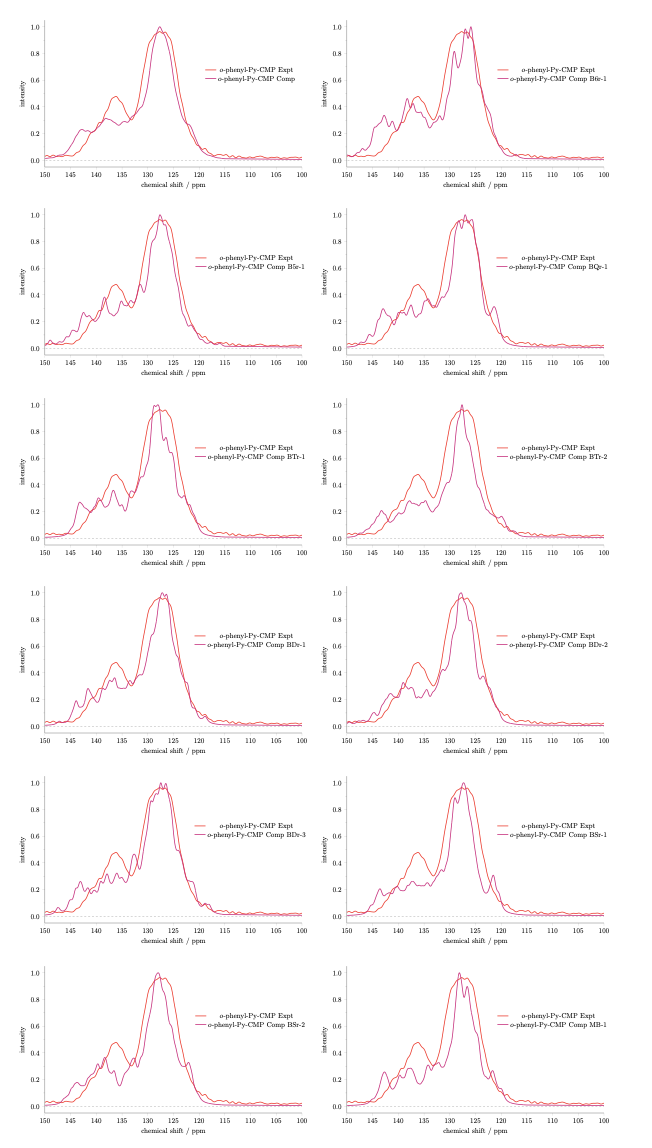
**

**Figure S29.** NMR spectra of experimental (red) and computational (pink) ***o*‑phenyl‑Py‑CMP**. *Key*: Expt – experimental, Comp – computational, considering the whole subset, Comp B6r-1 – sextuple ring structure 1, Comp B5r-1 – quintuple ring structure 1, Comp BQr-1 – quadruple ring structure 1, Comp BTr-X – triple ring structure X, Comp BDr-X – double ring structure X, Comp BSr-X – single ring structure X, Comp MB-1 – multiply branched structure 1.

**
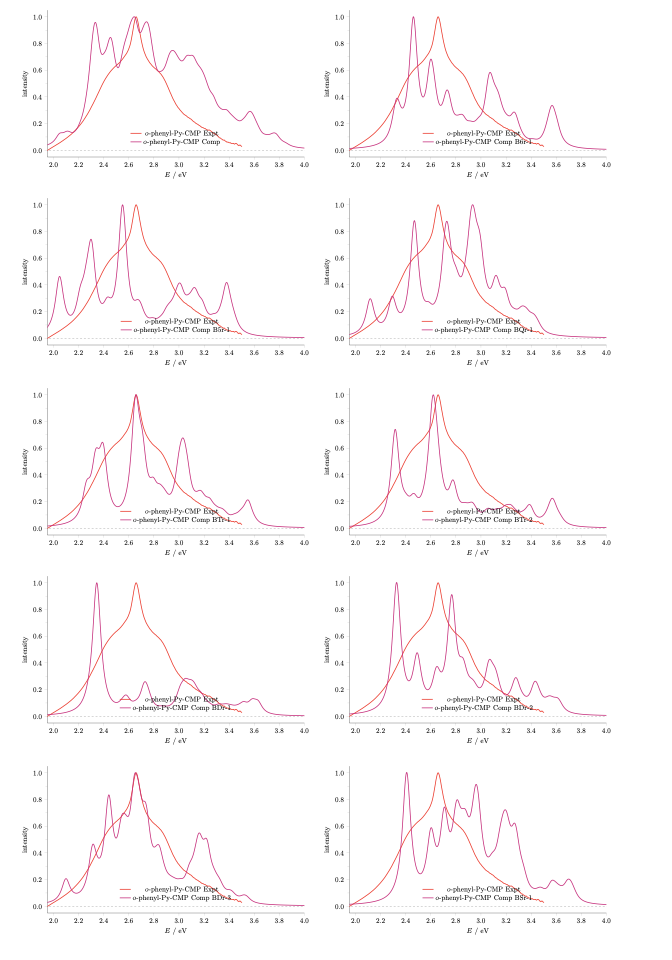
**

**Figure S30.** UV–vis spectra of experimental (red) and computational (pink) ***o*‑phenyl‑Py‑CMP**. *Key*: Expt – experimental, Comp – computational, considering the whole subset, Comp B6r-1 – sextuple ring structure 1, Comp B5r-1 – quintuple ring structure 1, Comp BQr-1 – quadruple ring structure 1, Comp BTr-X – triple ring structure X, Comp BDr-X – double ring structure X, Comp BSr-X – single ring structure X, Comp MB-1 – multiply branched structure 1.

**Figure S31.** Comparison of the experimental spectra of ***x*-phenyl-Py-CMP** and weighted (computational) averages of 10% ***x*-phenyl-Py-CMP** to 90% **Py-CMP**. (a) IR, ***x*** = ***p***,

(b) NMR, ***x*** = ***p***, (c) UV–vis, ***x*** = ***p***, (d) IR, ***x*** = ***m***, (e) NMR, ***x*** = ***m***, (f) UV–vis, ***x*** = ***m***,

(g) IR, ***x*** = ***o***, (h) NMR, ***x*** = ***o***, (i) UV–vis, ***x*** = ***o***.

*Key*: experimental ***p*-phenyl-Py-CMP** – purple, experimental ***m*-phenyl-Py-CMP** – green, experimental ***o*‑phenyl‑Py‑CMP** – red, weighted computational spectra – pink.

**Figure S32.** Comparison of the experimental spectra of ***x*-phenyl-Py-CMP** and weighted (computational) averages of 20% ***x*-phenyl-Py-CMP** to 80% **Py-CMP**. (a) IR, ***x*** = ***p***,

(b) NMR, ***x*** = ***p***, (c) UV–vis, ***x*** = ***p***, (d) IR, ***x*** = ***m***, (e) NMR, ***x*** = ***m***, (f) UV–vis, ***x*** = ***m***,

(g) IR, ***x*** = ***o***, (h) NMR, ***x*** = ***o***, (i) UV–vis, ***x*** = ***o***.

*Key*: experimental ***p*-phenyl-Py-CMP** – purple, experimental ***m*-phenyl-Py-CMP** – green, experimental ***o*‑phenyl‑Py‑CMP** – red, weighted computational spectra – pink.

**Figure S33.** Comparison of the experimental spectra of ***x*-phenyl-Py-CMP** and weighted (computational) averages of 30% ***x*-phenyl-Py-CMP** to 70% **Py-CMP**. (a) IR, ***x*** = ***p***,

(b) NMR, ***x*** = ***p***, (c) UV–vis, ***x*** = ***p***, (d) IR, ***x*** = ***m***, (e) NMR, ***x*** = ***m***, (f) UV–vis, ***x*** = ***m***,

(g) IR, ***x*** = ***o***, (h) NMR, ***x*** = ***o***, (i) UV–vis, ***x*** = ***o***.

*Key*: experimental ***p*-phenyl-Py-CMP** – purple, experimental ***m*-phenyl-Py-CMP** – green, experimental ***o*‑phenyl‑Py‑CMP** – red, weighted computational spectra – pink.

**Figure S34.** Comparison of the experimental spectra of ***x*-phenyl-Py-CMP** and weighted (computational) averages of 40% ***x*-phenyl-Py-CMP** to 60% **Py-CMP**. (a) IR, ***x*** = ***p***,

(b) NMR, ***x*** = ***p***, (c) UV–vis, ***x*** = ***p***, (d) IR, ***x*** = ***m***, (e) NMR, ***x*** = ***m***, (f) UV–vis, ***x*** = ***m***,

(g) IR, ***x*** = ***o***, (h) NMR, ***x*** = ***o***, (i) UV–vis, ***x*** = ***o***.

*Key*: experimental ***p*-phenyl-Py-CMP** – purple, experimental ***m*-phenyl-Py-CMP** – green, experimental ***o*‑phenyl‑Py‑CMP** – red, weighted computational spectra – pink.

**Figure S35.** Comparison of the experimental spectra of ***x*-phenyl-Py-CMP** and weighted (computational) averages of 50% ***x*-phenyl-Py-CMP** to 50% **Py-CMP**. (a) IR, ***x*** = ***p***,

(b) NMR, ***x*** = ***p***, (c) UV–vis, ***x*** = ***p***, (d) IR, ***x*** = ***m***, (e) NMR, ***x*** = ***m***, (f) UV–vis, ***x*** = ***m***,

(g) IR, ***x*** = ***o***, (h) NMR, ***x*** = ***o***, (i) UV–vis, ***x*** = ***o***.

*Key*: experimental ***p*-phenyl-Py-CMP** – purple, experimental ***m*-phenyl-Py-CMP** – green, experimental ***o*‑phenyl‑Py‑CMP** – red, weighted computational spectra – pink.

1. **Density-difference plots to characterise excited states**

The excited states for each cluster, as predicted by TDA-CAM-B3LYP/def2-SVP calculations, typically involve multiple important orbital transitions (this simply means the excited states are in each case best represented by contributions from several occupied–virtual orbital pairs). To most intuitively examine the nature of the excited states, we have generated *density difference plots* for a range of individual excited states in three representative **Py-CMP** clusters; MB-1, BSr-4, and BDr-3, illustrating the different types of excitations seen in branched and MCR-based clusters.

Initially, the ground state one-electron density $\rho_{S_{0}}(\mathbf{r})$, and the excited state one-electron density associated with the $n$th excited state of interest $\rho_{S_{n}}(\mathbf{r})$ (in all cases the “relaxed” density is calculated) are calculated using Gaussian 09. To evaluate the density difference, defined as the difference between the excited state density and the ground state density, $\rho_{S_{n}}\left( \mathbf{r} \right)-\rho_{S_{0}}(\mathbf{r})$, the densities are each converted to discrete grid data using the Gaussian utility program *cubegen*, and then the densities at each grid point are subtracted, using the Gaussian utility program *cubeman*. The resultant “density difference” cube files are then plotted using GaussView. In the figures below, the magenta colour represents the “electron”; where the electron has been excited to, and the blue/cyan colour represents the “hole”; where the electron has been excited from.

**MB-1 density difference plots:**


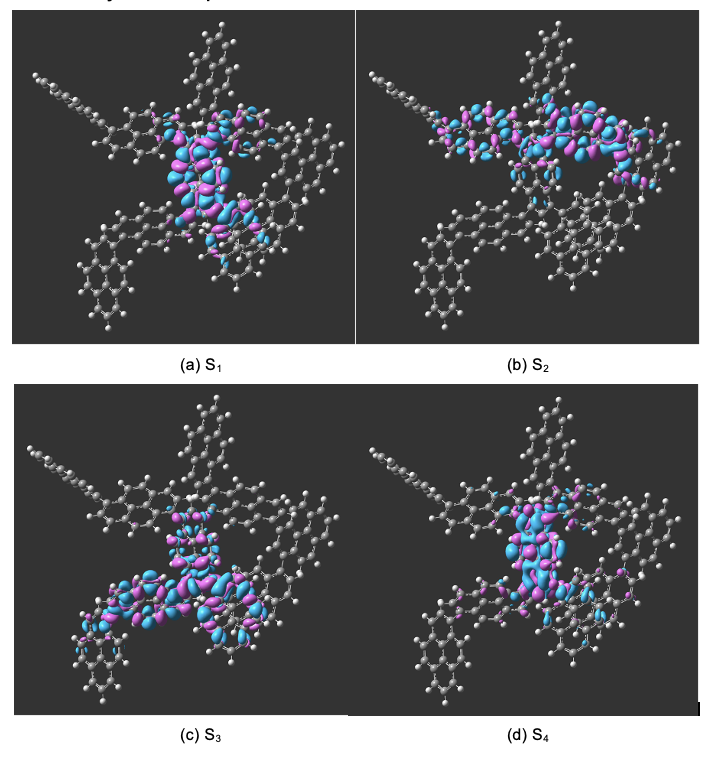


**Figure S36.** Several example density-difference plots for MB-1.

**BSr-4 density difference plots:**


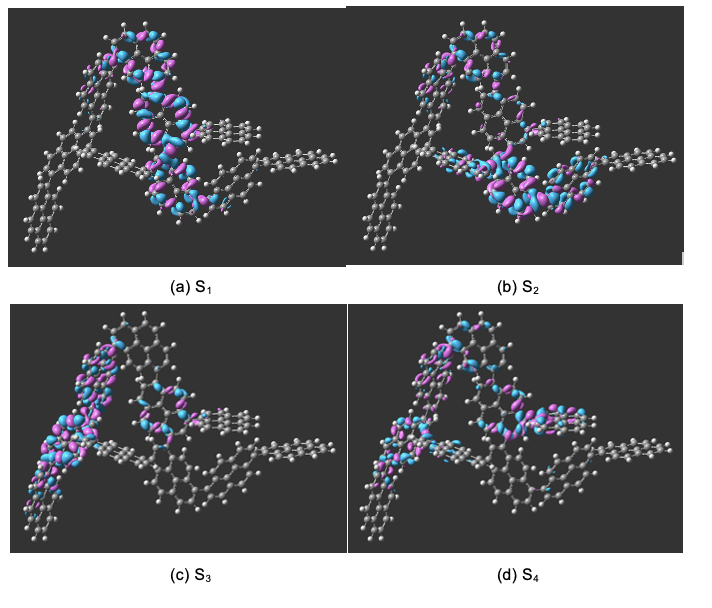


**Figure S37.** Several example density-difference plots for BSr-4.

**BDr-3 density difference plots:**


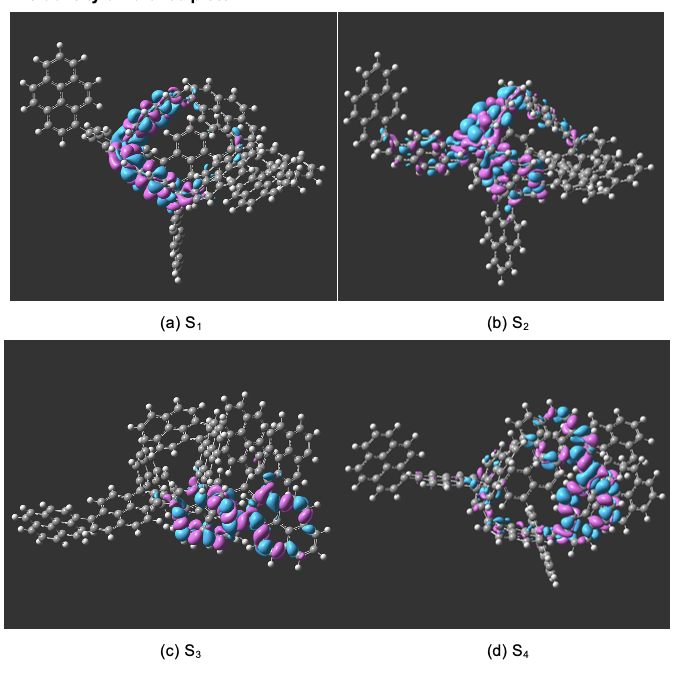


**Figure S38.** Several example density-difference plots for BDr-3.

**Figure S39.** An annotated UV–vis spectrum of BDr-3, indicating the calculated excited states that contribute to each energy range of the spectrum. Density difference plots are given for each of the lowest 30 excited states in Figure S40.


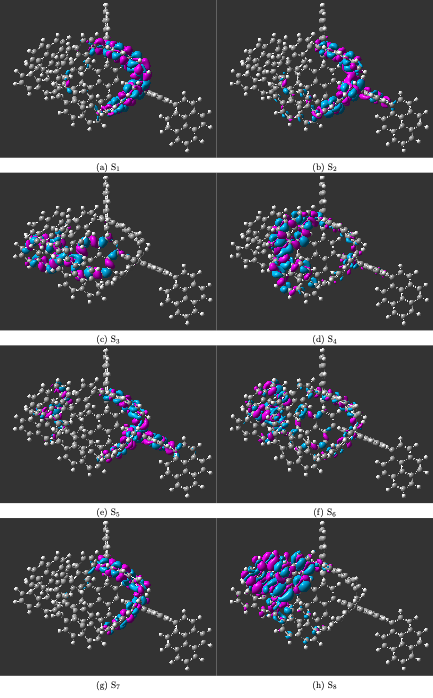


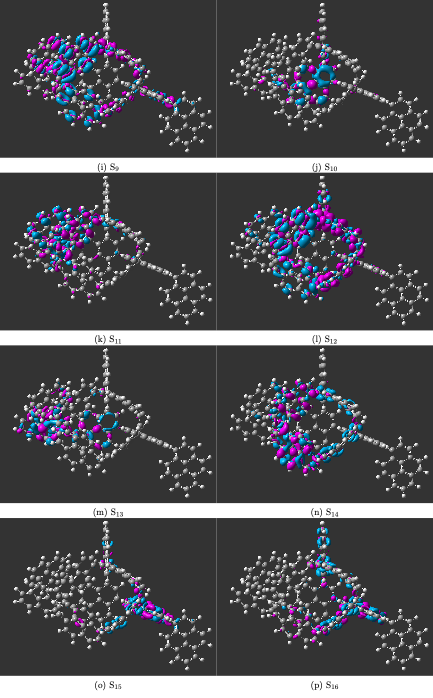


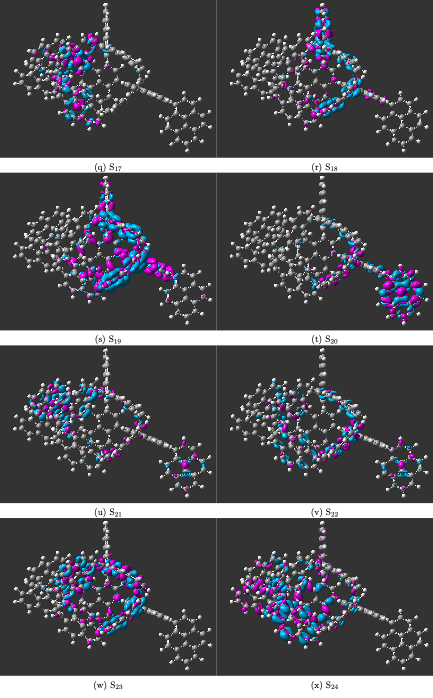


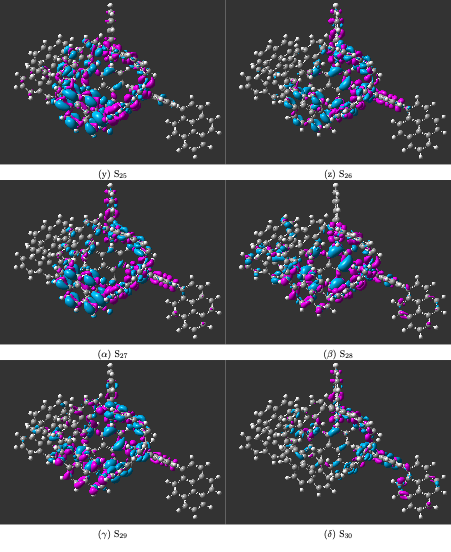


**Figure S40.** Density difference plots for each of these lowest 30 excited states of cluster BDr-3 (as shown as contributing to the overall UV–vis spectrum in Figure S39).

1. **Influence of density on UV–vis spectra**

In this section, the ‘occupied space’ of each cluster within the subset for each material is compared. As a proxy for the volume occupied by each cluster, we use values derived from the electronic spatial extent reported within Gaussian 09 from CAM-B3LYP/def2-SVP calculations, at xTB geometries. We compare the value for each cluster relative to the electronic spatial extent of a linear chain composed of ten pyrene units. The relative electronic spatial extent of each is given by the ‘ESE’ value in Figures S41–S44.

The electronic spatial extent (ESE) can be used as a simple measure of molecular volume, as it increases with the spread of the electron density throughout a system. We therefore use the ESE as it roughly correlates with the density of the structure; those systems (of equivalent composition) with a higher ESE will correspond to lower-density structures, whereas those with a lower ESE will correspond to higher-density structures. In the work here, we consider ratios of the ESE relative to the 10-unit “linear” Py chain that we consider earlier in this work (section S6) as a measure of the density (via the contraction in the ESE volume relative to the **10Py** chain as a structure becomes more dense).

Firstly, the subset structures of each material are shown as a function of increasing density (corresponding to decreasing ESE). Following this, we begin with the convoluted UV–vis spectra of the entirety of the subset for each material, and systematically remove the ‘least dense’ cluster from the overall combined spectrum one-by-one until we are left with only the densest cluster for each of our subsets. This allows us to assess the influence of structure density on the predicted spectrum. Finally, two histograms are plotted for each material. The first shows the density (number) of excited states within each 0.05 eV energy bin of the UV–vis spectrum. The second shows the cumulative intensity of the number of states within each energy bin, accounting for the relative oscillator strength of each contributing excited state. For example, if there are two excited states within a particular energy bin, the oscillator strengths of the two excited states are summed to give the cumulative intensity associated with that energy bin. The plots are then normalised in order the maximum experimental intensity is equal to the maximum computational cumulative intensity.


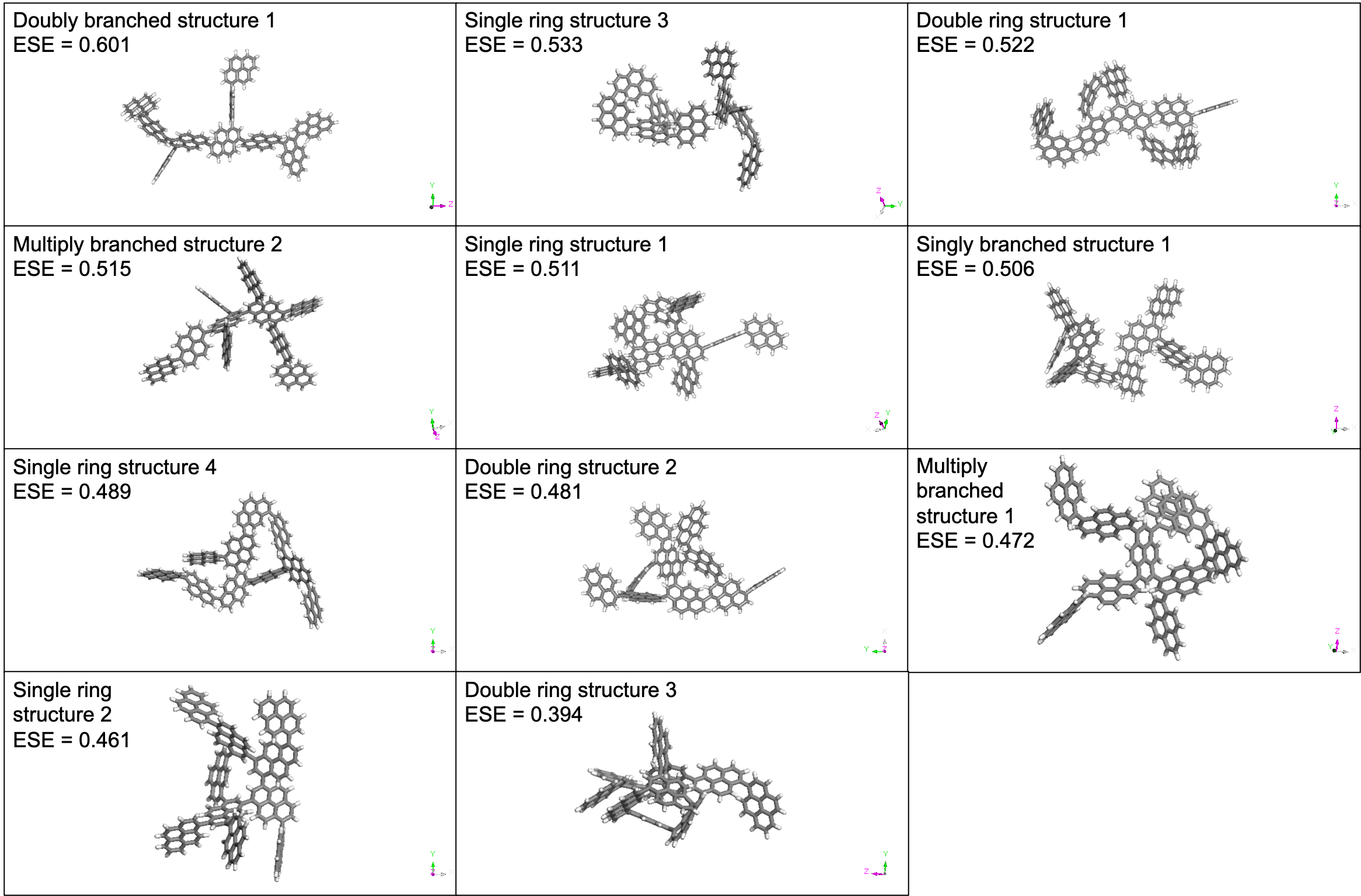


**Figure S41.** Structures included within the subset for **Py-CMP**, going from the least dense (highest ESE) to the densest (lowest ESE).


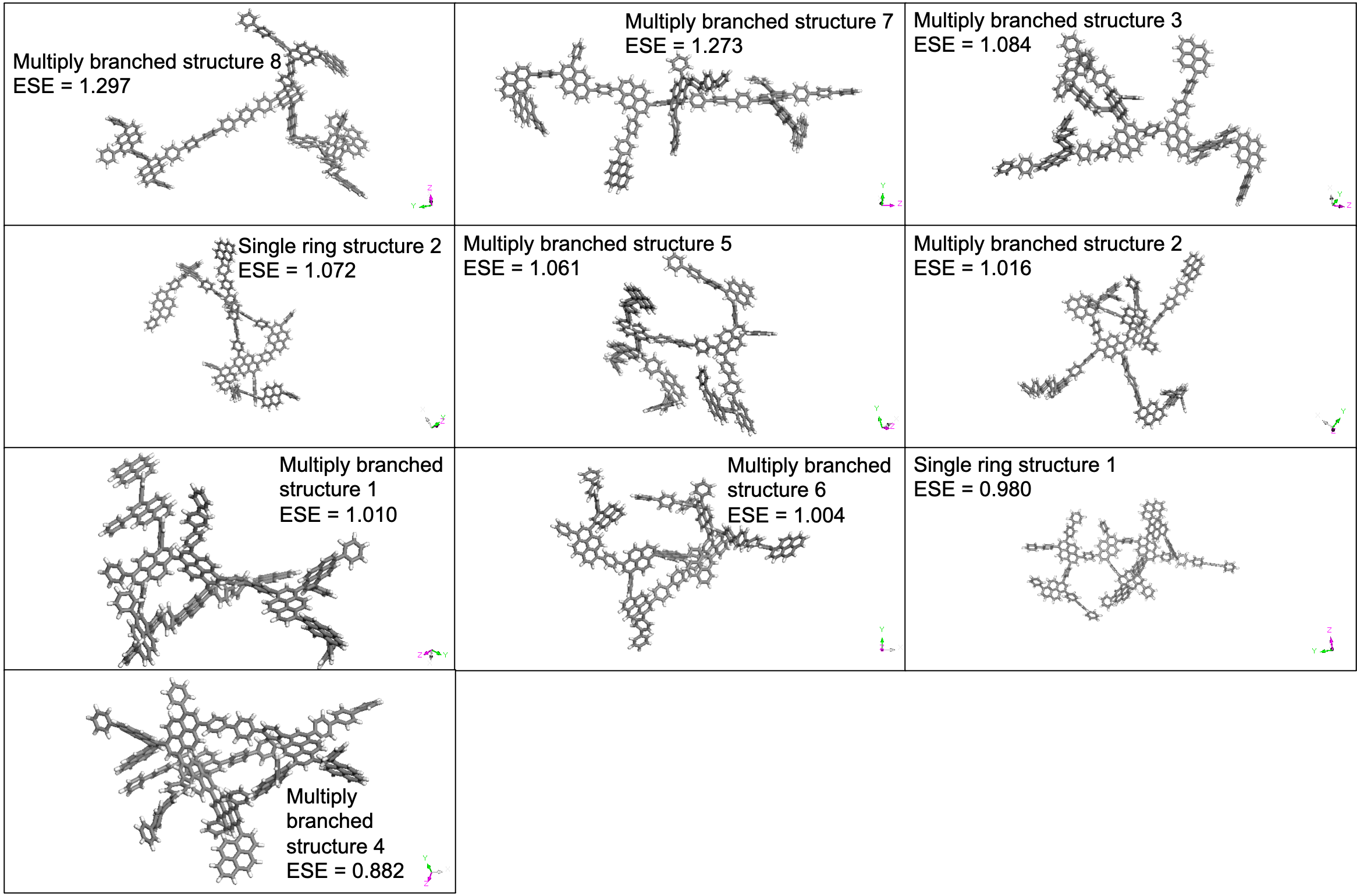


**Figure S42.** Structures included within the subset for ***p*-phenyl-Py-CMP**, going from the (highest ESE) to the densest (lowest ESE).

**
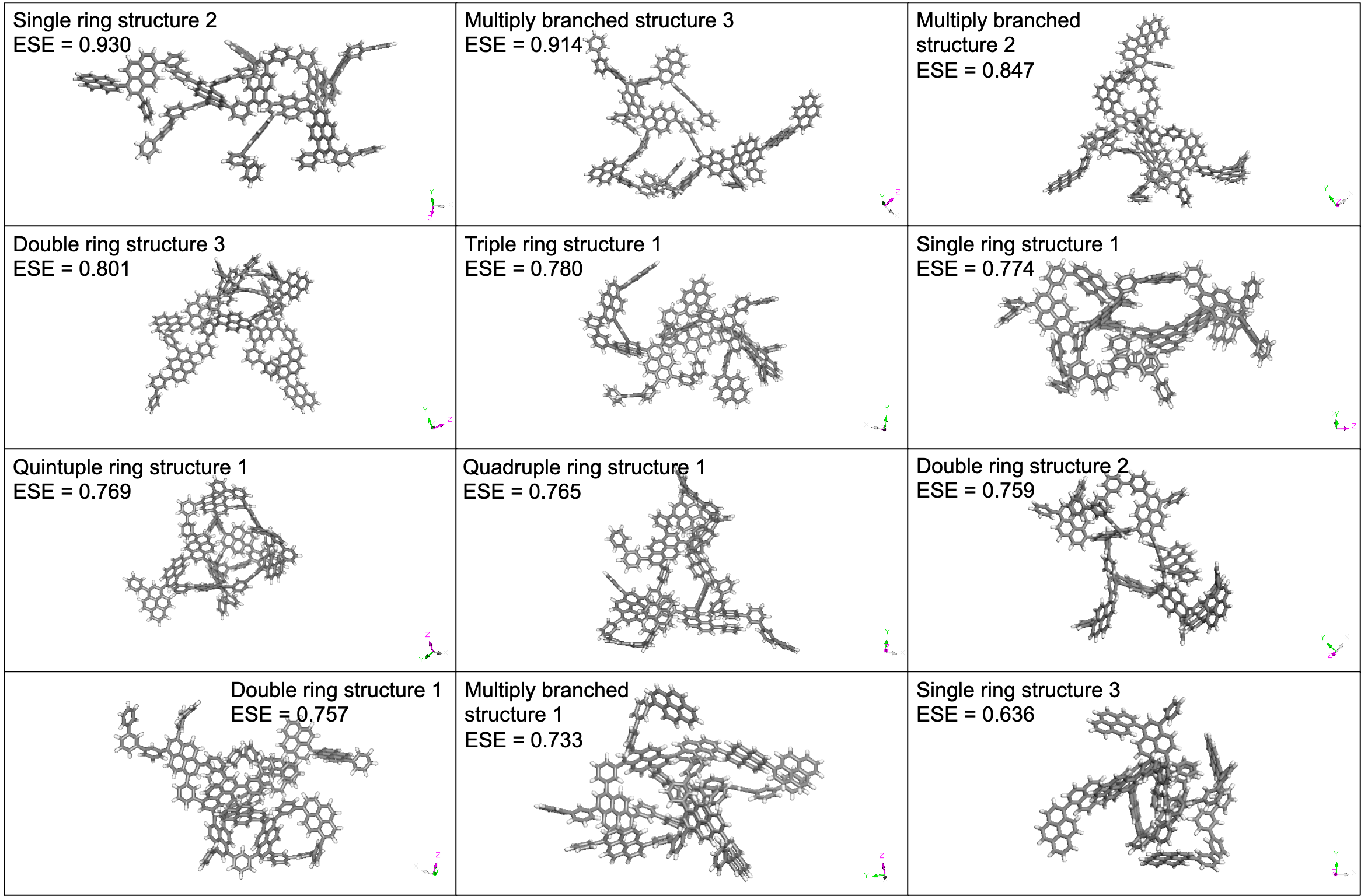
**

**Figure S43.** Structures included within the subset for ***m*-phenyl-Py-CMP**, going from the (highest ESE) to the densest (lowest ESE).


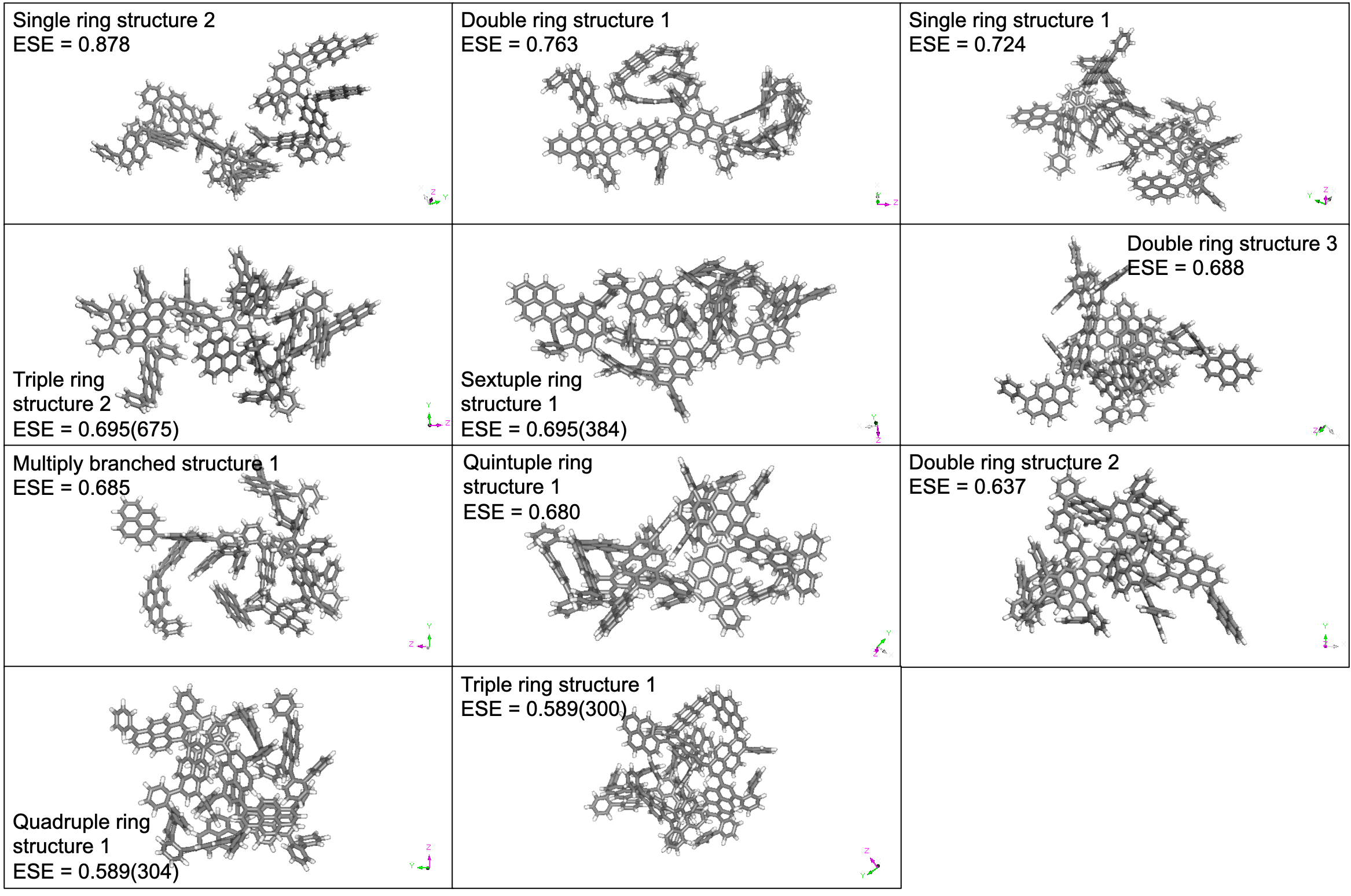


**Figure S44.** Structures included within the subset for ***o*‑phenyl‑Py‑CMP**, going from the (highest ESE) to the densest (lowest ESE).

**UV–vis**

Initial plot – *all clusters*


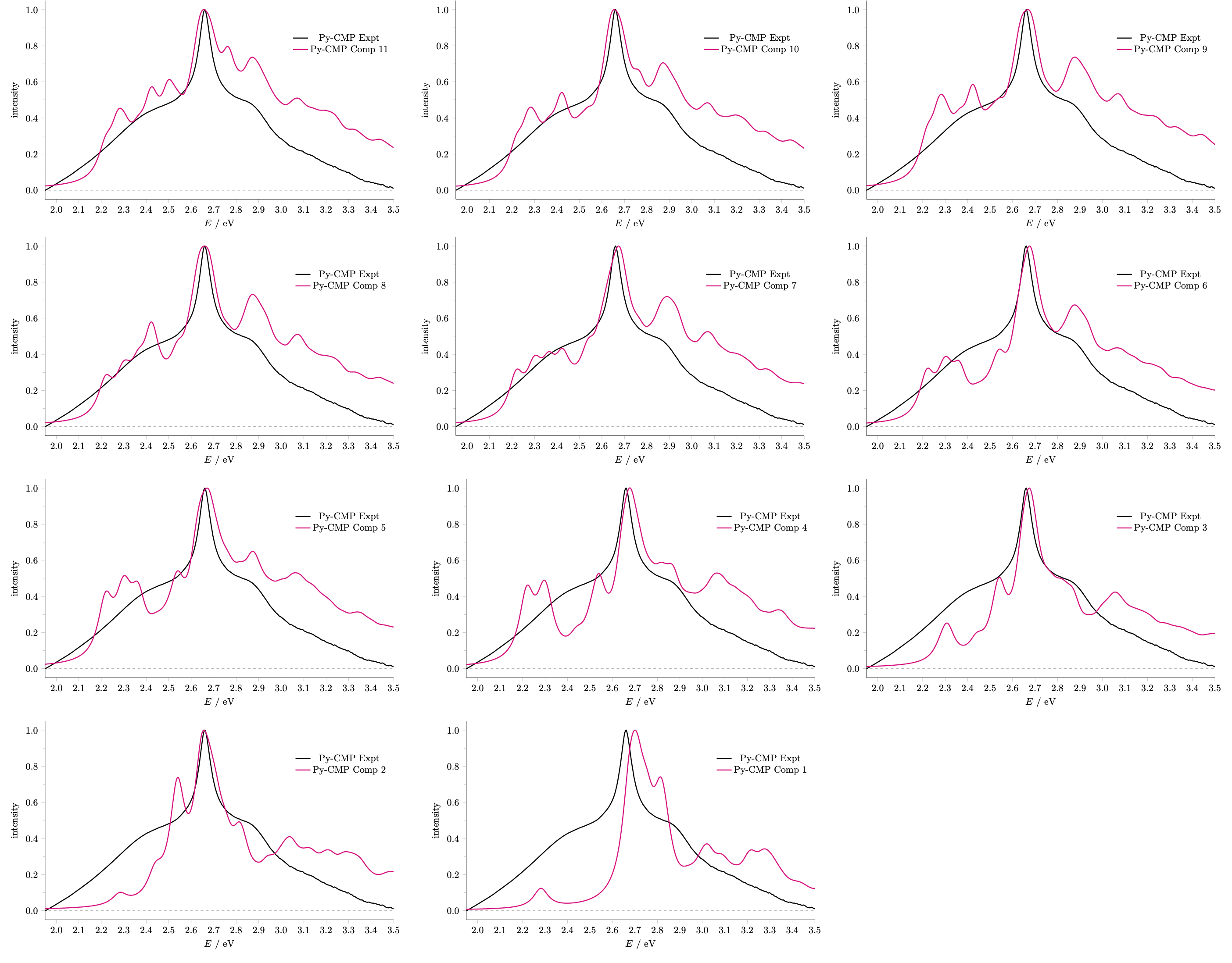


Final plot – *highest density cluster only*

**Figure S45.** Combined, broadened UV–vis spectra of the **Py-CMP** subset, with the least dense structure systematically removed one-by-one in each case. *Key*: Experimental spectrum – black, computational spectrum – pink, where ‘Comp X’ indicates the X most dense structures in the subset are included within the combined computational spectrum.

**
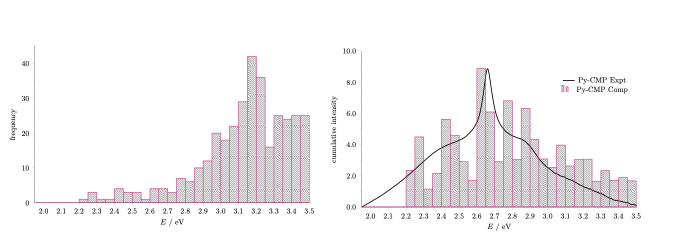
**

**Figure S46.** Histogram showing the density of excited states within the **Py-CMP** subset (left) and the cumulative intensity of the number of states within each energy bin, taking into account the relative oscillator strength of each excited state, normalised so the maximum experimental intensity is equal to the maximum computational cumulative intensity (right). *Key*: Experimental spectrum – black line, computational histograms – grey and pink bars.

Initial plot – *all clusters*


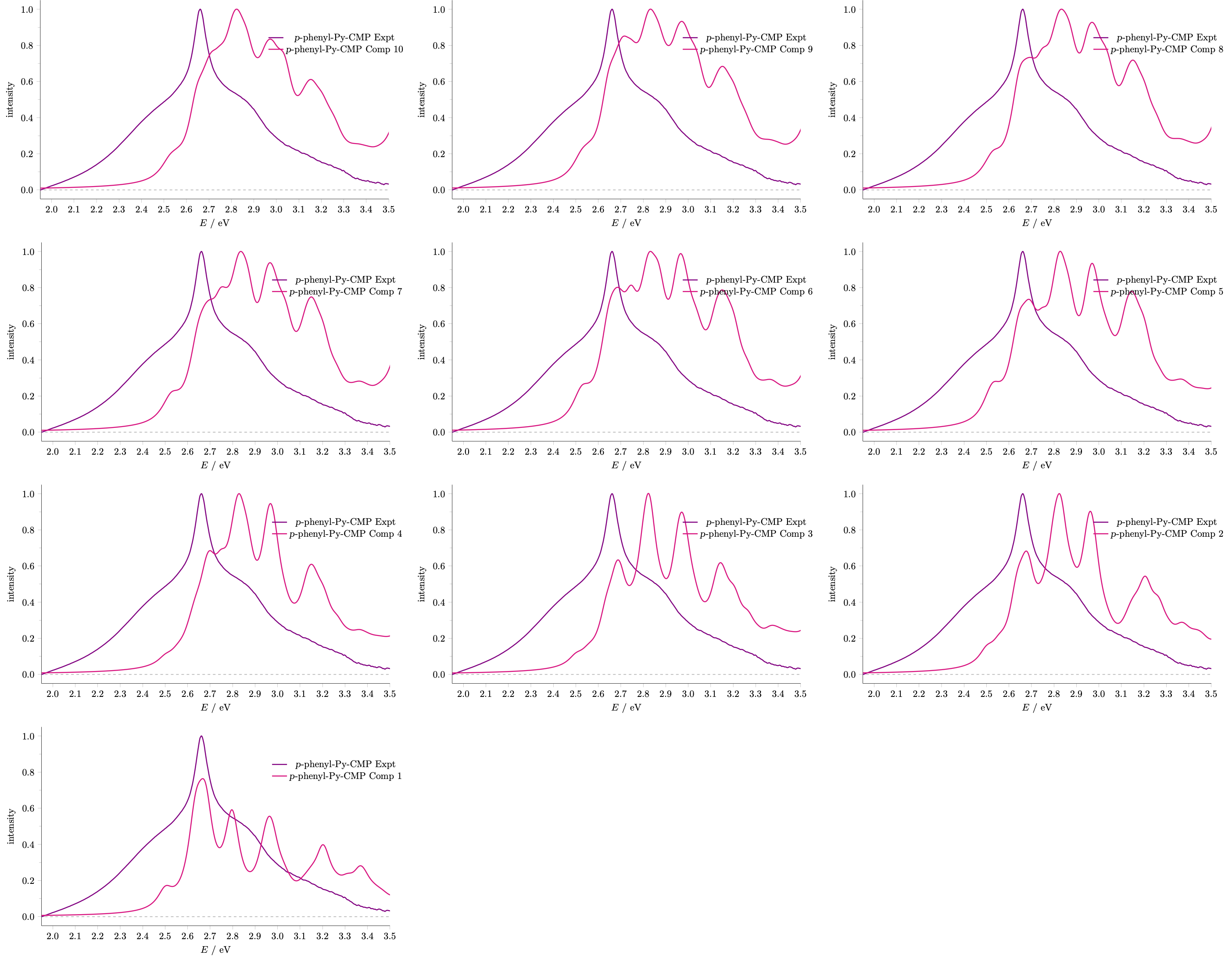


Final plot – *highest density cluster only*

**Figure S47.** Combined, broadened UV–vis spectra of the ***p*-phenyl-Py-CMP** subset, with the least dense structure systematically removed one-by-one in each case. *Key*: Experimental spectrum – purple, computational spectrum – pink, where ‘Comp X’ indicates the X most dense structures in the subset are included within the combined computational spectrum.


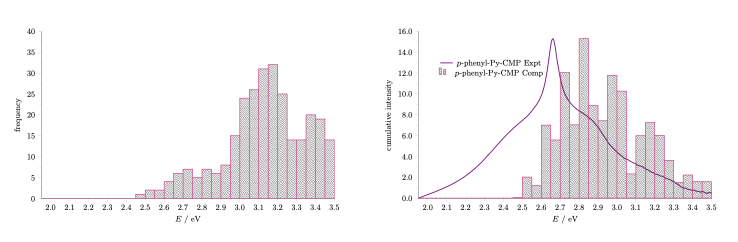


**Figure S48.** Histograms showing the density of excited states within the ***p*-phenyl-Py-CMP** subset (left) and the cumulative intensity of the number of states within each energy bin, normalised so the maximum experimental intensity is equal to the maximum computational cumulative intensity (right). *Key*: Experimental spectrum – purple line, computational histograms – grey and pink bars.

Initial plot – *all clusters*


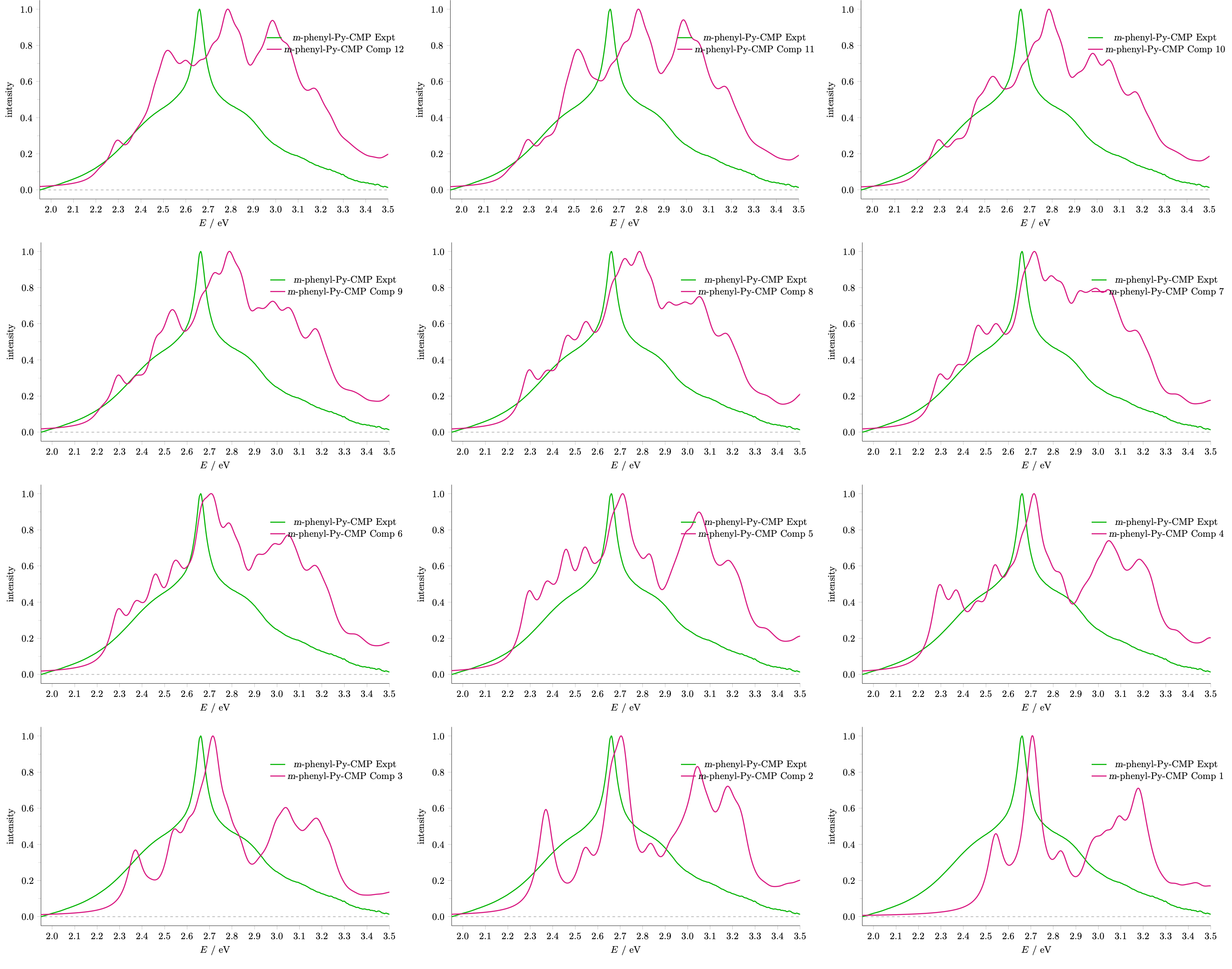


Final plot – *highest density cluster only*

**Figure S49.** Combined, broadened UV–vis spectra of the ***m*-phenyl-Py-CMP** subset, with the least dense structure systematically removed one-by-one in each case. *Key*: Experimental spectrum – green, computational spectrum – pink, where ‘Comp X’ indicates the X most dense structures in the subset are included within the combined computational spectrum.

**Figure S50.** Histograms showing the density of excited states within the ***m*-phenyl-Py-CMP** subset (left) and the cumulative intensity of the number of states within each energy bin, normalised so the maximum experimental intensity is equal to the maximum computational cumulative intensity (right). *Key*: Experimental spectrum – green line, computational histograms – grey and pink bars.

Initial plot – *all clusters*


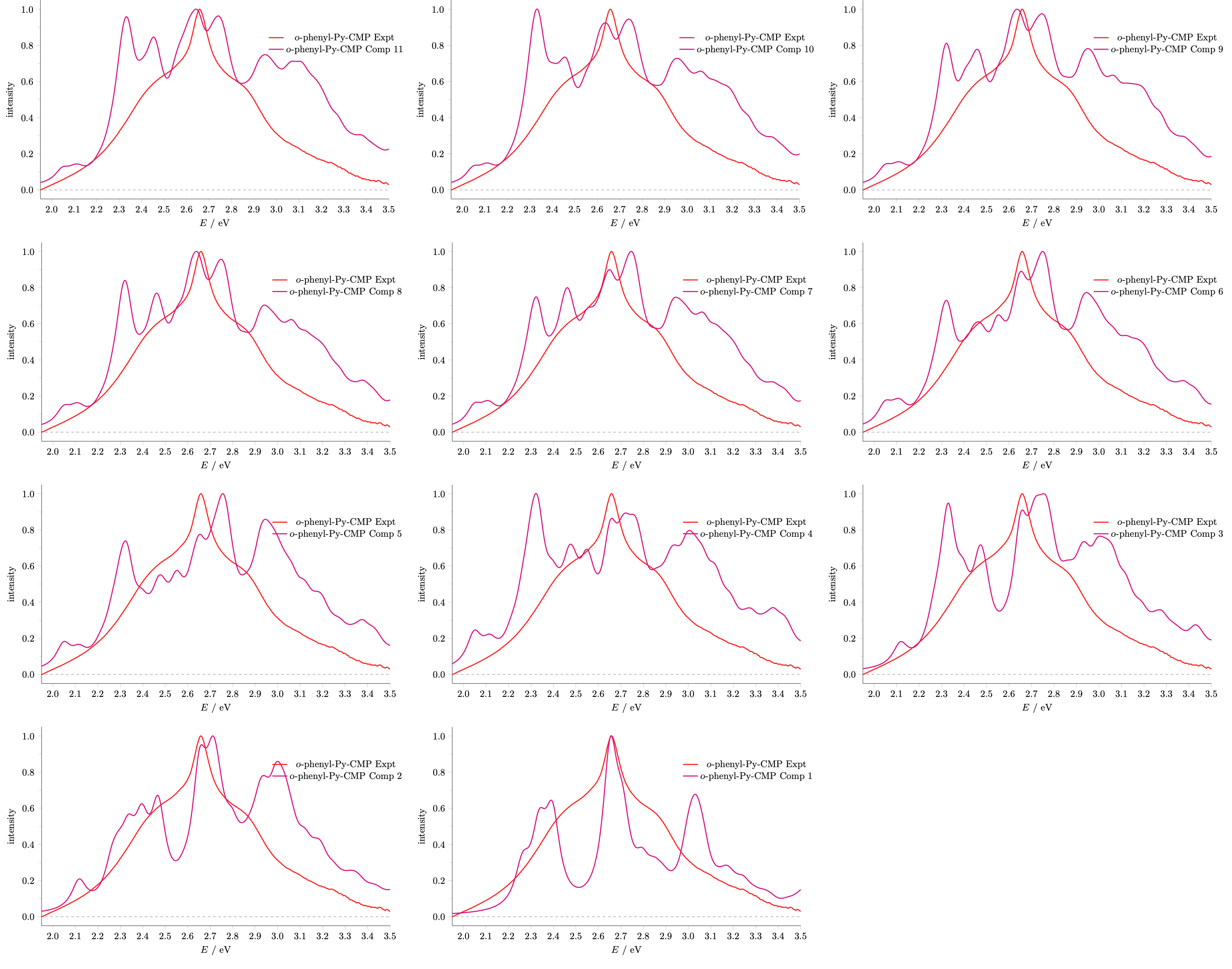


Final plot – *highest density cluster only*

**Figure S51.** Combined, broadened UV–vis spectra of the ***o*‑phenyl‑Py‑CMP** subset, with the least dense structure systematically removed one-by-one in each case. *Key*: Experimental spectrum – red, computational spectrum – pink, where ‘Comp X’ indicates the X most dense structures in the subset are included within the combined computational spectrum.


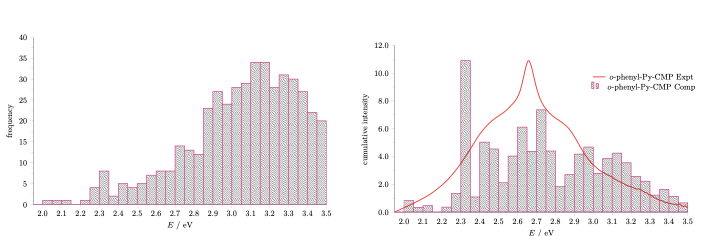


**Figure S52.** Histograms showing the density of excited states within the ***o*‑phenyl‑Py‑CMP** subset (left) and the cumulative intensity of the number of states within each energy bin, normalised so the maximum experimental intensity is equal to the maximum computational cumulative intensity (right). *Key*: Experimental spectrum – red line, computational histograms – grey and pink bars.

1. **References**

1 Yamamoto, T. *et al.* Preparation of π-conjugated poly(thiophene-2,5-diyl), poly(p-phenylene), and related polymers using zerovalent nickel complexes. Linear structure and properties of the π-conjugated polymers. *Macromolecules* **25**, 1214-1223 (1992). <https://doi.org:10.1021/ma00030a003>

2 Jiang, J.-X., Trewin, A., Adams, D. J. & Cooper, A. I. Band gap engineering in fluorescent conjugated microporous polymers. *Chem. Sci.* **2**, 1777-1781 (2011). <https://doi.org:10.1039/C1SC00329A>

3 Fayon, P., Thomas, J. M. H. & Trewin, A. Structure and Properties of a Nanoporous Supercapacitor. *J. Phys. Chem. C* **120**, 25880-25891 (2016). <https://doi.org:10.1021/acs.jpcc.6b08712>

4 Liu, A., Mollart, C., Trewin, A., Fan, X. & Lau, C. H. Photo-Modulating CO2 Uptake of Hypercross-linked Polymers Upcycled from Polystyrene Waste. *ChemSusChem* **16**, e202300019 (2023). <https://doi.org:https://doi.org/10.1002/cssc.202300019>

5 Mollart, C., Holcroft, S., Peach, M. J. G., Rowling, A. & Trewin, A. Artificial synthesis of covalent triazine frameworks for local structure and property determination. *Phys. Chem. Chem. Phys.* **24**, 20025-20029 (2022). <https://doi.org:10.1039/D2CP02430F>

6 Thomas, J. M. H. *et al.* Artificial Synthesis of Conjugated Microporous Polymers via Sonogashira–Hagihara Coupling. *J. Phys. Chem. B* **124**, 7318-7326 (2020). <https://doi.org:10.1021/acs.jpcb.0c04850>

7 Thomas, J. M. H. & Trewin, A. Amorphous PAF-1: Guiding the Rational Design of Ultraporous Materials. *J. Phys. Chem. C* **118**, 19712-19722 (2014). <https://doi.org:10.1021/jp502336a>

8 Anderson, J. A., Lorenz, C. D. & Travesset, A. General purpose molecular dynamics simulations fully implemented on graphics processing units. *J. Comput. Phys.* **227**, 5342-5359 (2008). <https://doi.org:https://doi.org/10.1016/j.jcp.2008.01.047>

9 Glaser, J. *et al.* Strong scaling of general-purpose molecular dynamics simulations on GPUs. *Comput. Phys. Commun.* **192**, 97-107 (2015). <https://doi.org:https://doi.org/10.1016/j.cpc.2015.02.028>

10 Sun, H. Ab initio calculations and force field development for computer simulation of polysilanes. *Macromolecules* **28**, 701-712 (1995). <https://doi.org:10.1021/ma00107a006>

11 Clark, S. J. *et al.* First principles methods using CASTEP. *Z. für Kristallogr. - Cryst. Mater.* **220**, 567-570 (2005). <https://doi.org:doi:10.1524/zkri.220.5.567.65075>

12 Hohenberg, P. & Kohn, W. Inhomogeneous Electron Gas. *Phys. Rev.* **136**, B864-B871 (1964). <https://doi.org:10.1103/PhysRev.136.B864>

13 Kohn, W. & Sham, L. J. Self-Consistent Equations Including Exchange and Correlation Effects. *Phys. Rev.* **140**, A1133-A1138 (1965). <https://doi.org:10.1103/PhysRev.140.A1133>

14 Payne, M. C., Teter, M. P., Allan, D. C., Arias, T. A. & Joannopoulos, J. D. Iterative minimization techniques for ab initio total-energy calculations: molecular dynamics and conjugate gradients. *Rev. Mod. Phys.* **64**, 1045-1097 (1992). <https://doi.org:10.1103/RevModPhys.64.1045>

15 Profeta, M., Mauri, F. & Pickard, C. J. Accurate First Principles Prediction of 17O NMR Parameters in SiO2:  Assignment of the Zeolite Ferrierite Spectrum. *J. Am. Chem. Soc.* **125**, 541-548 (2003). <https://doi.org:10.1021/ja027124r>

16 Pfrommer, B. G., Côté, M., Louie, S. G. & Cohen, M. L. Relaxation of Crystals with the Quasi-Newton Method. *J. Comput. Phys.* **131**, 233-240 (1997). <https://doi.org:https://doi.org/10.1006/jcph.1996.5612>

17 Byrd, R. H., Nocedal, J. & Schnabel, R. B. Representations of quasi-Newton matrices and their use in limited memory methods. *Math. Prog.* **63**, 129-156 (1994). <https://doi.org:10.1007/BF01582063>

18 Perdew, J. P., Burke, K. & Ernzerhof, M. Generalized Gradient Approximation Made Simple. *Phys. Rev. Lett.* **77**, 3865-3868 (1996). <https://doi.org:10.1103/PhysRevLett.77.3865>

19 Francis, G. P. & Payne, M. C. Finite basis set corrections to total energy pseudopotential calculations. *J. Phys.: Condens. Matter* **2**, 4395-4404 (1990). <https://doi.org:10.1088/0953-8984/2/19/007>

20 Yates, J. R., Pickard, C. J. & Mauri, F. Calculation of NMR chemical shifts for extended systems using ultrasoft pseudopotentials. *Phys. Rev. B* **76**, 024401 (2007). <https://doi.org:10.1103/PhysRevB.76.024401>

21 Bonhomme, C. *et al.* First-Principles Calculation of NMR Parameters Using the Gauge Including Projector Augmented Wave Method: A Chemist’s Point of View. *Chem. Rev.* **112**, 5733-5779 (2012). <https://doi.org:10.1021/cr300108a>

22 Pickard, C. J. & Mauri, F. All-electron magnetic response with pseudopotentials: NMR chemical shifts. *Phys. Rev. B* **63**, 245101 (2001). <https://doi.org:10.1103/PhysRevB.63.245101>

23 Monkhorst, H. J. & Pack, J. D. Special points for Brillouin-zone integrations. *Phys. Rev. B* **13**, 5188-5192 (1976). <https://doi.org:10.1103/PhysRevB.13.5188>

24 Pracht, P., Grant, D. F. & Grimme, S. Comprehensive Assessment of GFN Tight-Binding and Composite Density Functional Theory Methods for Calculating Gas-Phase Infrared Spectra. *J. Chem. Theory Comput.* **16**, 7044--7060 (2020). <https://doi.org:10.1021/acs.jctc.0c00877>

25 Zwijnenburg, M. A. Elucidating the Microscopic Origin of the Unique Optical Properties of Polypyrene. *J. Phys. Chem. C* **116**, 20191-20198 (2012).

26 Salvador, S. & Chan, P. FastDTW: Toward Accurate Dynamic Time Warping in Linear Time and Space. *Intell. Data Anal.* **11**, 561-580 (2007).

27 Salvador, S. & Chan, P. *Toward Accurate Dynamic Time Warping in Linear Time and Space*. Vol. 11 (2004).

28 Crawford, A. G. *et al.* Experimental and Theoretical Studies of the Photophysical Properties of 2- and 2,7-Functionalized Pyrene Derivatives. *Journal of the American Chemical Society* **133**, 13349--13362 (2011). <https://doi.org:10.1021/ja2006862>
